# Genetic diversity and population structure of Kudouzi (*Sophora alopecuroides*) in Northwest China revealed by SNP markers and seed phenotypic traits

**DOI:** 10.3389/fpls.2025.1634581

**Published:** 2025-09-04

**Authors:** Cunkai Luo, Fanyan Ma, Panxin Niu, Zhao Zhang, Weiting Chen, Ping Jiang, Mei Wang, Guangming Chu, Xiang Huang

**Affiliations:** ^1^ Agricultural College, Shihezi University, Shihezi, China; ^2^ Xinjiang Yuli National Positioning Observation and Research Station for Desert Ecosystems, Yuli, China; ^3^ Bozhou Water Conservancy Irrigation Test Station, Bole, China

**Keywords:** *Sophora alopecuroides*, single nucleotide polymorphism, genetic diversity, seed phenotypic traits, population structure, redundancy analysis

## Abstract

**Introduction:**

*Sophora alopecuroides* L., a perennial leguminous herb native to northwest China, holds medicinal, ecological, and forage value. However, intensified human activities have caused a sharp decline in its wild populations and genetic diversity.

**Methods:**

To assess its genetic structure and diversity, we analyzed 65 wild populations using SNP markers and seed phenotypic traits.

**Results:**

The coefficient of variation for eight seed traits ranged from 2.87% to 7.94%, with diversity indices (*H*) from 1.639 to 1.767, indicating rich phenotypic variation. Clustering based on phenotypic traits and SNP data both divided populations into two main groups. Genetic diversity was relatively low (*He* = 0.22; *Ho* = 0.17; P*i* = 0.19), and AMOVA showed that variation was mainly among individuals (132.83%), with low population differentiation (*F*_ST_ = 0.00-0.04). Redundancy analysis revealed that phenotypic traits were largely influenced by mean temperature of the driest quarter and annual wind speed, while genetic diversity was shaped by precipitation and thermal variables.

**Conclusions:**

Our results provide a foundation for understanding the genetic variation of *S. alopecuroides*, offering valuable insights for its conservation and breeding programs.

## 1 Introduction

*Sophora alopecuroides* L., a perennial herb of the Leguminosae family, is widely distributed across arid and semi-arid regions in West and Central Asia. In China, it is primarily found in Xinjiang, Gansu, Ningxia, and Inner Mongolia (Rong et al., 2020; Wang et al., 2020). Studies have shown that *S. alopecuroides* exhibits strong root development, drought resistance, tolerance to salinity and alkalinity, and nitrogen fixation capability (Wang et al., 2020; Li et al., 2023). Consequently, it has become a vital pioneer plant for environmental protection in northwest China due to its strong drought resistance, alkalinity tolerance, and resistance to wind and sand. In addition to its ecological significance, *S. alopecuroides* possesses substantial medicinal and agricultural value. Its seeds, aerial parts, and roots contain bioactive compounds, including alkaloids, flavonoids, volatile oils, steroids, polysaccharides, and free fatty acids (Gao et al., 2012; Huang et al., 2016; Aly et al., 2019; Huang et al., 2024b). These compounds support its use in traditional medicine and potential applications in biological pesticides (Qi et al., 2018), green manure (Ma et al., 2018), and high-quality forage (Yan et al., 2023). In natural populations, *S. alopecuroides* reproduces both sexually and asexually. Although detailed studies on its reproductive biology are limited, related species in the Sophora genus are typically monoecious, insect-pollinated, and self-compatible (Wang et al., 2019b). Additionally, *S. alopecuroides* exhibits strong clonal propagation through its root system, which facilitates its persistence and spread in harsh environments (Liu et al., 1996; Yang, 2005). These reproductive characteristics provide a crucial biological foundation for further investigation of its genetic diversity and population structure.

Genetic diversity and population structure play a key role in species conservation, utilization and breeding (Caroline et al., 2016). Gao et al. (Gao, 2013) reported that the concentrations of key medicinal compounds, such as oxymatrine and total flavonoids, vary significantly among *S. alopecuroides* seeds from different geographical regions. Similarly, An et al. (An JL Li et al., 2023) found that the contents of genistein and sophoridine in seeds from northern Xinjiang were generally higher than those from southern Xinjiang. Furthermore, gene flow between different *S. alopecuroides* populations is limited, resulting in high genetic differentiation between populations (Yang et al., 2013). Despite its ecological and medicinal importance, few fundamental studies have been conducted on the large-scale population resources and genetic diversity of *S. alopecuroides*.

Seed traits are crucial for population reproduction and conservation, serving as important indicators of species inheritance (Zhang et al., 2017; Andrea et al., 2021). Phenotypic plasticity, defined as the ability of a single genotype to produce different phenotypes in varying environments, is largely a result of genetic selection and is closely linked to phenotypic diversity (Nicotra et al., 2010; Pfennig et al., 2010; Hu et al., 2015). Significant differences in seed traits, such as size and thousand-grain weight, among different wild populations of *S. alopecuroides* suggest diverse ecological clusters and complex genetic backgrounds (Yang et al., 2010). However, the relationship between seed phenotypic traits and genetic diversity in *S. alopecuroides* has not been thoroughly investigated.

Genotyping-by-Sequencing (GBS) is a high-throughput, next-generation sequencing method that reduces genomic complexity through the use of restriction enzymes. This technique generates high-density single nucleotide polymorphism (SNP) markers by tagging random genomic deoxyribonucleic acid (DNA) fragments common across samples with unique short DNA sequences (barcodes) and pooling samples into a single sequencing run, enabling the detection and labeling of mutations at a relatively low cost (Elshire et al., 2011). Currently, GBS serves as a powerful genotyping tool, widely used in genetic mapping, marker-assisted selection (Geleta et al., 2020; Lu et al., 2023), diversity analysis, and germplasm and species identification (Lee et al., 2019; Al et al., 2022). Among molecular markers, SNPs are key tools in population and quantitative genetics. SNPs represent single-base differences among individuals of the same species and are the most abundant form of genetic variation (Uddin et al., 2024). As third-generation markers, SNPs offer advantages such as biallelic nature, high density, genetic stability, ease of detection, and genome-wide distribution (Liu et al., 2019; Rayda et al., 2022). With advances in sequencing and bioinformatics, SNPs have been extensively used to study genetic diversity, population structure, and differentiation (Li et al., 2014; Basheer-Salimia et al., 2015; Wu and Blair, 2017; Niu et al., 2019; Muli et al., 2022). However, their application in the analysis of genetic diversity in *S. alopecuroides* remains unreported.

In this study, seeds were collected from 65 wild populations of *S. alopecuroides* across various geographic regions. Phenotypic traits were measured, and SNP markers were used to assess the genetic structure and diversity within these populations. The main objectives of this study are as follows: (1) What is the level of genetic diversity and population structure of *S. alopecuroides* in these populations? (2) What are the effects of geographic and environmental factors on the genetic diversity of *S. alopecuroides*? Based on the findings, this research aims to provide scientific basis for the effective conservation, management, and utilization of *S. alopecuroides* germplasm, as well as for the development of high-quality cultivars to meet market demands.

## 2 Materials and methods

### 2.1 Plant materials

The field collection of *S. alopecuroides* samples was approved by the Chinese government and conducted in accordance with all relevant national regulations. Species identification was confirmed by Professor Zhuowen Zhang (College of Horticulture and Forestry, Huazhong Agricultural University), and voucher specimens were deposited in the Department of Forestry, College of Agriculture, Shihezi University (Accession Nos: SHAF20230801–SHAF20230865).

Based on the primary distribution range of *S. alopecuroides* in China (Rong et al., 2024) and the seed maturity period, a total of 65 seed samples were collected from five provinces: Xinjiang, Gansu, Ningxia, Inner Mongolia, and Shaanxi, between August and October 2023 (**Supplementary Table S1**). Each province contains multiple regions, and a typical suitable area (where *S. alopecuroides* grows well and is free from pests and diseases) was selected for setting up a sampling point in each region. Each sampling point was established with a 20m × 20m quadrat, and the latitude and longitude coordinates were recorded using handheld Global Satellite Positioning System (GPS) equipment. During sampling, a 4m × 4m subquadrat was marked at each of the four corners and the center of the quadrat. Then, 3–5 healthy and mature *S. alopecuroides* individuals were randomly selected in each subquadrat. From each plant, 5 to 10 completely dry, gray-brown pods were selected, with each pod containing 3 to 5 mature seeds. Finally, all seeds collected at each sampling point were combined to form a composite sample representing the genetic diversity of the population in the region, resulting in a total of 65 composite samples. After the pods were collected, they were placed in a ventilated nylon net and transported back. The pods were placed in a cool, dry laboratory (15-20°C, 30-40% humidity) and ventilated in the dark. During this period, the water content of the seeds was monitored dynamically using the 105°C constant weight method. Once the seeds were fully dried, they were manually threshed, and the moth-eaten, mildewed, and damaged seeds were removed. Fully mature seeds were obtained through wind cleaning. The seeds were then stored in a refrigerator at 5°C (Huang et al., 2024a).

**Figure 1:**
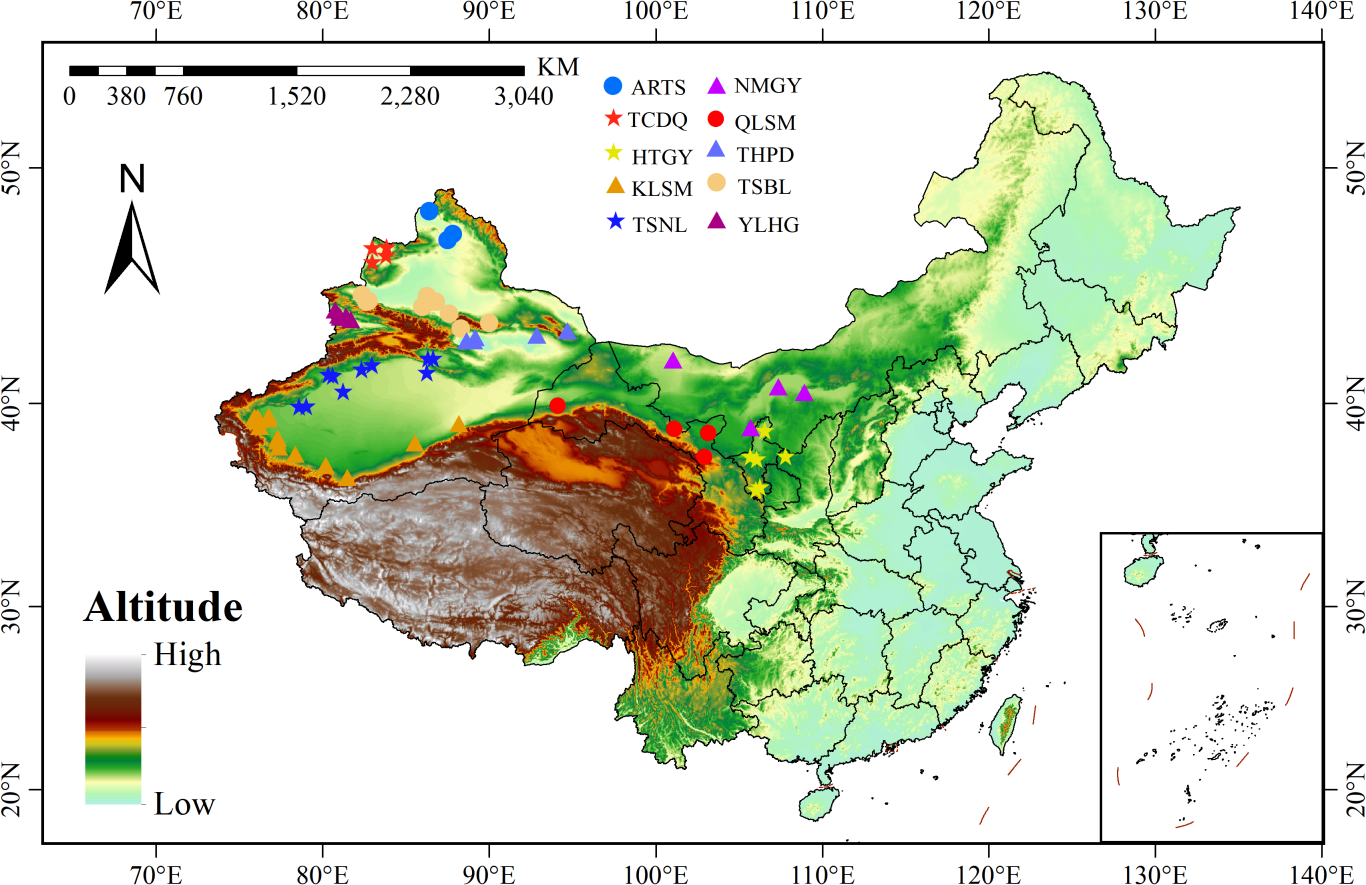
Sampling point distribution map of 10 *Sophora alopecuroides* populations.

Based on the main geographical and topographic features of the 65 regions, such as mountains, basins, and plateaus, the 65 composite samples were divided into 10 populations. Each population corresponds to a distinct geomorphological region, serving as a natural barrier affecting gene flow and population differentiation. These populations are named using abbreviations of representative landforms or regions, including the Qilian Mountains (QLSM), the northern foothills of the Tianshan Mountains (TSBL), the southern foothills of the Tianshan Mountains (TSNL), the Turpan-Hami Basin (THPD), the Altai Mountains (ARTS), the Kunlun Mountains (KLSM), the Ili River Valley (YLHG), the Loess Plateau (HTGY), the Inner Mongolia Plateau (NMGY), and the Tacheng area (TCDQ) (**Figure 1**). Bioclimatic variables were obtained from the WorldCLIM global high-resolution climate database (http://www.worldclim.org/) and local meteorological stations (**Supplementary Tables S2**, **S3**).

### 2.2 Measurement of seed phenotypic traits

A Wanshen SC-G automatic seed analyzer (Hangzhou Wanshen Detection Technology Co., Ltd., China) was used to measure the seed traits of 65 *S. alopecuroides* composite samples, including length, width, perimeter, area, diameter, roundness, and shape index. All traits are quantitative and treated as continuous numerical variables. The SC-G instrument employs high-resolution imaging and digital edge detection technology to extract two-dimensional seed projections (**Supplementary File S1**). The area and perimeter were calculated based on the pixelated outlines of individual seed projections. The roundness was then calculated using the formula: roundness=((4π×area)/perimeter^2^), which quantifies how closely the seed shape resembles a perfect circle. The shape index was computed as the ratio of seed length to width (length/width), reflecting the seed’s elongation. Each index was measured three times, with 200 seeds randomly selected for each repetition, and the mean value was recorded. The thousand-grain weight was measured using a one-thousandth electronic balance, with 1,000 seeds randomly selected each time. Three measurement replicates were conducted, and the average value was recorded (Yang et al., 2010; Wang et al., 2019b).

### 2.3 Statistics and analysis of phenotypic traits

The experimental data were organized using Microsoft Excel 2016, and seed trait data were statistically analyzed using SPSS 27.0 software. Prior to the one-way analysis of variance (ANOVA), the hypothesis of normality and homogeneity of variances was tested. The normality of the data was assessed using the single-sample Kolmogorov-Smirnov test, and the homogeneity of variances was evaluated using the mean-based Levene test. The results indicated that all variables conformed to the hypothesis (*P* > 0.05). Subsequently, one-way analysis of variance (ANOVA) was performed, and mean values at the 0.05 significance level were compared using the least significant difference (LSD) method. Paired Pearson correlation analysis was employed to test the linear relationship between different seed phenotypic traits. The correlation coefficient (r) was calculated, and the significance level was assessed (*P* < 0.05). The results were visualized as relevant heat maps using Origin Pro 2021. The coefficient of variation (*CV*) (Equation 1) and phenotypic genetic Shannon diversity index (*H*) (Equation 2) were calculated in Excel as follows:

The observed values of each trait were classified into 10 levels based on the mean value (X) and standard deviation (SD). Each level represented a 0.5 SD interval. Specifically, Level 1 included values less than X-2SD, and Level 10 included values greater than or equal to X+2SD. Levels 2 to 9 covered the range from X-2SD to X+2SD in 0.5 SD intervals. The relative frequency of each level was then used to calculate *H* (Schneider et al., 2000).

In Equation 2, *i* is the classification of a trait, and Pi is the percentage of the number of materials in the total number of materials in the *i* th grade of the trait.

According to the standardized (z-score) average value of seed phenotypic traits, Origin Pro 2021 was used for hierarchical cluster analysis. The squared Euclidean distance was used as the distance measure, and the average linkage was used as the clustering method. Prior to the analysis, all data were standardized to eliminate the impact of trait dimensions (Di et al., 2024). The standardized z-value calculation formula is as follows:

In Equation 3, X* is the normalized value of z value, *x* is the original value, $\bar{x}$ is the sample mean, and σ is the sample standard deviation.

### 2.4 DNA extraction, GBS library construction and sequencing

Genomic DNA was extracted from 65 *S. alopecuroides* seed samples collected from different provenances using the CTAB method (Murray and Thompson, 1980). DNA integrity was assessed using 1% agarose gel electrophoresis, and concentration and purity were measured with a Nanodrop spectrophotometer and a Qubit fluorometer. For GBS library construction, 0.1-1 μg of DNA per sample was digested with the restriction enzyme MseI, selected based on in silico digestion evaluation to ensure appropriate marker density. Barcoded P1 and P2 adapters, compatible with sticky ends generated by MseI digestion, were ligated to the digested fragments. The adapter-ligated fragments were amplified by PCR to enrich the target regions. Amplified products were pooled and subjected to size selection using agarose gel electrophoresis to recover fragments in the desired size range (~300–500 bp). The libraries were first quantified using a Qubit 2.0 instrument and diluted to 1 ng/μL, followed by insert size assessment using an Agilent 2100 Bioanalyzer. Quantitative PCR (qPCR) was further performed to measure the effective concentration of each library (with >2 nM required). Qualified libraries were pooled based on concentration and subjected to high-throughput sequencing on the Illumina HiSeq platform using a paired-end 150 bp (PE150) strategy.

### 2.5 Data processing and SNP identification

Raw image data from the Illumina platform were converted into FASTQ format using base calling. Raw reads were filtered using the following criteria: (1) reads containing adapter sequences were removed; (2) paired-end reads were discarded if the N content of one read exceeded 10%; and (3) paired-end reads were also removed if more than 50% of the bases in one read had a quality score ≤5. After filtering, high-quality clean reads were retained for downstream analysis. For SNP calling, sample G60-having the largest number of tags-was selected to perform *de novo* clustering using the Stacks pipeline, generating a pseudo-reference sequence. Clean reads from all samples were aligned to this pseudo-reference genome using BWA (v0.7.17) with parameters: mem -t 4 -k 32 -M (Li and Durbin, 2009). Alignment files were sorted using SAMTOOLS (v1.10). SNP calling was performed using SAMTOOLS Mpileup and Bcftools, resulting in 58,448 raw SNPs (Li et al., 2009). SNPs were filtered with a call rate threshold of 0.8 and a minor allele frequency (MAF) ≥ 0.03, resulting in a final dataset of 10,584 high-quality SNP loci for population analysis. See **Supplementary File S2** for detailed SNP site information.

### 2.6 Population structure and diversity analysis

The samples were selected based on the filtered SNP loci, and the distance matrix was calculated using TreeBest (http://treesoft.sourceforge.net/treebest.shtml) software. A pairwise genetic distance matrix was computed using TreeBest based on p-distance, which quantifies the average proportion of nucleotide differences between individual SNP profiles. A phylogenetic tree was subsequently constructed using the neighbor-joining method (Eickmeyer et al., 2008). Feature vectors and eigenvalues were calculated using GCTA (https://yanglab.westlake.edu.cn/software/gcta/), and the PCA distribution map was plotted using the ggplot2 and factoextra packages in R v. 4.4.3. The ADMIXTURE v1.3.0. was used to analyze the population structure (Alexander et al., 2009). First, the input Ped file of PLINK v1.9 was created, and then the population genetic structure and pedigree information were constructed using the admixture software. Arlequin v3.5.2.2 software was used to calculate the genetic diversity indices of each population: observed heterozygosity (Ho), expected heterozygosity (He), molecular variance analysis (AMOVA), and *F*_ST_ between groups to measure potential differences (Schneider et al., 2000). The method of Nei & Li (Nei and Li, 1979) was used to analyze population nucleotide diversity (π) and calculate the single nucleotide diversity index (Pi).

### 2.7 Climatic association analysis

To estimate the extent to which genomic variation is influenced by environmental variables, Redundancy analysis (RDA) was performed using the vegan package v2.6–4 in R v. 4.4.3 to examine the relationships among phenotypic traits, genetic diversity indices, and environmental factors (Oksanen et al., 2025). RDA involves multiple linear regression followed by a PCA on the matrix of regression-fitted values. A dependent matrix of minor allele frequencies for each population and an independent matrix of environmental variables were included. To avoid high collinearity, variables with a VIF greater than 20 were excluded (Borcard et al., 2018).

## 3 Results

### 3.1 Diversity analysis of seed phenotypic traits

**Figure 2:**
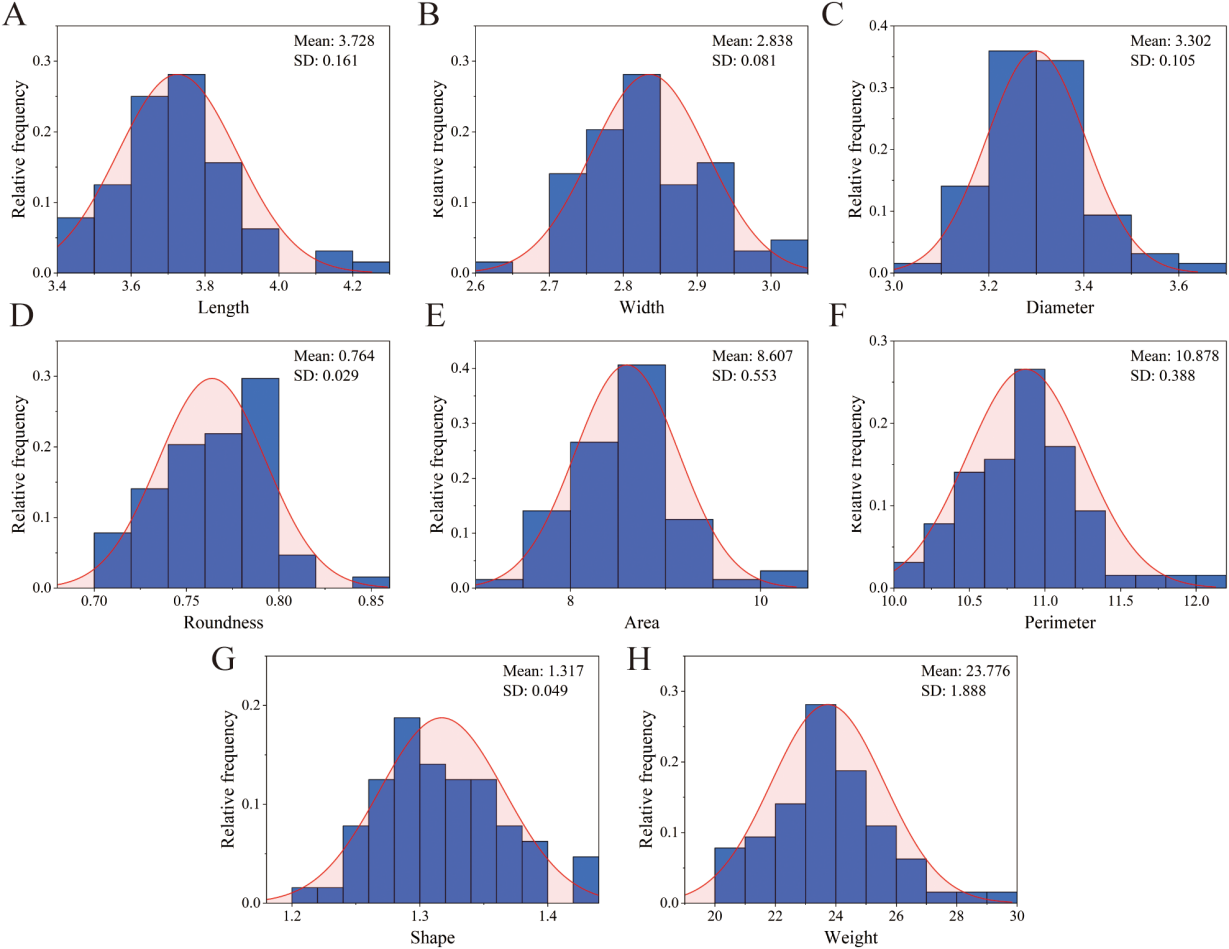
Histogram of frequency distribution of seeds phenotypic traits of *S. alopecuroides*. **(A)** length; **(B)** width; **(C)** diameter; **(D)** roundness; **(E)** shape index; **(F)** perimeter; **(G)** Area; **(H)** thousand-grain weight.

We measured 8 phenotypic traits of 65 *S. alopecuroides* samples and calculated their *CV* and *H* values (**Supplementary Table S4**). The results showed that the *CV* of the 8 phenotypic traits ranged from 2.87% to 7.94%, and the average *CV* was 4.48%. Among these, the *CV* of thousand-grain weight was the largest, while the *CV* of width was the smallest. The diversity indices of the 8 phenotypic traits ranged from 1.639 to 1.767, with an average of 1.713. All indicators followed a normal distributed (**Figure 2**). In order to further confirm the normality of these traits, we conducted a single-sample K-S test. The results showed that all traits obeyed normal distribution (*P* > 0.05). This result indicated that the diversity of the 8 phenotypic traits in the 65 *S. alopecuroides* samples was relatively high.

### 3.2 Correlation analysis of seed phenotypic traits

**Figure 3:**
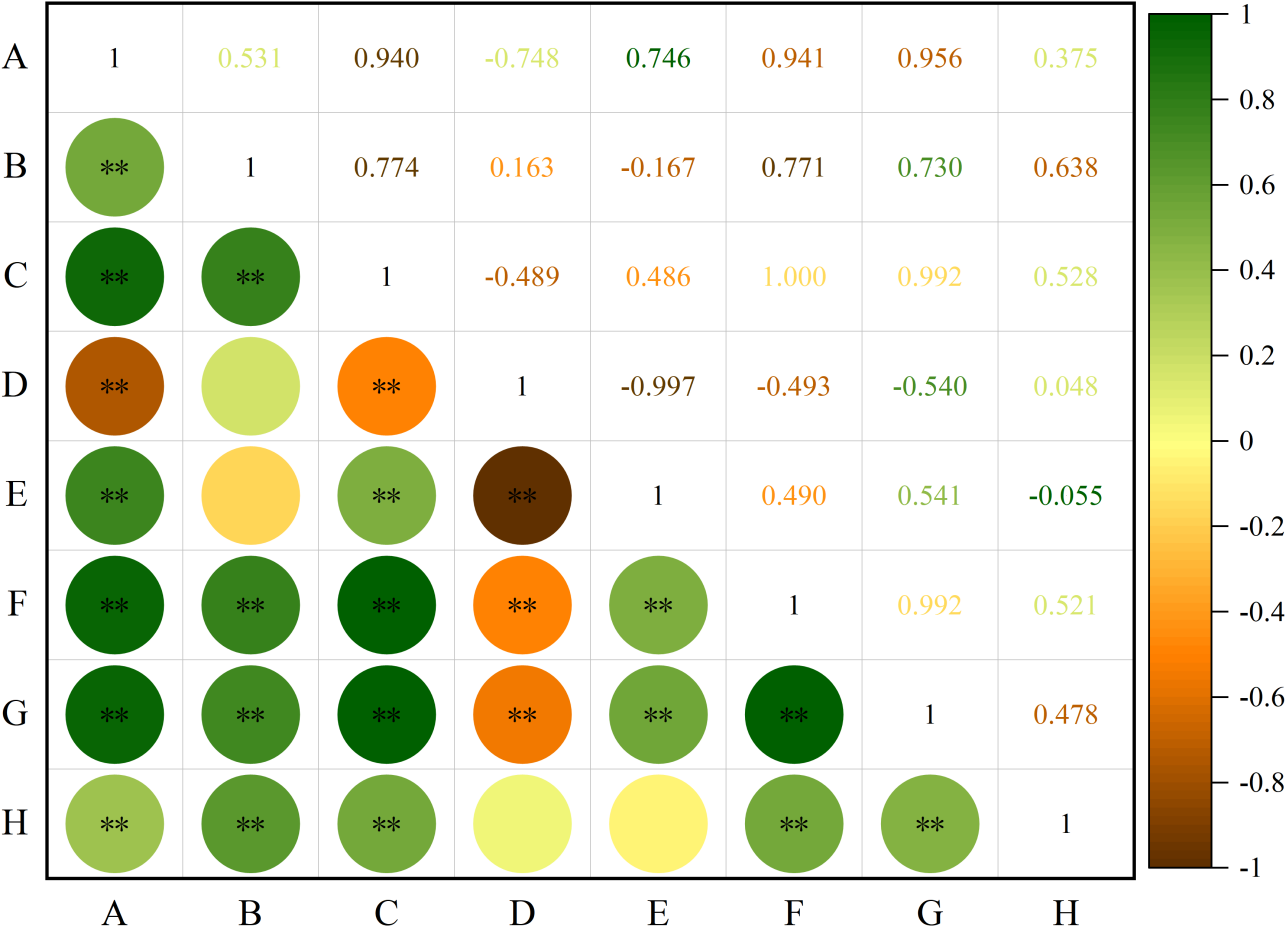
Correlation analysis of seeds phenotypic traits of *S. alopecuroides*. “**” indicates extremely significant correlation (*P* < 0.01); **(A)** length, **(B)** width, **(C)** diameter, **(D)** roundness, **(E)** shape index, **(F)** perimeter, **(G)** area, **(H)** thousand-grain weight.

Correlation analysis was performed on the phenotypic traits of all the tested *S. alopecuroides* seeds (**Figure 3**). The results showed that length was significantly positively correlated with width, diameter, shape index, area, perimeter and thousand-grain weight (*P* < 0.01) but negatively correlated with roundness (*P* < 0.01). Width was significantly positively correlated with diameter, area, perimeter and thousand-grain weight (*P* < 0.01), but weakly correlated with roundness and the shape index. A significant positive correlation was found between diameter and shape index, area, perimeter, and thousand-grain weight (*P* < 0.01) and a significant negative correlation with roundness (*P* < 0.01). Roundness was significantly negatively correlated with the shape index, area, and perimeter (*P* < 0.01), and its correlation with thousand-grain weight was weak. The shape index was significantly positively correlated with area and perimeter (*P* < 0.01), and weakly negatively correlated with thousand-grain weight. Area was significantly positively correlated with perimeter and thousand-grain weight (*P* < 0.01), and thousand-grain weight was also significantly positively correlated with perimeter (*P* < 0.01).

### 3.3 Cluster analysis of seed phenotypic traits

**Figure 4:**
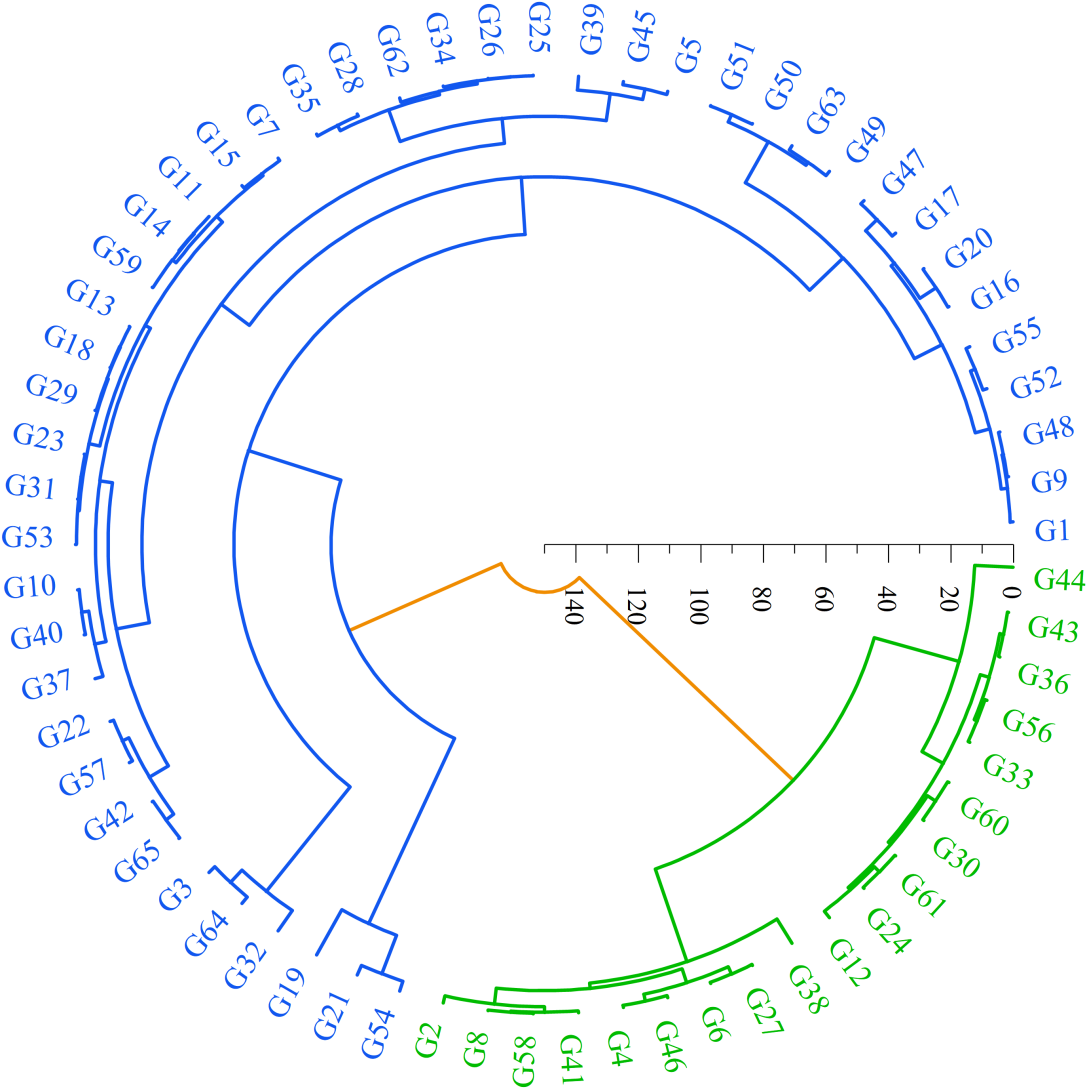
Cluster analysis of phenotypic traits in 65 samples of *S. alopecuroides* seeds. The samples were divided into two main phenotypic groups, group I was blue and group II was green.

Systematic cluster analysis of the seed phenotypic traits of 65 *S. alopecuroides* samples revealed that the samples were divided into two groups (**Figure 4**). Among them, group I is shown in blue in the figure and includes 19 samples from the YLHG, TSBL, KLSM, TSNL, HTGY, and NMGY populations. The samples in this group are characterized by high roundness but smaller values for other traits, indicating that the seeds are small, with light seed weight and poor overall traits. Group II, consisting of 46 samples, is shown in green in the figure and includes samples from all regions. The seeds in this group are generally larger in size, heavier in weight, and perform better in terms of seed length, width, and thousand-grain weight. These characteristics suggest superior seed quality and the potential for higher yield.

### 3.4 Sequencing data analysis

A total of 65 *S. alopecuroides* samples were used for sequencing analysis. The total sequencing data volume was 61.20257 Gb, with an average of 941.578 Mb per sample. The results showed that the sequencing quality was high (Q20 ≥ 93.97%, Q30 ≥ 84.85%), with a normal GC distribution (35.61%-39.78%) (**Supplementary Table S5**). No samples were contaminated by adapter sequences, confirming successful library construction. The sequencing data of 65 *S. alopecuroides* samples were subsequently mapped to the reference genome. The average mapping rate of the population samples ranged from 81.15% to 94.47%, and the average sequencing depth of the genome ranged from 12.87× to 34.92×, with a 1× coverage rate (at least one base coverage) for more than 0.10% of the samples (**Supplementary Table S6**). Next, a total of 58,448 SNP sites were detected using SAMTOOLS, which were filtered to obtain high-quality SNPs. A total of 10,584 high-quality SNPs were obtained for subsequent analysis.

The *S. alopecuroides* samples were divided into 10 clusters based on their geographical origin: YLHG, TSBL, TCDQ, ARTS, KLSM, TSNL, THPD, QLSM, and NMGY, totaling 65 samples. Furthermore, the heterozygosity of each population varied significantly (**Table 1**). The Ho values of the *S. alopecuroides* populations ranged from 0.20037 to 0.23511, with an average of 0.22213. The He ranged from 0.14387 to 0.18852, with an average of 0.16861, and Pi ranged from 0.16442 to 0.19803, with an average of 0.18647. Among them, Ho in the TCDQ was the highest, He in the KLSM was the highest, and Ho, He, and Pi in the NMGY were the lowest. Genetic diversity was highest in the TCDQ, while the NMGY exhibited relatively low genetic diversity.

**Table 1:** Genetic diversity level of ten subpopulations.

Population	Sample size	Observed heterozygosity (Ho)	Expected heterozygosity (He)	Nucleotide diversity index (Pi)
YLHG	5	0.204 22	0.152 39	0.169 32
TSBL	10	0.223 56	0.181 86	0.191 43
TCDQ	4	0.235 11	0.173 28	0.198 03
ARTS	3	0.230 11	0.159 74	0.191 69
KLSM	14	0.230 89	0.188 52	0.195 50
TSNL	10	0.214 18	0.174 53	0.183 72
THPD	5	0.216 48	0.162 17	0.180 19
QLSM	4	0.234 44	0.170 96	0.195 38
HTGY	6	0.231 97	0.178 76	0.195 01
NMGY	4	0.200 37	0.143 87	0.164 42

### 3.5 Population structure analysis and principal component analysis

**Figure 5:**
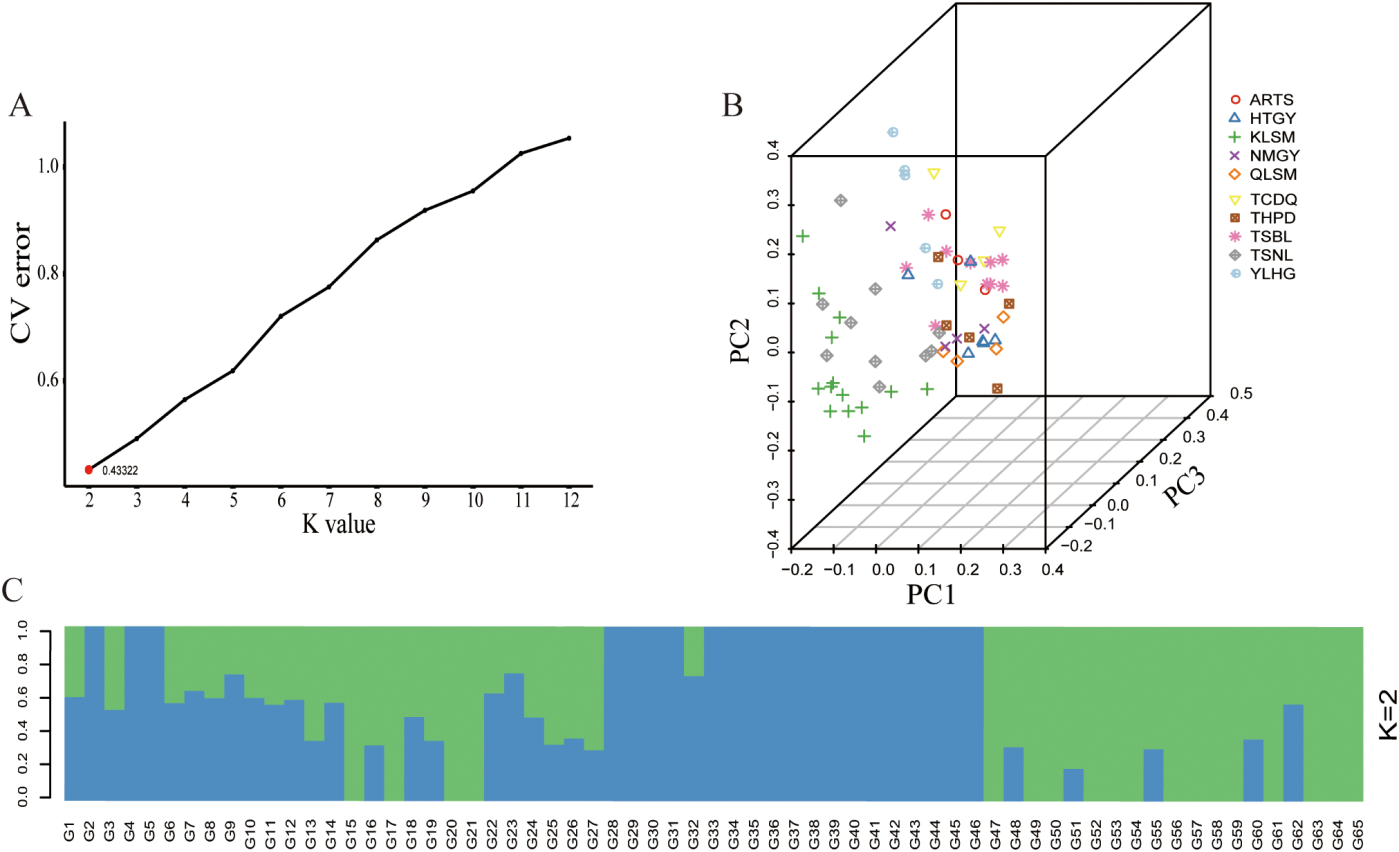
Population structure analysis and PCA of 65 samples of *S. alopecuroides*. **(A)** When K=2, the *CV* error value was the smallest (0.43322); **(B)** PCA of 65 *S. alopecuroides* samples; **(C)** Population structure separates the accessions into two subgroups (K=2).

To further understand the genetic background relationships of *S. alopecuroides* across different regions, Admixture software was used to analyze the population structure of 65 samples. The results showed that when K = 2, the CV error was the smallest, indicating that the optimal grouping of the genetic structure of the 65 *S. alopecuroides* samples consisted of two clusters (**Figure 5A, B**). Additionally, to supplement the results of the population structure analysis, we used GCTA for PCA. Based on the degree of SNP differences between individuals, PCA (**Figure 5C**) showed that the 65 *S. alopecuroides* samples could not be effectively divided into two groups, and the distribution of some populations gradually overlapped, which was consistent with the results of the population structure analysis.

### 3.6 Phylogenetic tree analysis

**Figure 6:**
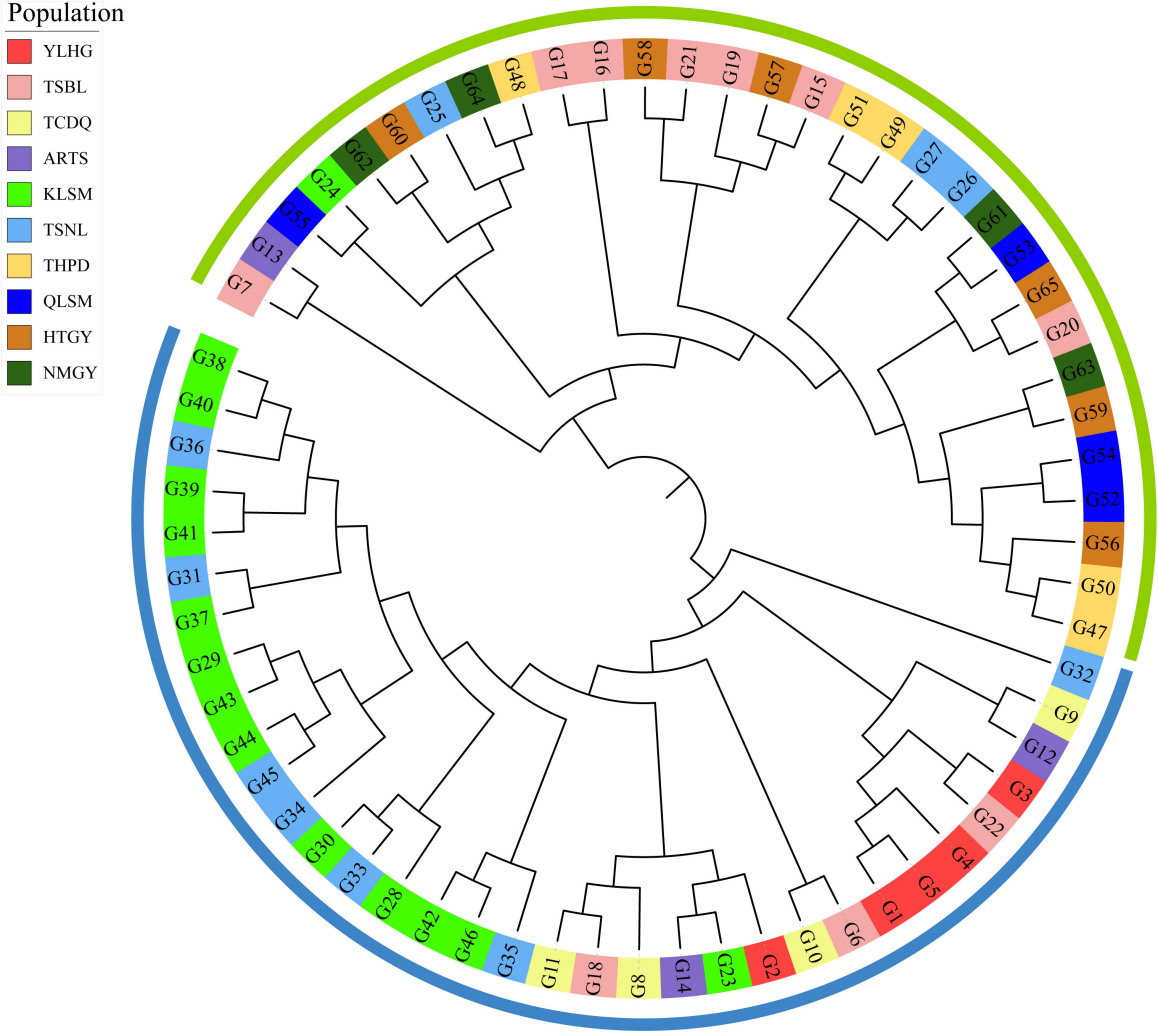
Phylogenetic analysis of 65 samples of *S. alopecuroides*. The outer color rings (green and blue) are equivalent to the structural analysis when K = 2. The background color of each sample label reflects its original population distribution.

The 10,584 filtered SNPs were used to analyze the phylogenetic tree of 65 samples from 10 *S. alopecuroides* populations using the neighbor-joining method (**Figure 6**). The results showed that the 65 samples could be divided into two large clusters. Generally, samples from the same geographical area showed a relative aggregation phenomenon in the two large clusters but did not merge into one group. Some samples were distributed across both clusters. Cluster I mainly included *S. alopecuroides* samples from the TSBL, QLSM, HTGY, NMGY, and THPD, while samples from the YLHG, TCDQ, and KLSM were clustered into cluster II. The distribution of *S. alopecuroides* samples in ARTS and TSNL was more dispersed across the two clusters. These findings indicate geographical isolation between the *S. alopecuroides* germplasm resources from different geographical regions.

### 3.7 Analysis of molecular variance and pairwise population differentiation

**Figure 7:**
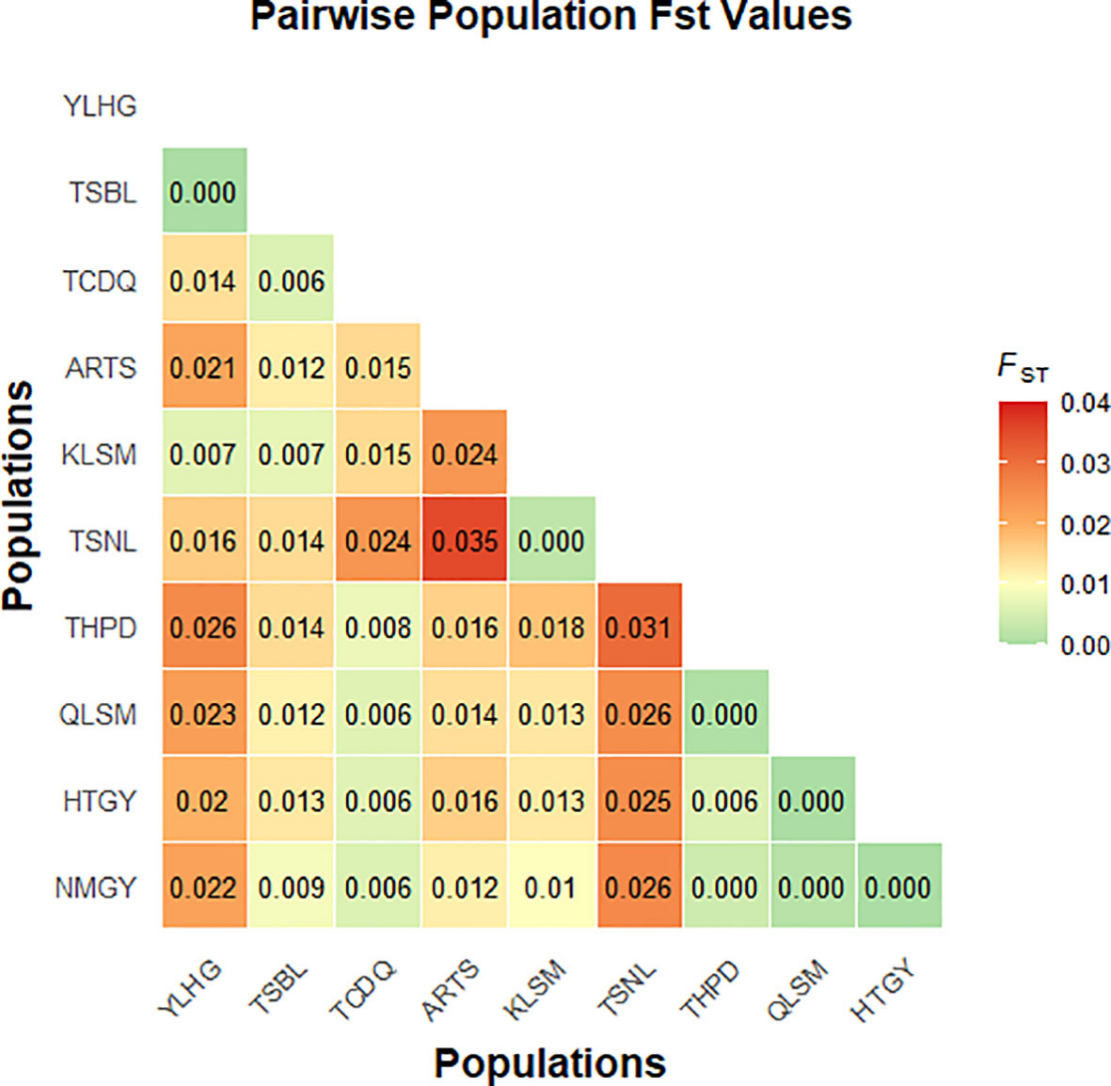
*F*_ST_ among 10 *S. alopecuroides* populations.

The *F*_ST_ based on SNP data ranged from 0.00 to 0.04 among the 10 populations (**Figure 7**). The highest *F*_ST_ value was observed between TSNL and ARTS, with relatively high values also found between YLHG, TSNL, and other populations. AMOVA results indicated that 1.94% of genetic variation was attributed to differences among populations, while 34.77% and 132.83% originated from among individuals and within individuals, respectively (**Table 2**). All differences were statistically significant (*P* < 0.05). These results suggest that most genetic variation in *S. alopecuroides* is derived from within-population differences. Notably, the occurrence of negative variance components may reflect limitations of the AMOVA model under small sample size and weak genetic structure conditions.

**Table 2:** Analysis of molecular variance analysis (AMOVA) of population.

Source of variation	df	Sum of squares	Variance components	Percentage of variation (%)
Among populations	9	879.598	2.16256Va	1.94
Among individuals	55	3876.463	-38.71712Vb	-34.77
Within individuals	65	9614.500	147.91538Vc	132.83
Total	129	14370.562	111.36083	100

### 3.8 Redundancy analysis

**Figure 8:**
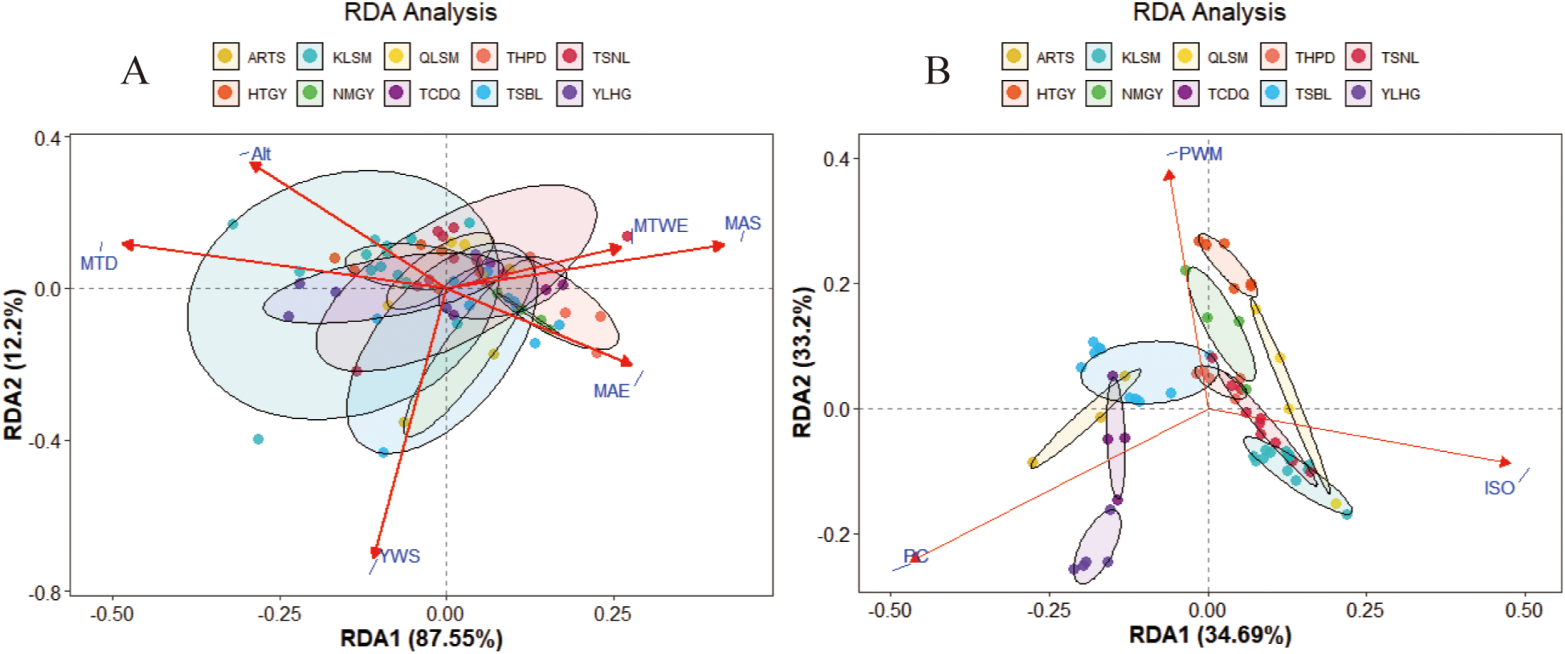
Redundancy analysis (RDA) of the relationship between seed phenotypic traits **(A)**, genetic diversity **(B)** and environmental factors. The red arrows represent environmental factors, and different color circles represent sample points and populations.

RDA revealed the relationship between phenotypic traits, genetic diversity, and environmental factors in *S. alopecuroides*. As shown in **Figures 8A, B**, the cumulative explanatory power of environmental factors for phenotypic traits and genetic diversity was 99.75% and 67.89%, respectively, suggesting that the first two RDA axes captured most of the variation. The length of the environmental vector indicates the strength of its influence, while the proximity between a sample point and an environmental vector reflects the degree of its impact. In **Figure 8A**, the key environmental factors influencing seed phenotypic traits included MTD (Mean Temperature of Driest Quarter), YWS (Annual Mean Wind Speed), Alt (Altitude), MAE (Mean Annual Evaporation), MTWE (Mean Temperature of Wettest Quarter), and MAS (Mean Annual Sunshine Time), with MTD, Alt, MAS, and YWS exerting the strongest effects. In **Figure 8B**, genetic diversity was primarily affected by PWE (Precipitation of Wettest Quarter), ISO (Isothermality), and PC (Precipitation of Coldest Quarter). Specifically, PC was most associated with YLHG, TCDQ, ARTS, and TSBL populations; ISO influenced KLSM, QLSM, and TSNL; and PWE had the greatest impact on HTGY, THPD, and NMGY populations.

## 4 Discussion

### 4.1 Genetic diversity

Genetic diversity analysis is crucial for the evaluation, utilization, origin, and evolution of plant germplasm resources (Lavanya et al., 2008; Huang L. et al., 2024). Phenotypic diversity comprehensively reflects genetic and environmental diversity (Nicotra et al., 2010). Through long-term natural selection, different populations may undergo significant genetic variation, leading to relatively stable phenotypic traits (Wu et al., 2018). In this study, the phenotypic traits of 65 *S. alopecuroides* seeds from various provenances were analyzed (**Supplementary Table S4**), revealing substantial phenotypic diversity. Among these, seed length, area, thousand-grain weight, and other traits exhibited greater variation, consistent with the findings of Yang et al. (Yang et al., 2010) and Wang et al. (Wang et al., 2019b). Additionally, H ranged from 1.639 to 1.767, and all traits followed a normal distribution (**Figure 2**), further verifying that phenotypic trait diversity was substantial and statistically uniform. Seed phenotypic traits are often interlinked, making correlation analysis crucial in the study of seed phenotypic diversity (Lu et al., 2010; Xu et al., 2024). The correlation analysis of phenotypic traits in *S. alopecuroides* seeds revealed significant correlations between most traits (*P* < 0.01) (**Figure 3**), suggesting a synergistic pattern in phenotypic expression, providing a foundation for further exploration of the genetic mechanisms underlying these traits.

The use of SNP molecular markers has become an important tool for identifying and conserving plant germplasm resources (Sun et al., 2011; Bendhifi et al., 2013). In this study, the genetic diversity analysis based on SNP markers showed that the genetic diversity of *S. alopecuroides* was low, with Ho, He, and Pi values of 0.22, 0.17, and 0.19 (**Table 1**), respectively, consistent with previous genetic diversity analyses of *S. alopecuroides* (Liu et al., 2013; Wang et al., 2019a; Li et al., 2020). Furthermore, it exhibited relatively low genetic diversity compared to other *Sophora* species (Heenan et al., 2018; Shu et al., 2019). Additionally, most species of the *Sophora* genus are monoecious, insect-pollinated, and self-compatible (Wang et al., 2019b); *S. alopecuroides* is no exception. These factors contribute to a low level of genetic diversity.

The AMOVA based on SNP markers showed that genetic variation among individuals was -34.77%, while the genetic variation within individuals was as high as 132.83% (**Table 2**). This indicates a high genetic similarity within the population, resulting in a negative difference between individuals. Meanwhile, the FST ranged from 0.00 to 0.04 (**Figure 7**), also lower than that of other *Sophora* species (Liu et al., 2006; Fu et al., 2024). Additionally, small differences between populations and low genetic differentiation suggest that these samples may belong to the same ancestral group, which subsequently experienced geographical isolation. The occurrence of negative genetic variation may reflect the limitations of AMOVA in poorly differentiated populations. Additionally, population size plays a critical role, as smaller populations reduce the evolutionary potential of wild species, thereby diminishing genetic diversity (Mondini et al., 2009; Frankham, 2010). Therefore, the small number of samples collected in this study may have led to aberrant results. Moreover, due to the small population sizes of *S. alopecuroides* studied previously and the limited sample sizes, understanding of the genetic structure and variation of *S. alopecuroides* remains constrained (Wang et al., 2019a; Li et al., 2020). In contrast, we collected a larger number of germplasm resources from 65 regions across five provinces in northwest China. Although the limitations of previous studies have been partially overcome, the results still indicate that fully revealing the genetic diversity and population structure of *S. alopecuroides* populations remains challenging.

### 4.2 Population structure

The population structure analysis in this study indicated that K = 2 is the optimal number of groups (**Figure 5**). PCA and phylogenetic tree analysis further confirmed the division of the populations into two main clusters (**Figure 6**). However, some populations were still divided into different clusters, likely due to the varying calculation focuses of the three analysis methods and the insufficient sample size, leading to populations with complex genetic relationships being split into different clusters. Geographical isolation also significantly impacts the genetic structure of populations, playing a major role in the formation of exotic species (Hoskin et al., 2005; Yang et al., 2017). The distribution of many *S. alopecuroides* populations across mountains and plateaus limits pollen and seed dispersal, creating barriers between populations (Ye et al., 2021). Through the above analysis, we found that according to the characteristics of geographical, ecological and climatic conditions, *S. alopecuroides* can be divided into two clusters: north and south, with the Tianshan Mountains and the Qilian Mountains as the boundaries. This north-south geographical division is consistent with the population structure classification of the sample. However, the TSNL and ARTS populations exhibited more complex patterns, with higher genetic differentiation coefficients than other populations. Simultaneously, due to human activities, the suitable habitat area of *S. alopecuroides* has been greatly reduced, resulting in a fragmented pattern (Rong et al., 2024). We speculate that the two populations originated from a common ancestral population and subsequently experienced geographical isolation. Human factors also exacerbated the isolation effect, and TSBL may serve as a transitional group between the two populations. The results of the phylogenetic tree analysis also support the possibility of this scenario. Similar patterns of genetic structure have been observed in other threatened or geographically restricted species. For example, *Scutellaria yildirimlii*, an endemic plant species in Turkey, exhibits moderate genetic diversity within populations but shows substantial genetic differentiation among populations due to limited gene flow (Yildirim et al., 2023). Similarly, the medicinal plant *Polygonatum cyrtonema* Hua exhibits low genetic differentiation, likely due to environmental heterogeneity and habitat fragmentation (Liu et al., 2023). These findings are consistent with the patterns observed in *S. alopecuroides* in this study, suggesting that even widely distributed species can exhibit relatively low inter-population genetic diversity due to the combined effects of complex ecological landscapes and anthropogenic pressures. Therefore, to safeguard the genetic diversity and long-term viability of *S. alopecuroides*, greater emphasis should be placed on conserving marginal and transitional populations. Future efforts should also prioritize monitoring and evaluating the genetic structure of populations across diverse habitats.

### 4.3 Bioclimatic redundancy analysis

Environmental factors are the primary limiting factors influencing the distribution and growth of plants on a large geographical scale. Differences in temperature, precipitation, and other factors across regions lead to significant differentiation in the growth adaptability of various populations to their habitats (Yuan et al., 2021; Ji et al., 2022). To further assess the impact of environmental factors on genetic variation, we performed RDA. The phenotypic traits are the most direct indicators of the impact of environmental factors on plants. We found that environmental factors influence the phenotype of *S. alopecuroides* seeds, particularly through external conditions such as average temperature during dry and wet seasons, average annual wind speed, evaporation, altitude, and sunlight. This is also a key factor influencing the distribution of *S. alopecuroides* (Wang, 2023; Yao et al., 2023). Rong et al. (Rong et al., 2024) also indicated that the future expansion of the *S. alopecuroides* population would be influenced by environmental factors, with temperature and rainfall being the primary factors. The molecular-level RDA shows that PC, ISO, and PWE are key factors. PC had a strong influence on the genetic diversity of *S. alopecuroides* populations (**Figure 8**). This reflects that seasonal variation in precipitation plays a key role in gene flow and adaptability within *S. alopecuroides* populations. ISO also significantly affects the genetic diversity of some populations, particularly in the KLSM, QLSM, and TSNL populations. In areas with high ISO, relatively gentle climate change promotes the stable growth of plants over time, facilitating gene flow and the accumulation of adaptive genetics (Bansal et al., 2015). This is the primary factor influencing the low genetic differentiation of populations in these areas.

## 5 Conclusion

This study utilized 10,584 SNP markers obtained via GBS technology, combined with seed phenotypic traits, to analyze the genetic diversity of 65 *S. alopecuroides* samples from 10 populations in northwest China. The results showed that the genetic diversity of *S. alopecuroides* populations was low, with no obvious differentiation. Genetic variation mainly originated from individuals. The population structure is divided into two main clusters, related to environmental factors such as geographical distribution and climatic conditions. These findings underscore the need for further genetic protection and breeding efforts, considering the geographical and genetic distances between populations. Therefore, based on the study of genetic characteristics of *S. alopecuroides* populations, *in situ* conservation and regeneration strategies should be developed, prioritizing populations with relatively high genetic diversity and strengthening introduction measures to maximize genetic diversity in *S. alopecuroides* populations. Future research should increase the sample size, combining population dynamics analysis, screening for adaptive genes, and examining the interaction between environment and genetics. This study provides a foundation for the genetic evaluation and conservation of *S. alopecuroides* genetic resources in China and offers valuable insights for its breeding programs.

## 6 Equations

Equation 1:


(1) CV=(Standard deviation×Mean)×100%

Equation 2:


(2) H=−∑PilnPi

Equation 3:


(3) $${\text{X}}^{*}=\frac{x-\bar{x}}{\sigma }$$

## Data Availability

The raw sequencing data have been deposited at the National Center for Biotechnology Information database under BioProject PRJNA1225430. The dataset associated with this study is available under the accession numbers SRP564835, SRR32391254-SRR32391318.

## Supplementary material

Supplementary Table 1 Detailed geographic location of 65 *Sophora alopecuroides* samples.

Supplementary Table 2 Diversity of seed phenotypic traits of *S. alopecuroides* samples.

Supplementary Table 3 22 bioclimatic variables in the 10 populations of S. alopecuroides.

Supplementary Table 4 22 bioclimatic variables in the 10 populations of *S. alopecuroides*.

Supplementary Table 5 Statistics of sequencing data.

Supplementary Table 6 Statistics of sequencing depth and coverage.

Supplementary File S1 Photographs of 10 populations of *Sophora alopecuroides* seeds taken by Wanshen SC-G automatic seed analyzer.

Supplementary File S2 All SNP variation information of *Sophora alopecuroides*.
