# Supplementary material for: Genetic diversity and population structure of Kudouzi (Sophora alopecuroides) in Northwest China revealed by SNP markers and seed phenotypic traits
Source: Front Plant Sci. 2025 Sep 4;16:1634581. doi: 10.3389/fpls.2025.1634581 (PMC12443825; doi:10.3389/fpls.2025.1634581)
Supplement: Supplementary Table 1 — Detailed geographic location of 65 Sophora alopecuroides samples. [file Table1.docx]

Supplementary Material

**Supplementary Table 1.** Detailed geographic location of 65 *Sophora alopecuroides* samples

| Sample | Population | Longitude(E◦) | Latitude(N◦) | Location |
| --- | --- | --- | --- | --- |
| G1 | YLHG | 81°41'26″ | 43°44'56″ | China: Yili, Yingtamu |
| G2 | YLHG | 81°23'21″ | 43°53'53″ | China: Yili, Yining |
| G3 | YLHG | 81°03'48″ | 43°51'07″ | China: Yili, Chabuchar |
| G4 | YLHG | 80°57'09″ | 43°56'26″ | China: Yili, Keketdala |
| G5 | YLHG | 80°44'42″ | 44°09'48″ | China: Yili, Huocheng |
| G6 | TSBL | 82°45'26″ | 44°33'25″ | China: Jinghe, Bajiahu |
| G7 | TSBL | 82°36'51″ | 44°31'32″ | China: Jinghe, 83rd Regiment |
| G8 | TCDQ | 82°59'24″ | 46°11'54″ | China: Tacheng, Yumin |
| G9 | TCDQ | 83°51'31″ | 46°48'39″ | China: Tacheng, Emin, 167th Regiment |
| G10 | TCDQ | 83°47'38″ | 46°27'48″ | China: Tacheng, Emin, Hojirte |
| G11 | TCDQ | 82°58'57″ | 46°47'34″ | China: Tacheng City |
| G12 | ARTS | 87°49'30″ | 47°21'11″ | China: Altay, Beitun |
| G13 | ARTS | 87°29'45″ | 47°06'52″ | China: Altay, Fuhai |
| G14 | ARTS | 86°24'02″ | 48°16'32″ | China: Altay, Habahe |
| G15 | TSBL | 90°00'32″ | 43°36'23″ | China: Changji, Qitai |
| G16 | TSBL | 86°48'40″ | 44°31'44″ | China: Changji, Hutubi |
| G17 | TSBL | 86°23'39″ | 44°30'24″ | China: Changji, Manas |
| G18 | TSBL | 86°16'12″ | 44°46'21″ | China: Shihezi, 148th Regiment |
| G19 | TSBL | 86°00'00″ | 44°18'24″ | China: Shihezi |
| G20 | TSBL | 87°36'37″ | 44°00'19″ | China: Urumqi, Midong |
| G21 | TSBL | 88°17'50″ | 43°21'31″ | China: Urumqi, Daban |
| G22 | TSBL | 82°20'57″ | 44°48'38″ | China: Bole, Shuanghe |
| G23 | KLSM | 85°32'31″ | 38°07'52″ | China: Bazhou, Qiemo |
| G24 | KLSM | 88°09'58″ | 39°02'08″ | China: Bazhou, Ruoqiang |
| G25 | TSNL | 86°15'38″ | 41°23'50″ | China: Bazhou, Yili |
| G26 | TSNL | 86°38'02″ | 42°02'06″ | China: Bazhou, Bole |
| G27 | TSNL | 86°18'57″ | 42°01'26″ | China: Bazhou, Yanqi |
| G28 | KLSM | 78°20'16″ | 37°35'52″ | China: Hetian, Pishan |
| G29 | KLSM | 80°09'33″ | 37°03'36″ | China: Hetian, Lopnur |
| G30 | KLSM | 81°28'35″ | 36°29'02″ | China: Hetian, Yutian |
| G31 | TSNL | 80°20'10″ | 41°18'33″ | China: Aksu City |
| G32 | TSNL | 82°57'10″ | 41°44'48″ | China: Aksu, Kuche |
| G33 | TSNL | 82°19'28″ | 41°32'14″ | China: Aksu, Xihe |
| G34 | TSNL | 80°36'38″ | 41°15'43″ | China: Aksu, Wensu |
| G35 | TSNL | 81°13'15″ | 40°32'59″ | China: Alar |
| G36 | TSNL | 78°35'30″ | 39°50'59″ | China: Kashgar, Bachu |
| G37 | KLSM | 76°44'39″ | 39°22'28″ | China: Kashgar, Jiashi |
| G38 | KLSM | 76°39'03″ | 39°15'26″ | China: Kashgar, Yuepu Lake |
| G39 | KLSM | 75°53'09″ | 39°21'58″ | China: Kashgar, Shufu |
| G40 | KLSM | 76°05'05″ | 39°20'34″ | China: Kashgar, Shule |
| G41 | KLSM | 76°11'29″ | 38°54'06″ | China: Kashgar, Yengisar |
| G42 | KLSM | 77°20'54″ | 37°56'53″ | China: Kashgar, Yecheng |
| G43 | KLSM | 77°16'29″ | 38°21'17″ | China: Kashgar, Shache |
| G44 | KLSM | 77°15'17″ | 38°13'56″ | China: Kashgar, Zepu |
| G45 | TSNL | 79°00'59″ | 39°51'58″ | China: Tumushuk |
| G46 | KLSM | 76°05'55″ | 39°12'56″ | China: Kizilsu, Akto |
| G47 | THPD | 89°03'58″ | 42°48'55″ | China: Turpan, Gaochang, Grape Town |
| G48 | THPD | 89°09'26″ | 42°56'56″ | China: Turpan, Gaochang, Yar Town |
| G49 | THPD | 88°37'39″ | 42°48'32″ | China: Toksun |
| G50 | THPD | 92°51'49″ | 43°03'29″ | China: Hami, Yizhou |
| G51 | THPD | 94°41'14″ | 43°15'49″ | China: Hami, Yuwu County |
| G52 | QLSM | 103°09'07″ | 38°36'39″ | China: Gansu, Wuwei |
| G53 | QLSM | 102°55'04″ | 37°28'37″ | China: Gansu, Wuwei, Gulang County |
| G54 | QLSM | 94°06'57″ | 39°53'09″ | China: Gansu, Jiuquan, Dunhuang |
| G55 | QLSM | 101°06'25″ | 38°47'30″ | China: Gansu, Zhangye, Sandan County |
| G56 | HTGY | 106°04'45″ | 37°21'59″ | China: Ningxia, Wuzhong |
| G57 | HTGY | 106°32'23″ | 38°42'54″ | China: Ningxia, Shizuishan |
| G58 | HTGY | 105°41'09″ | 37°29'27″ | China: Ningxia, Zhongwei, Zhongning County |
| G59 | HTGY | 106°00'45″ | 35°52'54″ | China: Ningxia, Guyuan, Xiji County |
| G60 | HTGY | 106°15'03″ | 36°00'56″ | China: Ningxia, Guyuan, Yuanzhou District |
| G61 | NMGY | 107°21'50″ | 40°45'04″ | China: Inner Mongolia, Bayanzhuoer |
| G62 | NMGY | 101°02'13″ | 41°57'52″ | China: Inner Mongolia, Ejina Banner |
| G63 | NMGY | 105°43'12″ | 38°51'45″ | China: Inner Mongolia, Alashan Left Banner |
| G64 | NMGY | 108°55'16″ | 40°29'41″ | China: Inner Mongolia, Ordos |
| G65 | HTGY | 107°48'16″ | 37°30'42″ | China: Shaanxi, Yulin |

**Supplementary Table 2.** Bioclimatic variables used in this study, and the mean (± SD) values of 65 *S. alopecuroides* samples.

| Code | Name | Source | Mean ± SD |
| --- | --- | --- | --- |
| AMT | Annual Mean Temperature (℃) | http://www.worldclim.org/ | 10.57±2.7 |
| MDR | Mean Diurnal Range | http://www.worldclim.org/ | 13.43±1.38 |
| ISO | Isothermality (×100) | http://www.worldclim.org/ | 28.51±3.71 |
| TS | Temperature Seasonality | http://www.worldclim.org/ | 1245.24±159.65 |
| MTWM | Max Temperature of Warmest Month (℃) | http://www.worldclim.org/ | 33.2±3.36 |
| MTCM | Min Temperature of Coldest Month (℃) | http://www.worldclim.org/ | -14.29±3.06 |
| MTWE | Mean Temperature of Wettest Quarter (℃) | http://www.worldclim.org/ | 24.19±3.63 |
| MTD | Mean Temperature of Driest Quarter (℃) | http://www.worldclim.org/ | -3.39±9.03 |
| MTWA | Mean Temperature of Warmest Quarter (℃) | http://www.worldclim.org/ | 24.67±3.02 |
| MTC | Mean Temperature of Coldest Quarter (℃) | http://www.worldclim.org/ | -5.71±3.25 |
| AP | Annual Precipitation (mm) | http://www.worldclim.org/ | 176.45±132.19 |
| PWM | Precipitation of Wettest Month (mm) | http://www.worldclim.org/ | 36.06±33.24 |
| PDM | Precipitation of Driest Month (mm) | http://www.worldclim.org/ | 3.78±4.52 |
| PSCV | Precipitation Seasonality (%) | http://www.worldclim.org/ | 66.95±26.01 |
| PWE | Precipitation of Wettest Quarter (mm) | http://www.worldclim.org/ | 88.05±76.49 |
| PD | Precipitation of Driest Quarter (mm) | http://www.worldclim.org/ | 13.92±15.95 |
| PWA | Precipitation of Warmest Quarter (mm) | http://www.worldclim.org/ | 81.42±71.31 |
| PC | Precipitation of Coldest Quarter(mm) | http://www.worldclim.org/ | 14.91±16.64 |
| Alt | Altitude (m) | Local weather stations | 1020.6±504.18 |
| MAS | Mean annual sunshine time (h) | Local weather stations | 2853.6±380.5 |
| YWS | Annual Mean Wind Speed (m/s) | Local weather stations | 2.46±1.03 |
| MAE | Mean annual evaporation (mm) | Local weather stations | 2081.45±565.66 |

**Supplementary Table 3.** 22 bioclimatic variables in the 10 populations of *S. alopecuroides.*

| Bioclimatic variables | Population | | | | | | | | | |
| --- | --- | --- | --- | --- | --- | --- | --- | --- | --- | --- |
|  | YLHG | TSBL | TCDQ | ARTS | KLSM | TSNL | THPD | QLSM | HTGY | NMGY |
| AMT (℃) | 9.98 | 7.53 | 6.87 | 9.03 | 12.32 | 13.12 | 12.78 | 8.40 | 9.08 | 9.33 |
| MDR | 13.56 | 12.28 | 11.77 | 11.87 | 14.25 | 14.31 | 13.48 | 14.93 | 12.98 | 13.63 |
| ISO | 27.52 | 25.65 | 23.33 | 23.24 | 29.87 | 31.86 | 26.28 | 33.20 | 31.47 | 28.20 |
| TS | 1275.44 | 1330.93 | 1414.40 | 1446.52 | 1214.36 | 1139.34 | 1382.60 | 1084.55 | 1023.23 | 1261.50 |
| MTWM (℃) | 32.82 | 31.55 | 31.87 | 33.23 | 34.35 | 34.41 | 37.70 | 30.10 | 28.97 | 32.90 |
| MTCM (℃) | -16.52 | -16.40 | -18.53 | -18.10 | -13.31 | -10.48 | -13.86 | -14.85 | -12.27 | -15.45 |
| MTWE (℃) | 20.02 | 21.58 | 22.33 | 24.02 | 26.70 | 25.52 | 29.30 | 22.08 | 20.62 | 24.00 |
| MTD (℃) | 13.72 | -10.75 | -10.10 | -13.25 | -3.84 | 5.54 | -5.68 | -5.95 | -5.95 | -8.65 |
| MTWA (℃) | 24.02 | 22.95 | 23.17 | 25.15 | 25.72 | 25.76 | 28.72 | 21.20 | 21.22 | 24.25 |
| MTC (℃) | -7.24 | -9.28 | -11.20 | -10.15 | -3.76 | -2.08 | -5.04 | -5.48 | -3.82 | -6.53 |
| AP (mm) | 279.20 | 278.25 | 226.00 | 189.50 | 95.00 | 65.79 | 55.00 | 205.75 | 440.00 | 194.50 |
| PWM (mm) | 32.40 | 39.75 | 34.00 | 29.10 | 20.50 | 13.43 | 13.00 | 55.75 | 119.33 | 58.25 |
| PDM (mm) | 14.20 | 10.25 | 8.67 | 6.40 | 1.30 | 0.57 | 1.00 | 1.00 | 1.67 | 1.00 |
| PSCV% | 25.14 | 42.58 | 36.47 | 47.26 | 78.80 | 69.19 | 60.50 | 96.35 | 102.55 | 103.43 |
| PWE (mm) | 91.80 | 113.00 | 82.67 | 79.60 | 53.90 | 30.57 | 30.60 | 129.00 | 268.50 | 130.25 |
| PD (mm) | 53.40 | 35.00 | 32.00 | 21.10 | 4.50 | 3.50 | 3.80 | 3.50 | 7.50 | 4.75 |
| PWA (mm) | 74.60 | 95.50 | 80.67 | 69.60 | 53.80 | 29.50 | 30.40 | 127.25 | 249.00 | 123.25 |
| PC (mm) | 56.20 | 35.75 | 36.33 | 21.10 | 4.80 | 5.71 | 3.80 | 3.50 | 7.50 | 4.75 |
| Alt (m) | 601.40 | 816.75 | 573.33 | 620.80 | 1073.90 | 1302.64 | 505.80 | 1770.50 | 1474.83 | 1175.25 |
| MAS (h) | 2733.20 | 2797.95 | 2815.07 | 2615.90 | 3011.93 | 2834.51 | 3236.78 | 2882.23 | 2718.73 | 3048.58 |
| YWS (m/s) | 1.82 | 2.18 | 3.66 | 2.98 | 2.16 | 2.30 | 2.46 | 2.42 | 2.28 | 2.95 |
| MAE (mm) | 1780.04 | 1900.00 | 1761.67 | 1802.67 | 2048.78 | 2190.86 | 2659.26 | 2415.80 | 1661.77 | 2848.13 |

**Supplementary Table 4.** Diversity of seed phenotypic traits of *S. alopecuroides* samples.

| Parameter | Length  (mm) | Width  (mm) | Diameter  (mm) | Roundness | Area  (mm^2^) | Perimeter  (mm) | Shape index | Weight  (g) |
| --- | --- | --- | --- | --- | --- | --- | --- | --- |
| Maximum | 4.271 | 3.023 | 3.626 | 0.840 | 1.434 | 10.356 | 12.136 | 29.060 |
| Minimum | 3.461 | 2.641 | 3.074 | 0.700 | 1.200 | 7.464 | 10.129 | 20.056 |
| Mean | 3.728 | 2.838 | 3.302 | 0.764 | 1.317 | 8.607 | 10.878 | 23.776 |
| SD | 0.161 | 0.081 | 0.105 | 0.029 | 0.049 | 0.553 | 0.388 | 1.888 |
| CV | 4.32% | 2.87% | 3.19% | 3.80% | 6.43% | 3.56% | 3.69% | 7.94% |
| H | 1.654 | 1.767 | 1.725 | 1.736 | 1.721 | 1.639 | 1.737 | 1.724 |
| Levene’s p | 0.072 | 0.187 | 0.068 | 0.145 | 0.084 | 0.063 | 0.173 | 0.219 |
| K–S p | 0.525 | 0.275 | 0.815 | 0.07 | 0.065 | 0.667 | 0.942 | 0.053 |

SD: standard deviation; *CV*: coefficient of variation; *H*: Shannon 's diversity index; Shape: shape index; Weight: thousand-grain weight; K–S: Kolmogorov-Smirnov normality test.

**Supplementary Table 5.** Statistics of sequencing data**.**

| Parameter | Raw Base(bp) | Clean Base(bp) | Effective Rate (%) | Error Rate (%) | Q20(%) | Q30(%) | GC Content (%) |
| --- | --- | --- | --- | --- | --- | --- | --- |
| G1 | 846,332,064 | 833,247,072 | 98.45 | 0.03 | 96.73 | 90.63 | 37.37 |
| G2 | 757,958,112 | 697,625,856 | 92.04 | 0.03 | 96.74 | 90.66 | 38.41 |
| G3 | 732,387,168 | 699,700,032 | 95.54 | 0.03 | 96.52 | 90.11 | 38.77 |
| G4 | 855,351,360 | 841,714,848 | 98.41 | 0.03 | 96.97 | 91.22 | 37.88 |
| G5 | 864,516,672 | 840,940,992 | 97.27 | 0.03 | 97 | 91.28 | 37.75 |
| G6 | 975,371,904 | 941,950,080 | 96.57 | 0.03 | 96.81 | 90.77 | 38.23 |
| G7 | 791,121,024 | 756,074,016 | 95.57 | 0.03 | 96.08 | 89 | 38.64 |
| G8 | 862,517,088 | 798,541,344 | 92.58 | 0.03 | 97.56 | 92.67 | 39.78 |
| G9 | 682,431,264 | 638,822,304 | 93.61 | 0.04 | 95.52 | 88.5 | 38.23 |
| G10 | 877,143,168 | 823,196,448 | 93.85 | 0.03 | 97.21 | 91.83 | 38.69 |
| G11 | 872,641,728 | 808,740,000 | 92.68 | 0.03 | 97.11 | 91.53 | 38.6 |
| G12 | 1,017,356,544 | 982,456,704 | 96.57 | 0.03 | 96.72 | 90.44 | 37.95 |
| G13 | 674,231,616 | 604,058,400 | 89.59 | 0.03 | 96.35 | 89.69 | 37.5 |
| G14 | 580,247,712 | 493,283,808 | 85.01 | 0.03 | 96.35 | 89.62 | 38.45 |
| G15 | 564,987,744 | 500,395,680 | 88.57 | 0.03 | 96.19 | 89.28 | 38.37 |
| G16 | 594,109,152 | 516,402,144 | 86.92 | 0.03 | 96.78 | 90.77 | 38.32 |
| G17 | 571,857,696 | 480,835,584 | 84.08 | 0.03 | 97.29 | 92.11 | 37.7 |
| G18 | 533,957,184 | 488,007,072 | 91.39 | 0.03 | 96.88 | 91.05 | 38.33 |
| G19 | 663,359,328 | 594,378,720 | 89.60 | 0.03 | 96.86 | 90.99 | 37.52 |
| G20 | 544,020,768 | 503,392,320 | 92.53 | 0.03 | 97.48 | 92.58 | 38.41 |
| G21 | 599,568,768 | 515,522,592 | 85.98 | 0.03 | 97.1 | 91.59 | 37.36 |
| G22 | 574,043,328 | 547,703,424 | 95.41 | 0.03 | 95.93 | 88.71 | 37.87 |
| G23 | 715,893,408 | 647,638,560 | 90.47 | 0.03 | 96.23 | 89.5 | 38.47 |
| G24 | 607,448,448 | 551,792,160 | 90.84 | 0.03 | 96.62 | 90.44 | 38.52 |
| G25 | 1,108,956,096 | 1,054,205,856 | 95.06 | 0.03 | 95.59 | 88.15 | 38.09 |
| G26 | 677,219,616 | 643,507,200 | 95.02 | 0.03 | 97.51 | 92.69 | 38.38 |
| G27 | 547,154,208 | 503,897,184 | 92.09 | 0.03 | 97.73 | 93.25 | 38.52 |
| G28 | 735,442,272 | 686,697,984 | 93.37 | 0.03 | 96.61 | 90.44 | 38.41 |
| G29 | 634,609,440 | 589,648,896 | 92.92 | 0.03 | 96.91 | 91.2 | 38.88 |
| G30 | 795,091,680 | 748,764,288 | 94.17 | 0.03 | 96.05 | 89.16 | 38.4 |
| G31 | 1,134,652,896 | 1,089,161,856 | 95.99 | 0.03 | 95.59 | 88.14 | 37.89 |
| G32 | 936,058,176 | 900,916,128 | 96.25 | 0.03 | 96.91 | 91.05 | 37.53 |
| G33 | 839,331,648 | 779,916,384 | 92.92 | 0.03 | 96.08 | 89.18 | 38.32 |
| G34 | 789,131,232 | 764,948,160 | 96.94 | 0.04 | 93.97 | 84.85 | 38.21 |
| G35 | 997,536,960 | 939,475,872 | 94.18 | 0.03 | 97.03 | 91.37 | 37.75 |
| G36 | 906,260,832 | 897,804,864 | 99.07 | 0.03 | 95.83 | 87.99 | 35.61 |
| G37 | 1,327,752,000 | 1,287,069,984 | 96.94 | 0.03 | 96.84 | 90.93 | 36.97 |
| G38 | 916,627,680 | 851,369,472 | 92.88 | 0.03 | 96.7 | 90.67 | 38.17 |
| G39 | 1,061,084,736 | 989,568,864 | 93.26 | 0.03 | 96.44 | 90.04 | 38.12 |
| G40 | 1,591,091,424 | 1,556,618,112 | 97.83 | 0.03 | 97.12 | 91.59 | 37.42 |
| G41 | 1,136,632,608 | 1,089,145,728 | 95.82 | 0.03 | 97.09 | 91.53 | 37.94 |
| G42 | 1,004,207,328 | 884,054,880 | 88.04 | 0.03 | 96.83 | 90.95 | 37.89 |
| G43 | 939,291,264 | 887,690,592 | 94.51 | 0.03 | 95.98 | 88.93 | 38.5 |
| G44 | 897,800,256 | 855,999,648 | 95.34 | 0.03 | 97.62 | 92.87 | 38.7 |
| G45 | 1,014,556,608 | 977,730,624 | 96.37 | 0.03 | 96.95 | 91.23 | 38.17 |
| G46 | 972,296,928 | 921,687,552 | 94.79 | 0.03 | 97.34 | 92.21 | 38.53 |
| G47 | 795,584,448 | 711,016,416 | 89.37 | 0.03 | 97.05 | 91.45 | 36.98 |
| G48 | 1,093,358,880 | 1,051,081,056 | 96.13 | 0.03 | 96.66 | 90.38 | 37.39 |
| G49 | 695,937,312 | 669,825,216 | 96.25 | 0.03 | 95.52 | 88.02 | 37.42 |
| G50 | 629,607,744 | 577,120,032 | 91.66 | 0.04 | 95.29 | 87.42 | 38.59 |
| G51 | 919,853,280 | 860,416,704 | 93.54 | 0.03 | 95.77 | 88.31 | 38.72 |
| G52 | 845,119,008 | 789,177,600 | 93.38 | 0.03 | 96.03 | 89.19 | 37.84 |
| G53 | 747,448,704 | 690,505,920 | 92.38 | 0.03 | 96.74 | 91.02 | 38.33 |
| G54 | 810,942,912 | 746,272,800 | 92.03 | 0.03 | 96.37 | 89.97 | 37.45 |
| G55 | 919,064,736 | 858,089,952 | 93.37 | 0.03 | 96.23 | 89.72 | 37.12 |
| G56 | 748,770,912 | 683,078,112 | 91.23 | 0.03 | 97.06 | 91.75 | 37.86 |
| G57 | 637,131,744 | 565,456,320 | 88.75 | 0.03 | 96.62 | 90.6 | 38.52 |
| G58 | 837,766,368 | 786,948,480 | 93.93 | 0.04 | 95.09 | 87.04 | 37.88 |
| G59 | 611,091,648 | 550,270,656 | 90.05 | 0.03 | 95.59 | 88.21 | 38.67 |
| G60 | 1,169,224,416 | 1,145,130,912 | 97.94 | 0.03 | 97.69 | 92.68 | 35.98 |
| G61 | 739,202,112 | 693,167,040 | 93.77 | 0.03 | 95.22 | 87.23 | 38.49 |
| G62 | 1,131,595,200 | 1,092,912,192 | 96.58 | 0.04 | 95.00 | 87.46 | 38.19 |
| G63 | 578,210,976 | 541,644,480 | 93.68 | 0.03 | 97.14 | 91.99 | 38.36 |
| G64 | 748,494,144 | 715,066,848 | 95.53 | 0.03 | 96.32 | 89.66 | 38.52 |
| G65 | 787,123,872 | 696,104,352 | 88.44 | 0.03 | 96.62 | 90.47 | 38.16 |
| Maximum | 1,591,091,424 | 1,556,618,112 | 99.07 | 0.04 | 97.73 | 93.25 | 39.78 |
| Minimum | 533,957,184 | 480,835,584 | 84.08 | 0.03 | 93.97 | 84.85 | 35.61 |
| Mean | 827,187,193 | 775,839,775 | 93.31 | 0.03 | 96.51 | 90.24 | 38.06 |
| Total | 53,767,167,552 | 50,429,585,376 |  |  |  |  |  |

**Supplementary Table 6.** Statistics of sequencing depth and coverage.

| Parameter | Clean Reads | Mapped Reads | Mapping Rate (%) | Average Depth | Coverage at Least 1X (%) | Coverage at Least 4X (%) |
| --- | --- | --- | --- | --- | --- | --- |
| G1 | 5,786,438 | 5,261,858 | 90.93% | 20.57 | 78.06% | 56.09% |
| G2 | 4,844,624 | 4,122,612 | 85.10% | 17.18 | 75.21% | 51.77% |
| G3 | 4,859,028 | 4,024,741 | 82.83% | 17.46 | 70.37% | 50.19% |
| G4 | 5,845,242 | 5,334,694 | 91.27% | 21.26 | 74.29% | 54.82% |
| G5 | 5,839,868 | 5,236,192 | 89.66% | 20.34 | 77.03% | 56.33% |
| G6 | 6,541,320 | 5,705,560 | 87.22% | 21.51 | 78.69% | 57.58% |
| G7 | 5,250,514 | 4,593,894 | 87.49% | 18.91 | 74.33% | 53.11% |
| G8 | 5,545,426 | 4,745,924 | 85.58% | 20.77 | 72.43% | 48.69% |
| G9 | 4,436,266 | 3,680,767 | 82.97% | 16.18 | 76.38% | 47.63% |
| G10 | 5,716,642 | 4,919,139 | 86.05% | 20.05 | 74.21% | 53.41% |
| G11 | 5,616,250 | 4,738,807 | 84.38% | 18.65 | 79.15% | 54.77% |
| G12 | 6,822,616 | 5,982,027 | 87.68% | 22.09 | 81.34% | 58.61% |
| G13 | 4,194,850 | 3,439,527 | 81.99% | 15.01 | 77.46% | 47.75% |
| G14 | 3,425,582 | 2,801,484 | 81.78% | 13.44 | 72.12% | 43.12% |
| G15 | 3,474,970 | 2,902,271 | 83.52% | 13.68 | 72.08% | 44.25% |
| G16 | 3,586,126 | 3,015,223 | 84.08% | 13.89 | 72.85% | 45.32% |
| G17 | 3,339,136 | 2,709,853 | 81.15% | 12.87 | 72.69% | 43.32% |
| G18 | 3,388,938 | 2,951,161 | 87.08% | 14.2 | 68.26% | 43.96% |
| G19 | 4,127,630 | 3,474,161 | 84.17% | 14.89 | 75.46% | 49.61% |
| G20 | 3,495,780 | 3,074,348 | 87.94% | 14.21 | 71.30% | 45.63% |
| G21 | 3,580,018 | 2,913,924 | 81.39% | 13.2 | 73.66% | 45.98% |
| G22 | 3,803,496 | 3,360,775 | 88.36% | 15.15 | 72.32% | 47.23% |
| G23 | 4,497,490 | 3,826,710 | 85.09% | 16.55 | 73.06% | 49.41% |
| G24 | 3,831,890 | 3,309,637 | 86.37% | 14.93 | 72.02% | 47.17% |
| G25 | 7,320,874 | 6,535,782 | 89.28% | 24.8 | 77.64% | 57.11% |
| G26 | 4,468,800 | 4,051,266 | 90.66% | 18.48 | 68.86% | 47.81% |
| G27 | 3,499,286 | 3,115,114 | 89.02% | 15.64 | 66.40% | 42.53% |
| G28 | 4,768,736 | 4,244,622 | 89.01% | 19.26 | 68.22% | 47.93% |
| G29 | 4,094,784 | 3,673,099 | 89.70% | 18.25 | 64.73% | 43.51% |
| G30 | 5,199,752 | 4,677,226 | 89.95% | 20.89 | 68.26% | 48.92% |
| G31 | 7,563,624 | 6,802,626 | 89.94% | 26.35 | 75.52% | 55.67% |
| G32 | 6,256,362 | 5,618,321 | 89.80% | 22.56 | 75.09% | 53.71% |
| G33 | 5,416,086 | 4,781,550 | 88.28% | 20.71 | 71.12% | 49.77% |
| G34 | 5,312,140 | 4,795,244 | 90.27% | 21.02 | 70.10% | 49.53% |
| G35 | 6,524,138 | 5,760,130 | 88.29% | 23.15 | 74.63% | 53.33% |
| G36 | 6,234,756 | 5,628,353 | 90.27% | 19 | 82.35% | 62.56% |
| G37 | 8,937,986 | 7,957,377 | 89.03% | 28.34 | 80.40% | 59.15% |
| G38 | 5,912,288 | 5,214,978 | 88.21% | 21.9 | 72.58% | 51.83% |
| G39 | 6,872,006 | 6,067,970 | 88.30% | 24.26 | 74.48% | 54.09% |
| G40 | 10,809,848 | 9,812,988 | 90.78% | 34.92 | 78.76% | 60.30% |
| G41 | 7,563,512 | 6,778,767 | 89.62% | 26.66 | 74.35% | 54.63% |
| G42 | 6,139,270 | 5,124,599 | 83.47% | 20.44 | 77.47% | 54.02% |
| G43 | 6,164,518 | 5,523,499 | 89.60% | 23.24 | 71.69% | 52.02% |
| G44 | 5,944,442 | 5,385,795 | 90.60% | 23.72 | 68.27% | 49.48% |
| G45 | 6,789,796 | 6,171,034 | 90.89% | 24.86 | 73.26% | 54.05% |
| G46 | 6,400,608 | 5,732,272 | 89.56% | 24.18 | 71.54% | 51.61% |
| G47 | 4,937,614 | 4,161,731 | 84.29% | 17.26 | 76.90% | 51.19% |
| G48 | 7,299,174 | 6,422,072 | 87.98% | 24.12 | 78.77% | 56.71% |
| G49 | 4,651,564 | 4,199,364 | 90.28% | 18.85 | 71.56% | 47.46% |
| G50 | 4,007,778 | 3,514,533 | 87.69% | 17.29 | 66.62% | 43.85% |
| G51 | 5,975,116 | 5,280,762 | 88.38% | 22.76 | 72.86% | 50.39% |
| G52 | 5,480,400 | 4,792,407 | 87.45% | 19.67 | 77.14% | 52.70% |
| G53 | 4,795,180 | 4,211,048 | 87.82% | 18.34 | 74.08% | 49.57% |
| G54 | 5,182,450 | 4,486,312 | 86.57% | 19.38 | 73.54% | 49.97% |
| G55 | 5,958,958 | 5,149,727 | 86.42% | 20.51 | 78.62% | 53.63% |
| G56 | 4,743,598 | 4,077,745 | 85.96% | 17.66 | 75.34% | 49.36% |
| G57 | 3,926,780 | 3,337,716 | 85.00% | 16.2 | 70.83% | 43.63% |
| G58 | 5,464,920 | 4,822,238 | 88.24% | 19.78 | 77.24% | 52.83% |
| G59 | 3,821,324 | 3,281,304 | 85.87% | 15.93 | 70.06% | 43.81% |
| G60 | 7,952,298 | 7,512,185 | 94.47% | 18.5 | 99.90% | 87.62% |
| G61 | 4,813,660 | 4,281,247 | 88.94% | 19.32 | 70.78% | 48.26% |
| G62 | 7,589,668 | 6,584,865 | 86.76% | 22.21 | 84.10% | 64.96% |
| G63 | 3,761,420 | 3,348,472 | 89.02% | 16.26 | 68.01% | 44.27% |
| G64 | 4,965,742 | 4,466,827 | 89.95% | 20.28 | 68.80% | 47.50% |
| G65 | 4,834,058 | 4,090,609 | 84.62% | 17.73 | 76.22% | 49.35% |
| Maximum | 10,809,848 | 9,812,988 | 94.47% | 34.92 | 0.10% | 12.38% |
| Minimum | 3,339,136 | 2,709,853 | 81.15% | 12.87 | 35.27% | 57.47% |
| Mean | 5,387,776 | 4,732,293 | 87.45% | 19.56 | 25.82% | 48.67% |
