# Supplementary figures and images for: Genetic diversity and population structure of Kudouzi (Sophora alopecuroides) in Northwest China revealed by SNP markers and seed phenotypic traits
Source: Front Plant Sci. 2025 Sep 4;16:1634581. doi: 10.3389/fpls.2025.1634581 (PMC12443825; doi:10.3389/fpls.2025.1634581)

G12

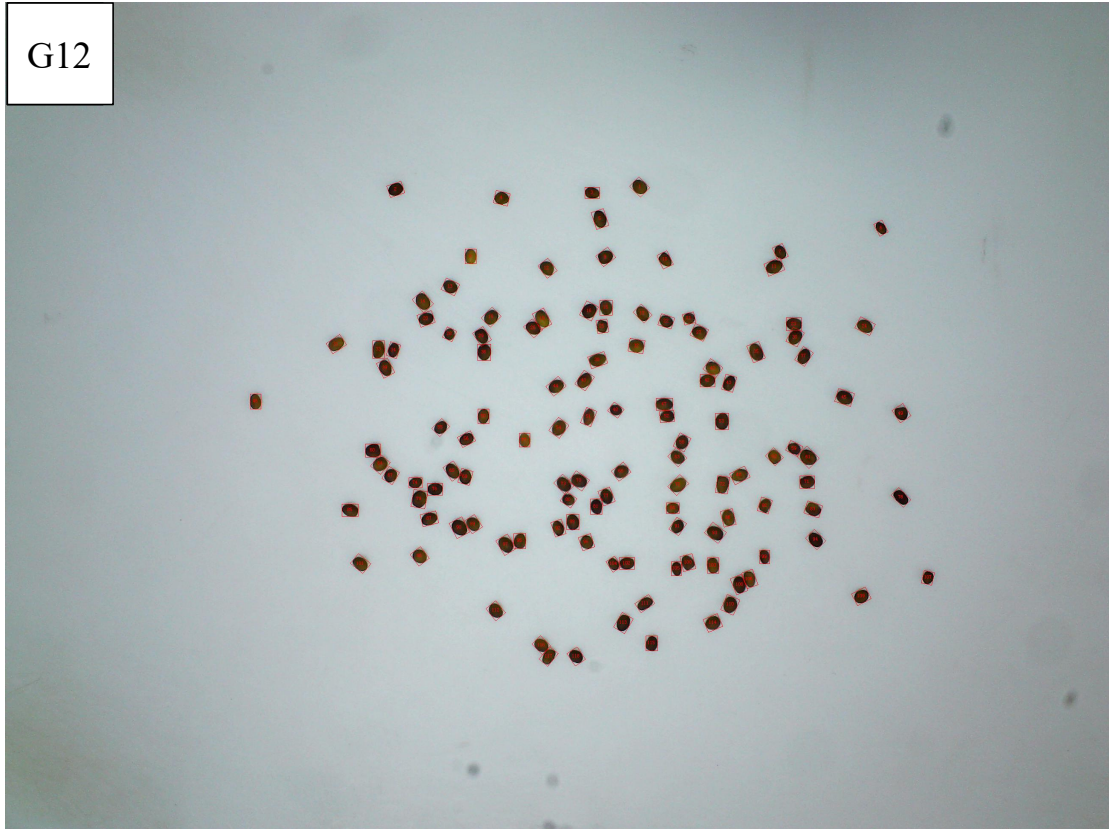

G13

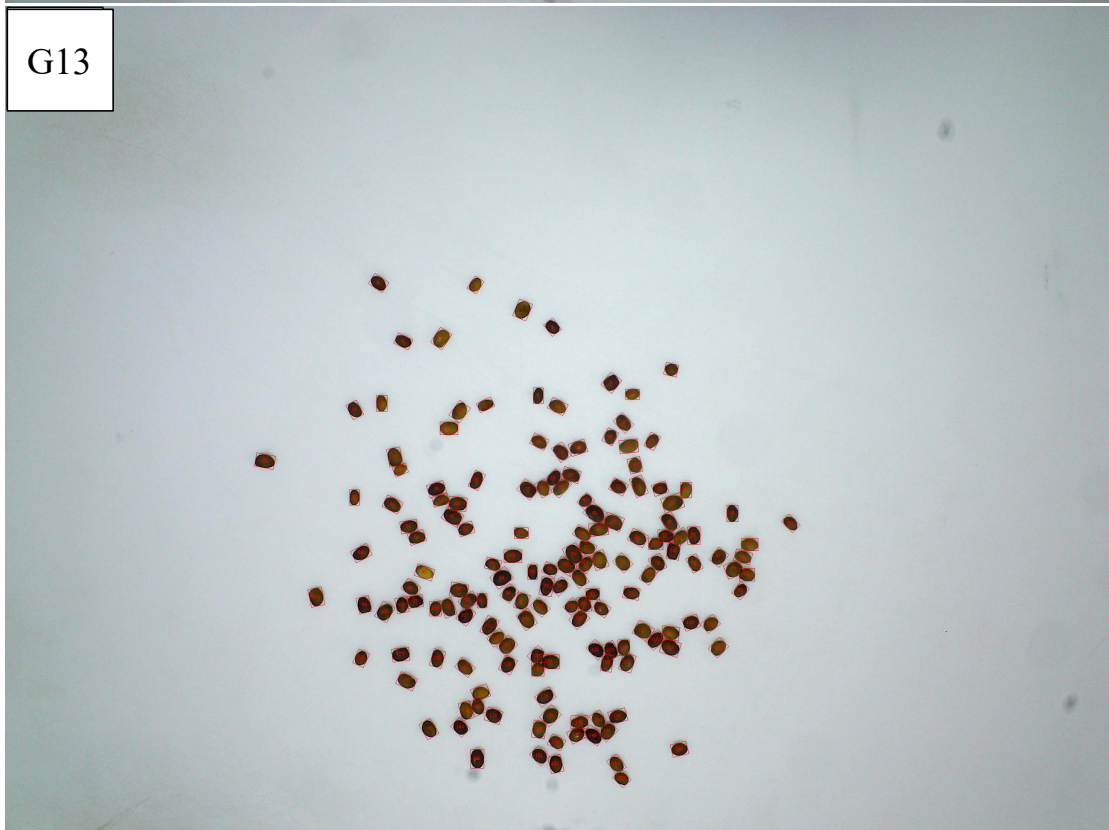

G14

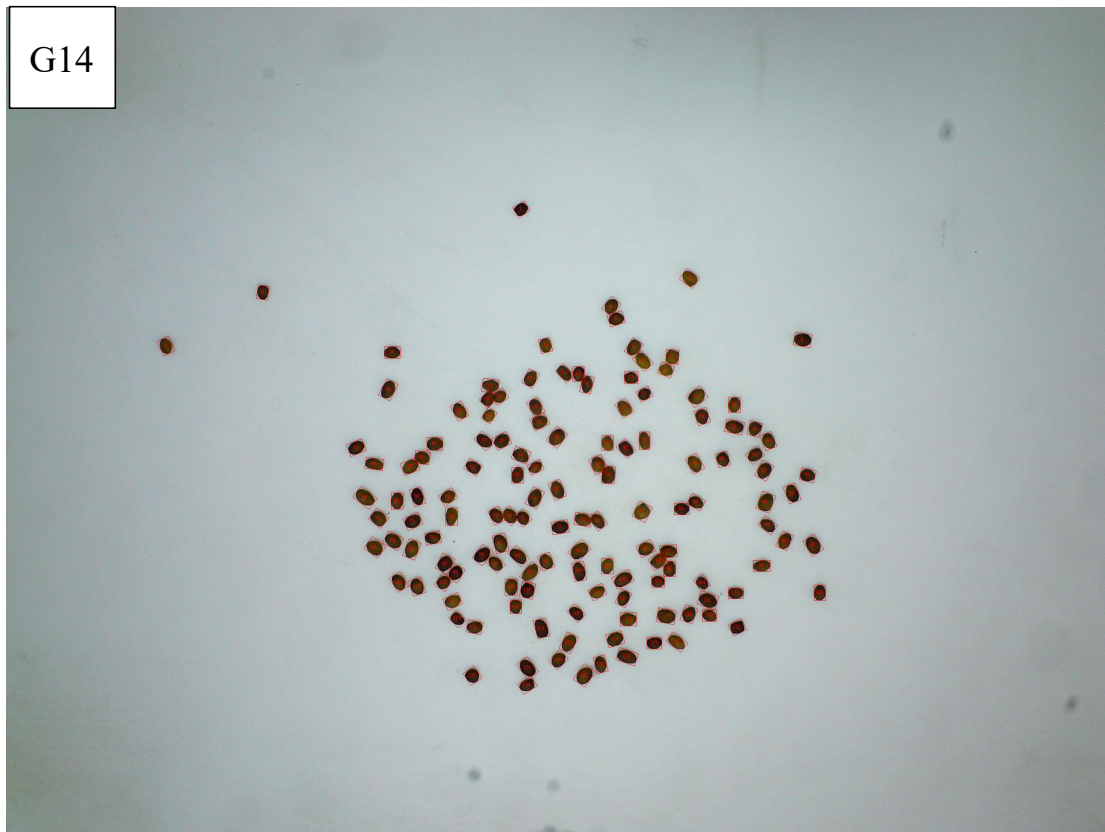

Supplement: Supplementary Table 3 — 22 bioclimatic variables in the 10 populations of S. alopecuroides. [file DataSheet2.zip › Supplementary file 1/ARTS.pdf]

G56

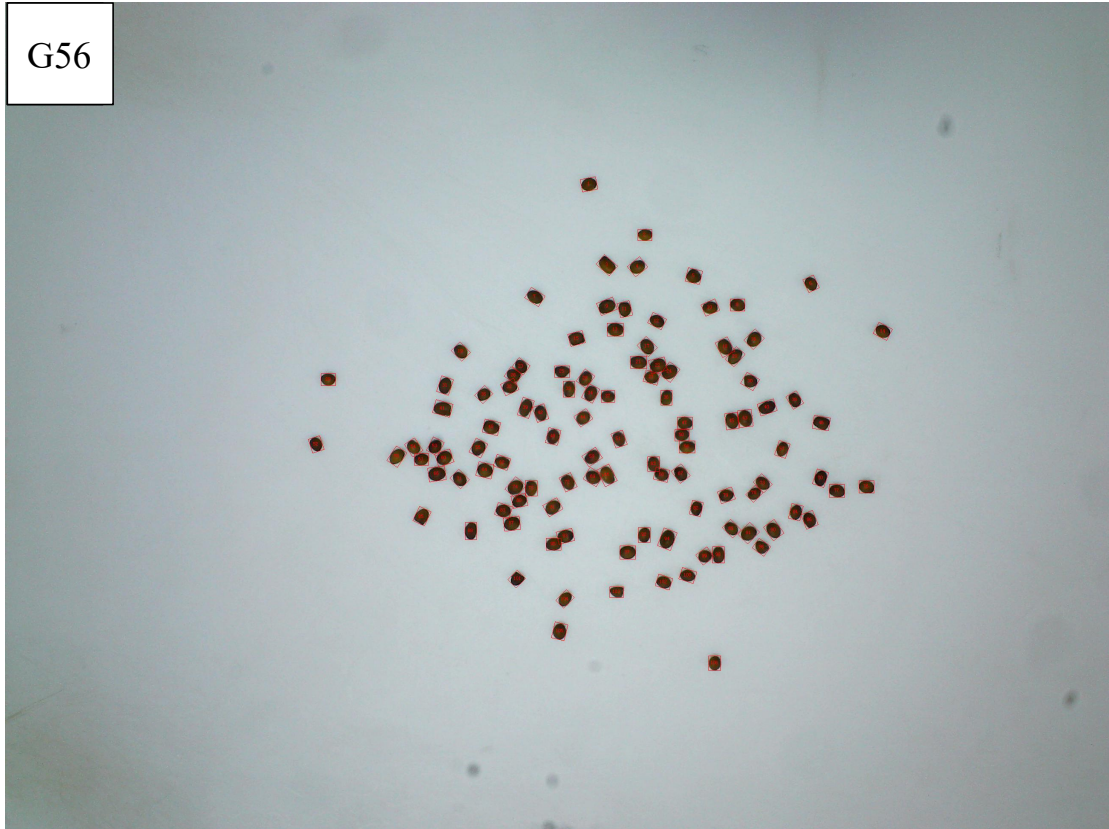

G57

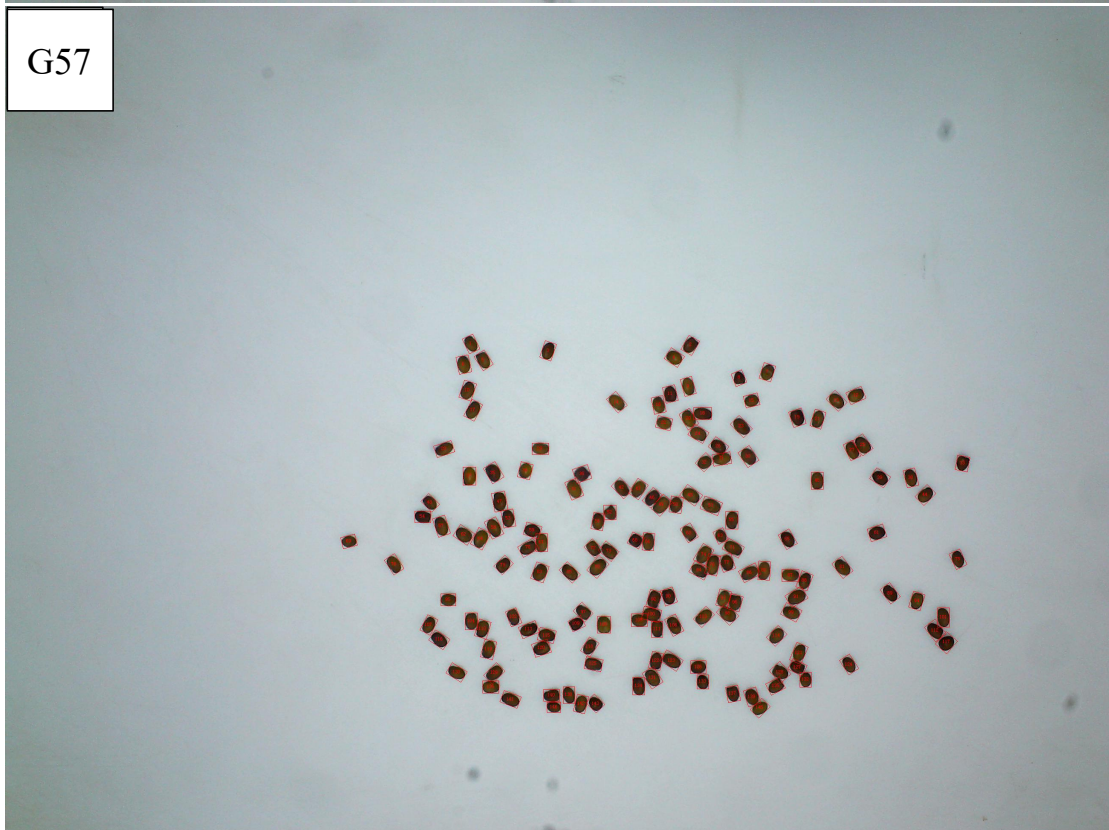

G58

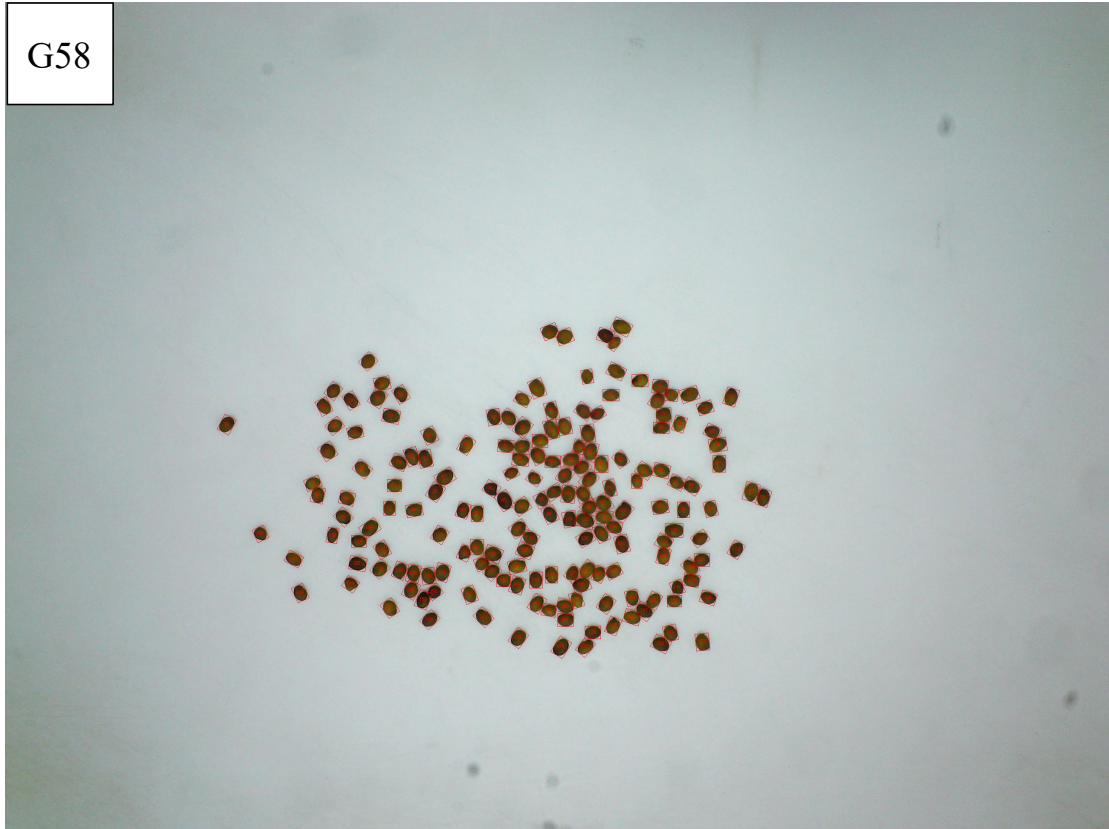

G59

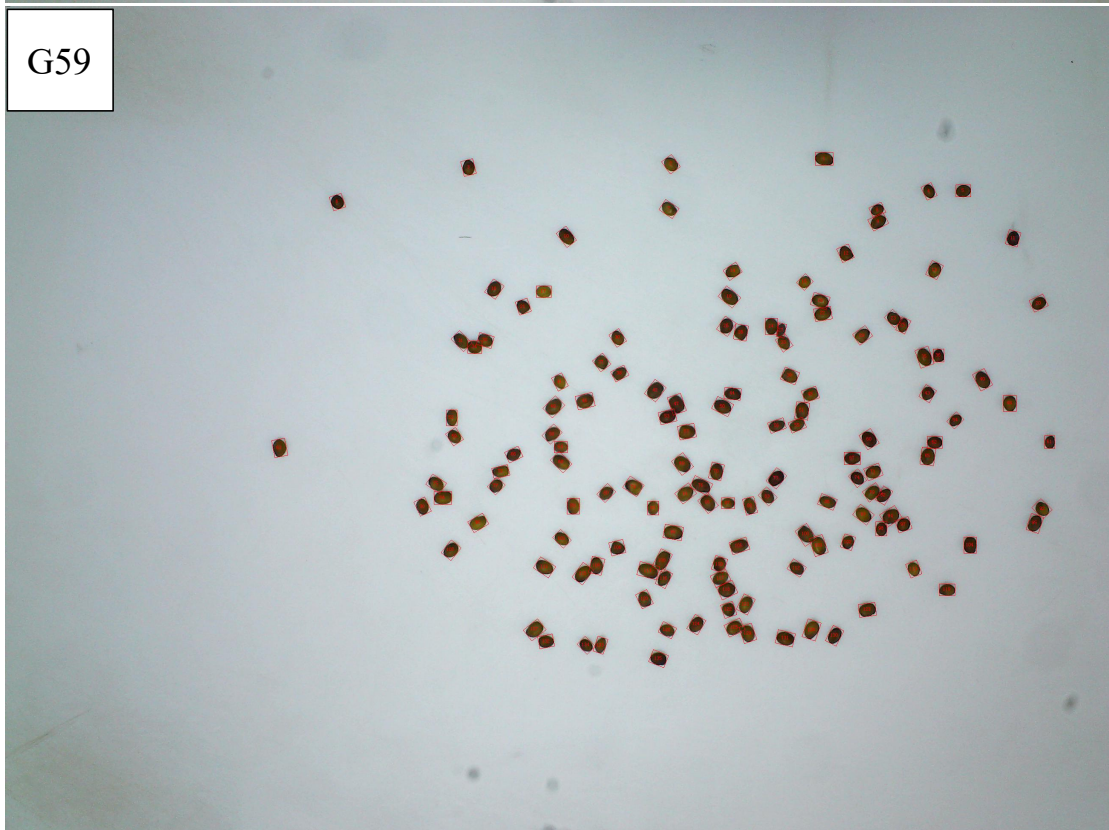

G60

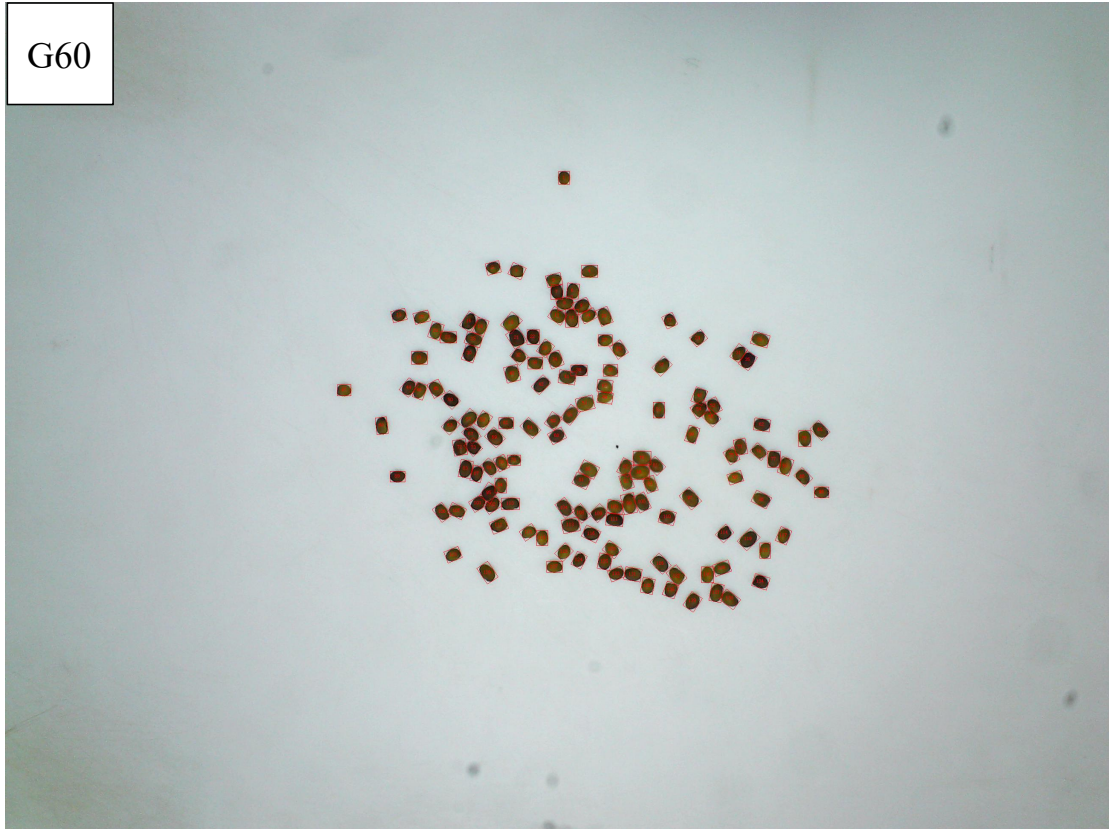

G65

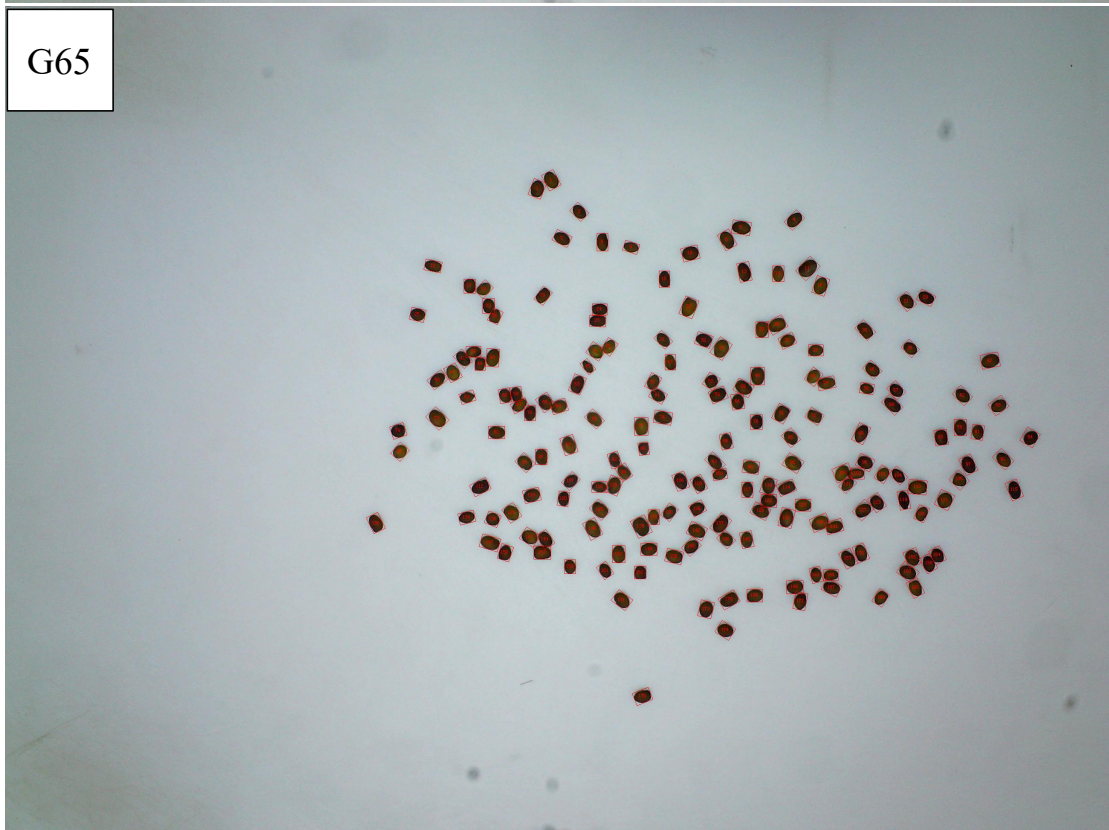

Supplement: Supplementary Table 3 — 22 bioclimatic variables in the 10 populations of S. alopecuroides. [file DataSheet2.zip › Supplementary file 1/HTGY.pdf]

G23

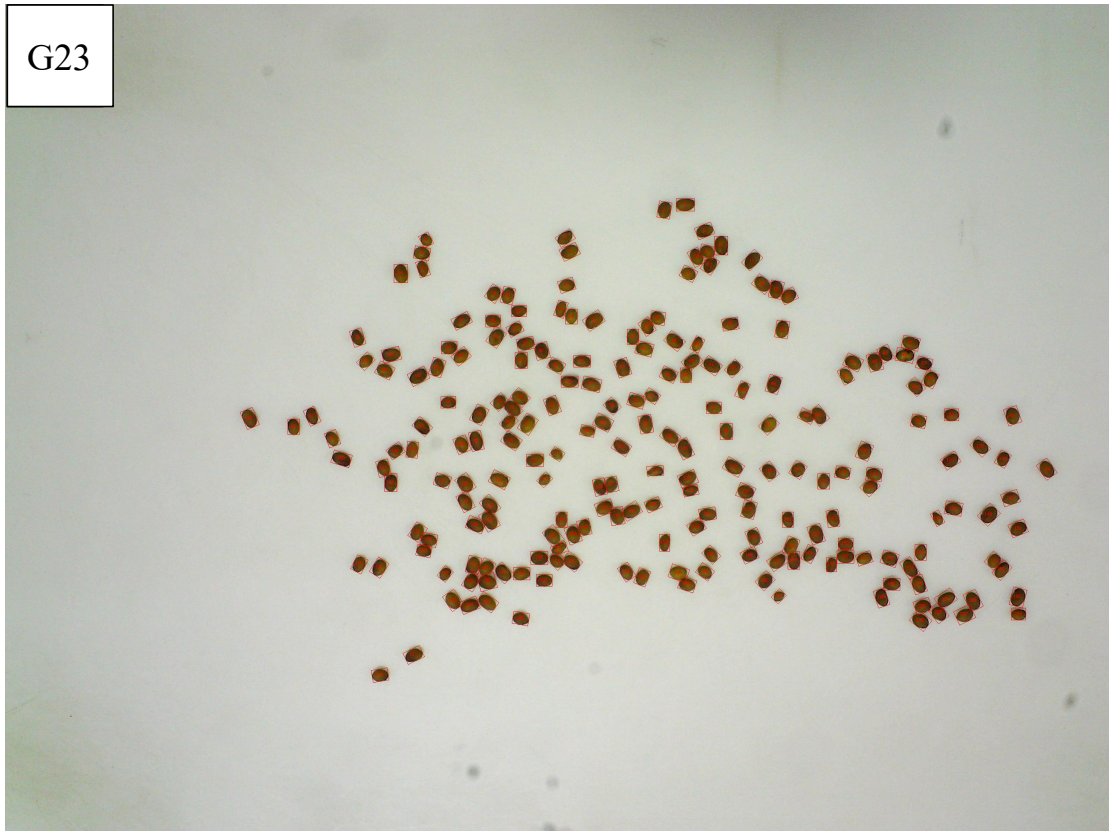

G24

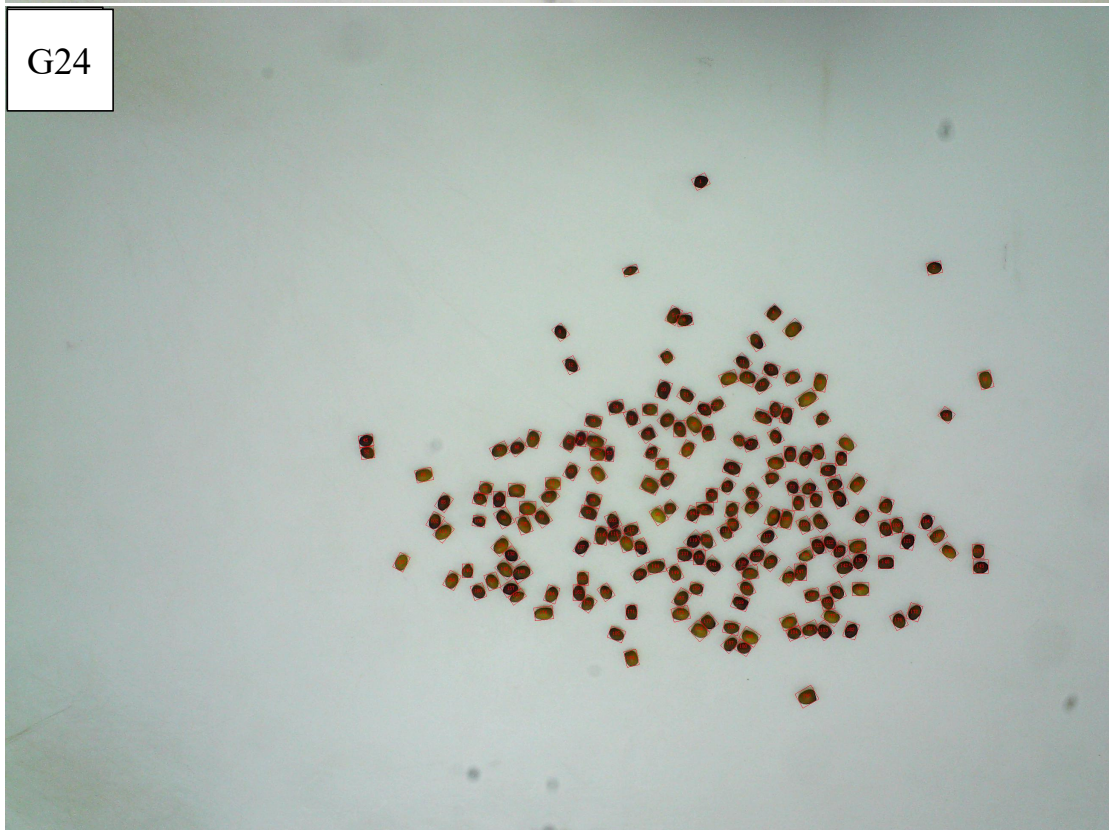

G28

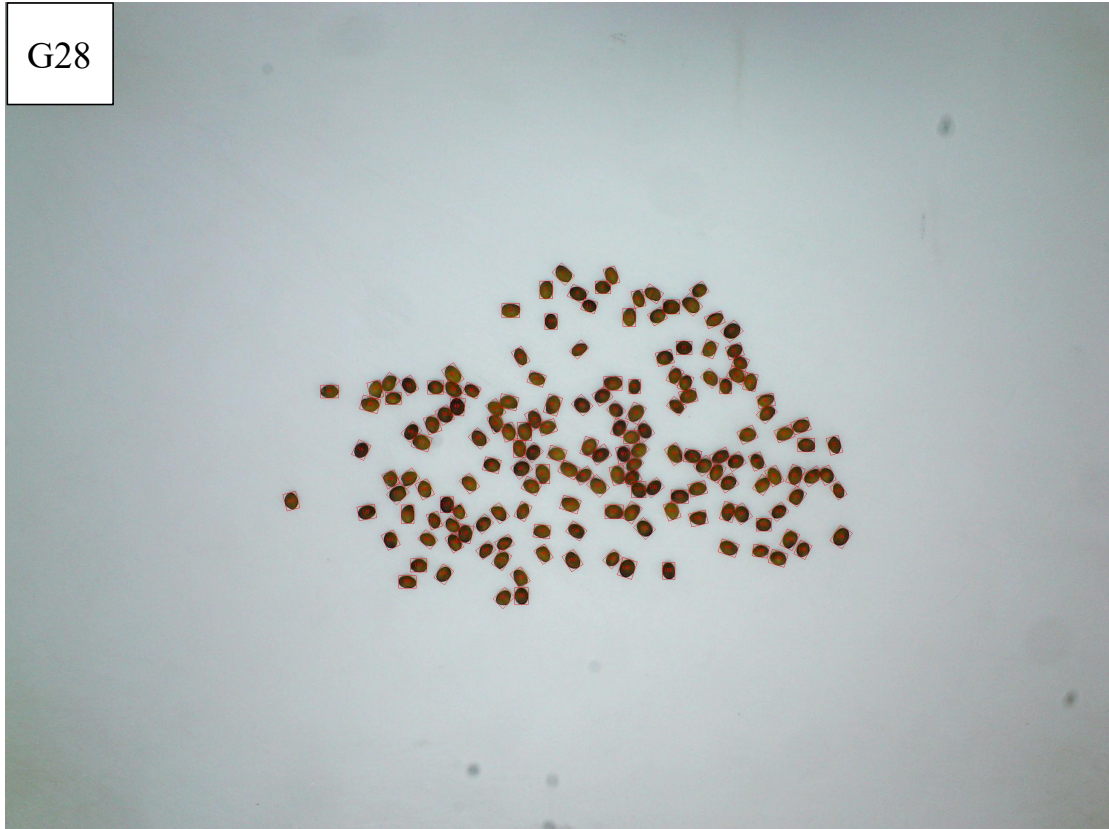

G29

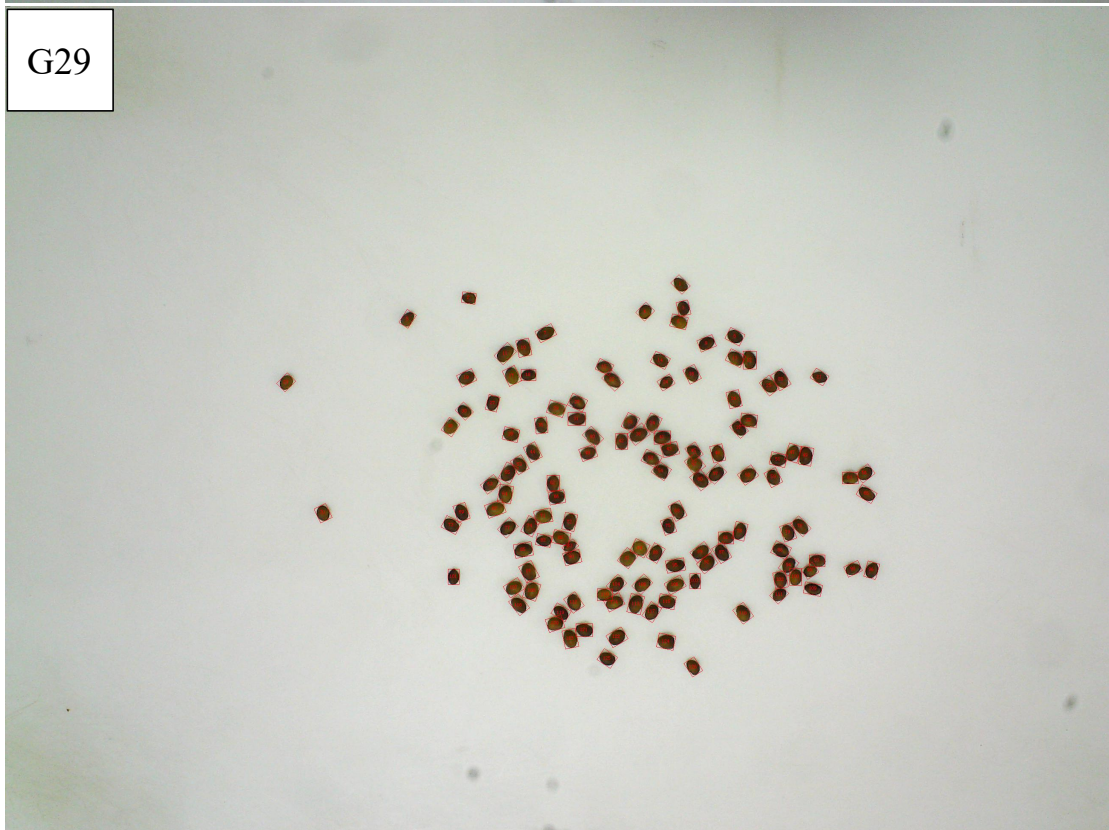

G30

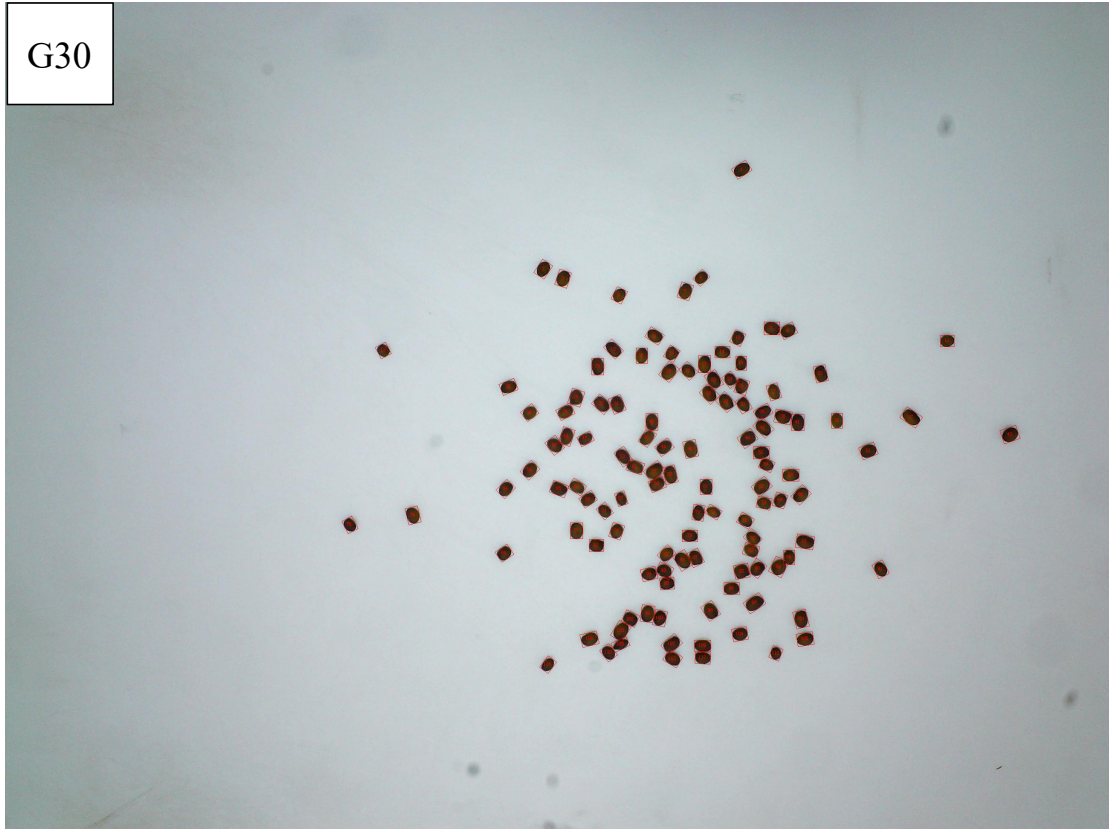

G37

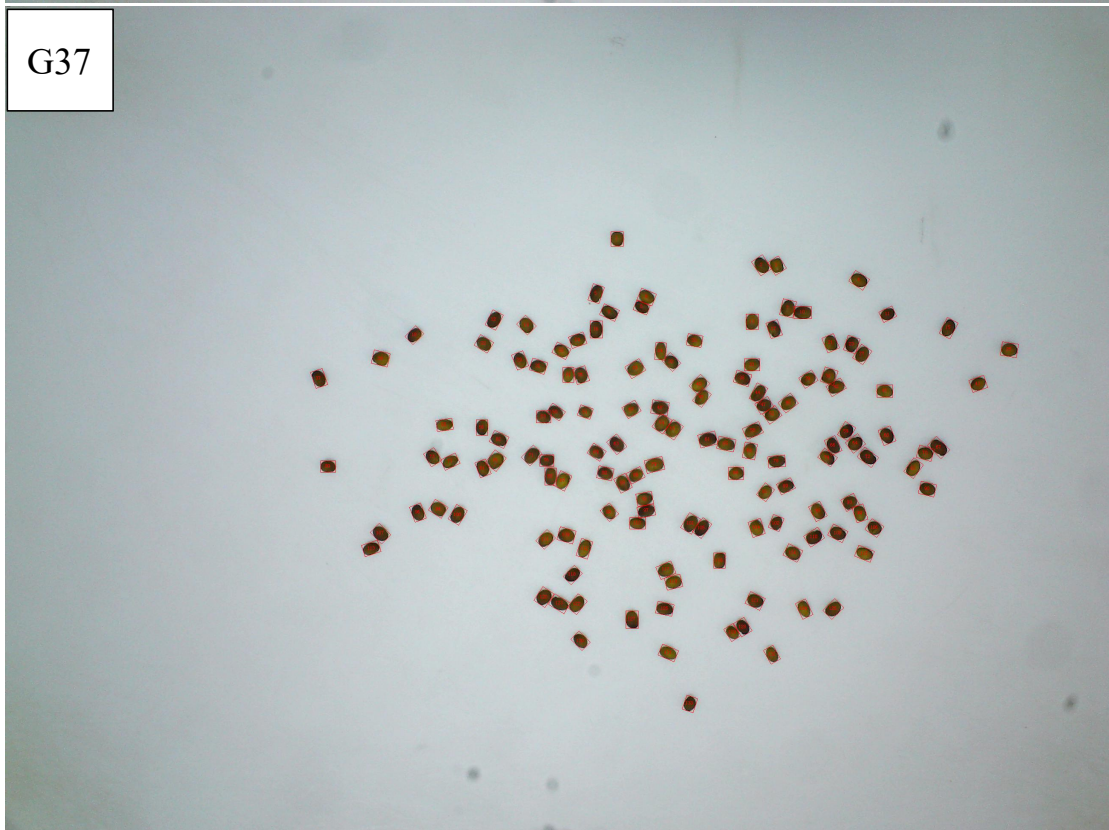

G38

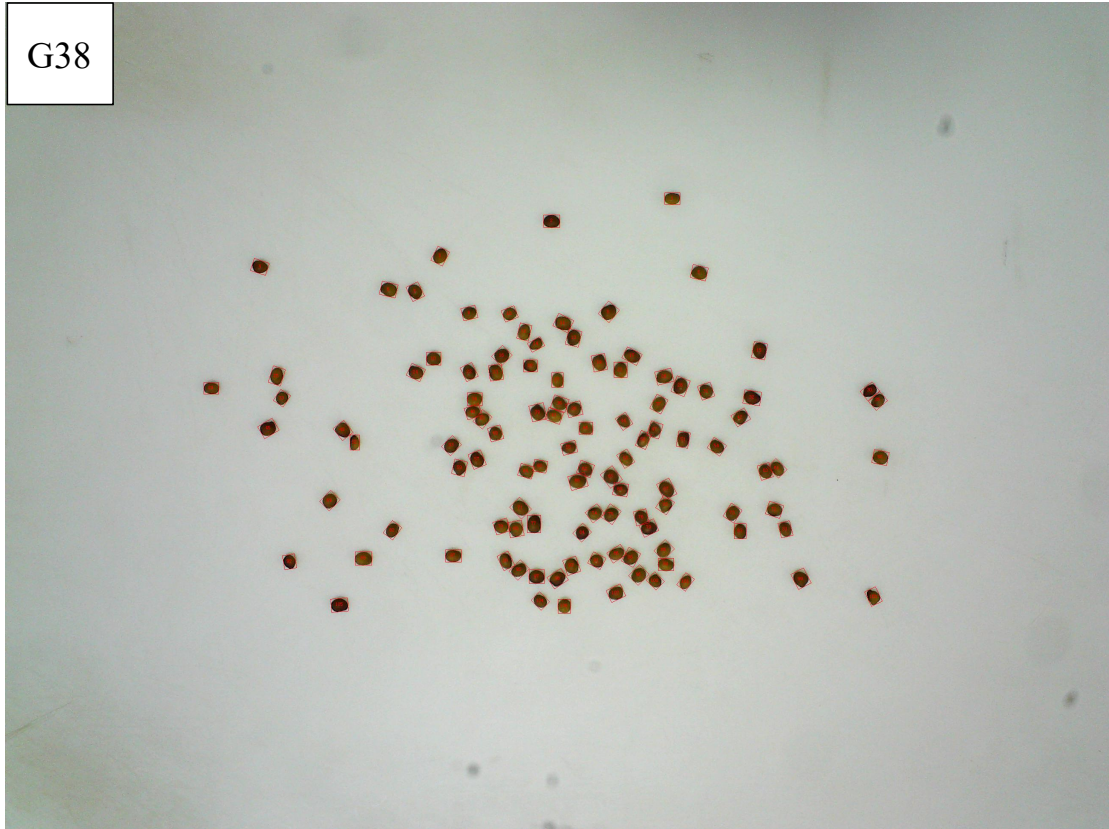

G39

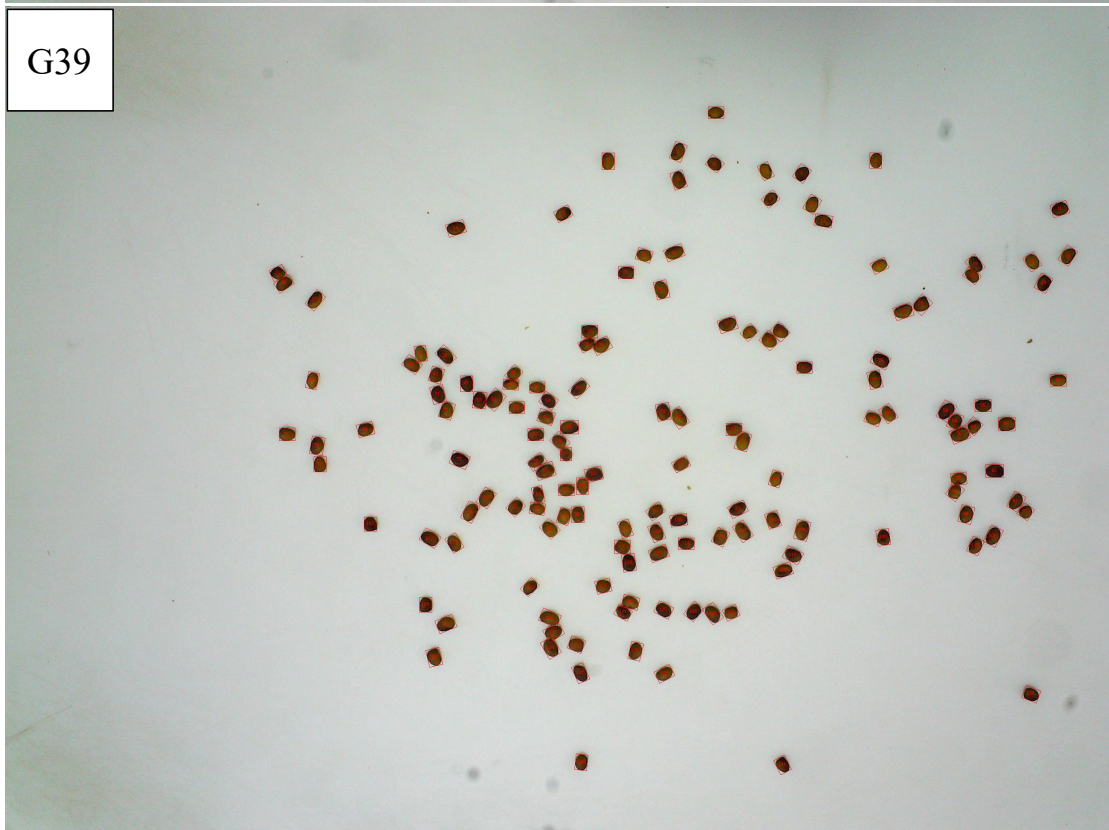

G40

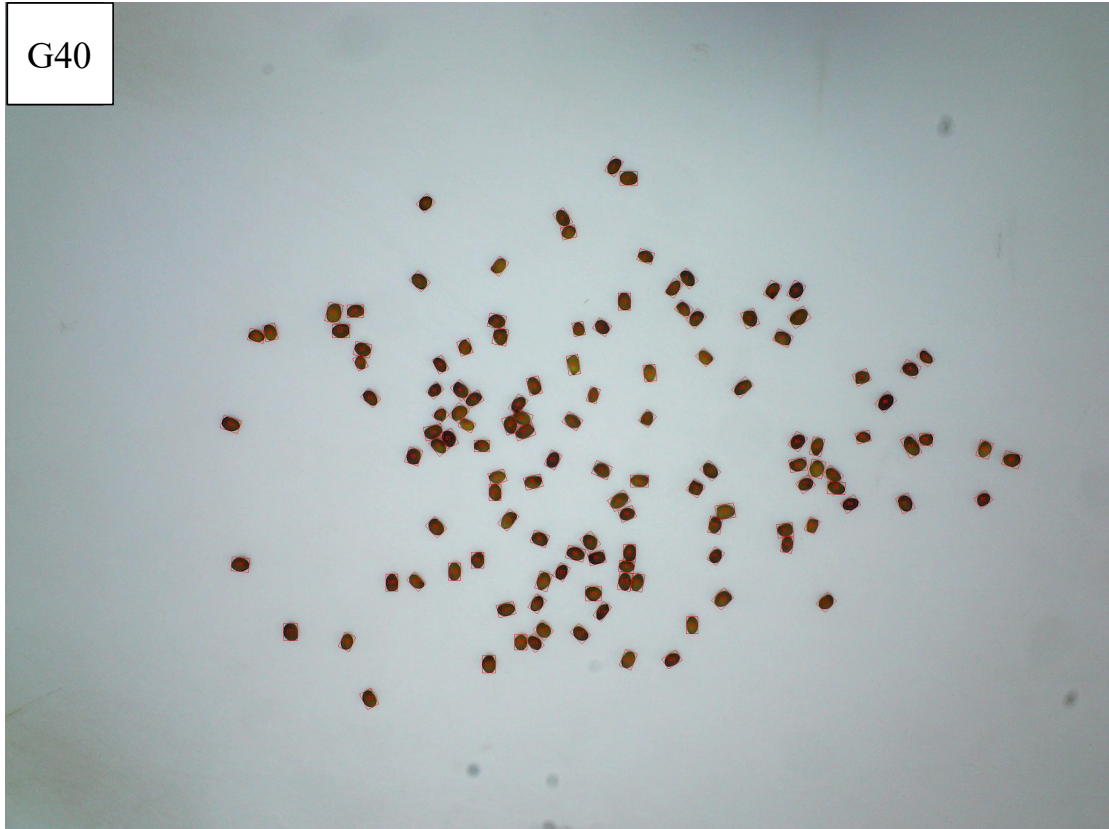

G41

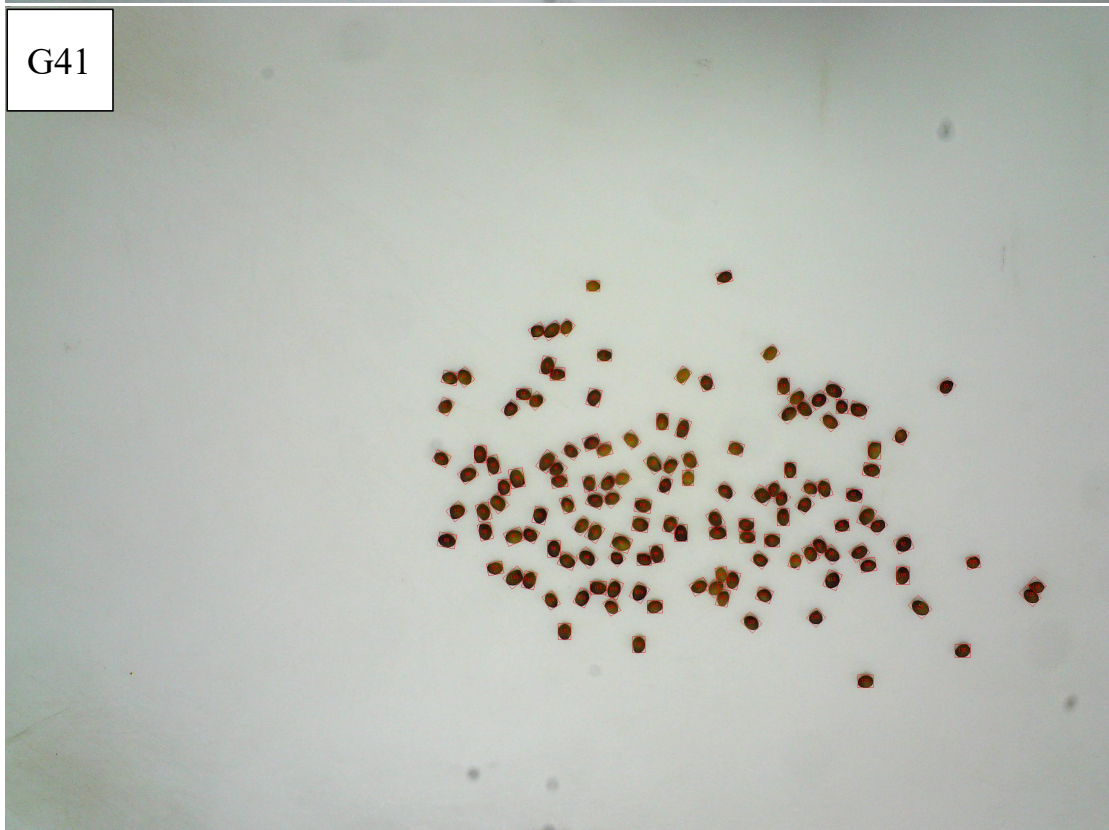

G42

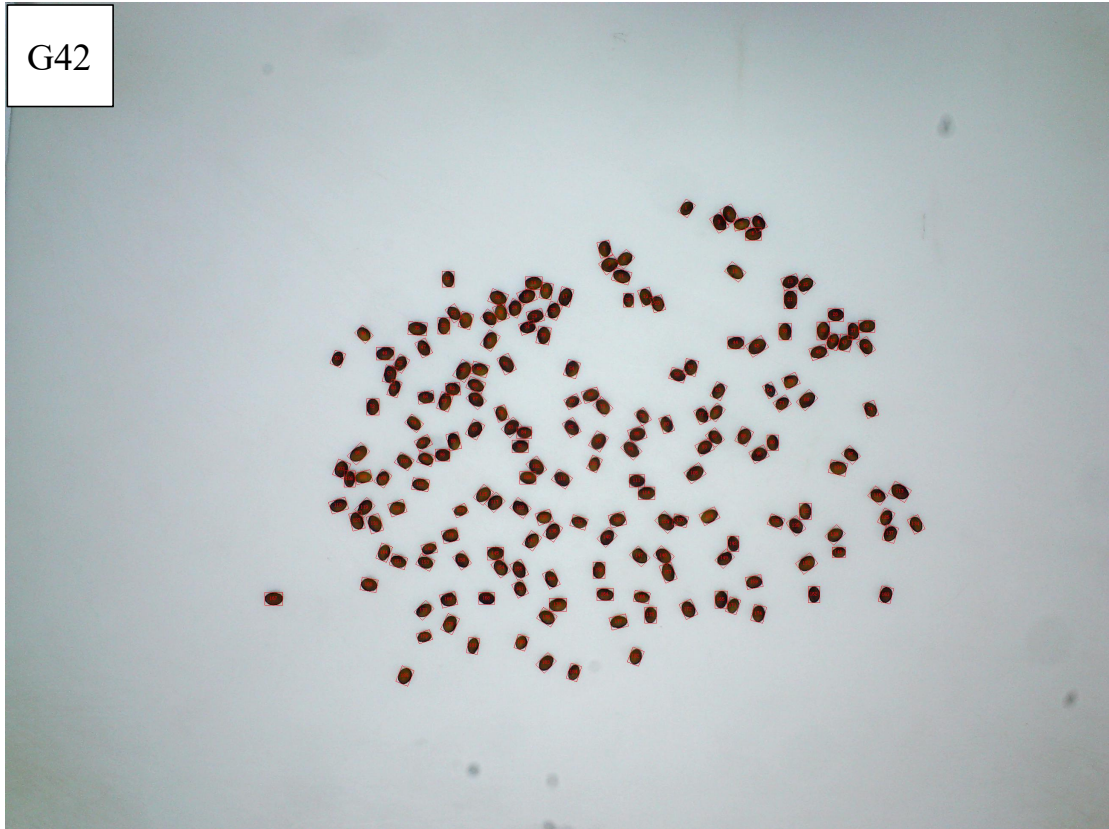

G43

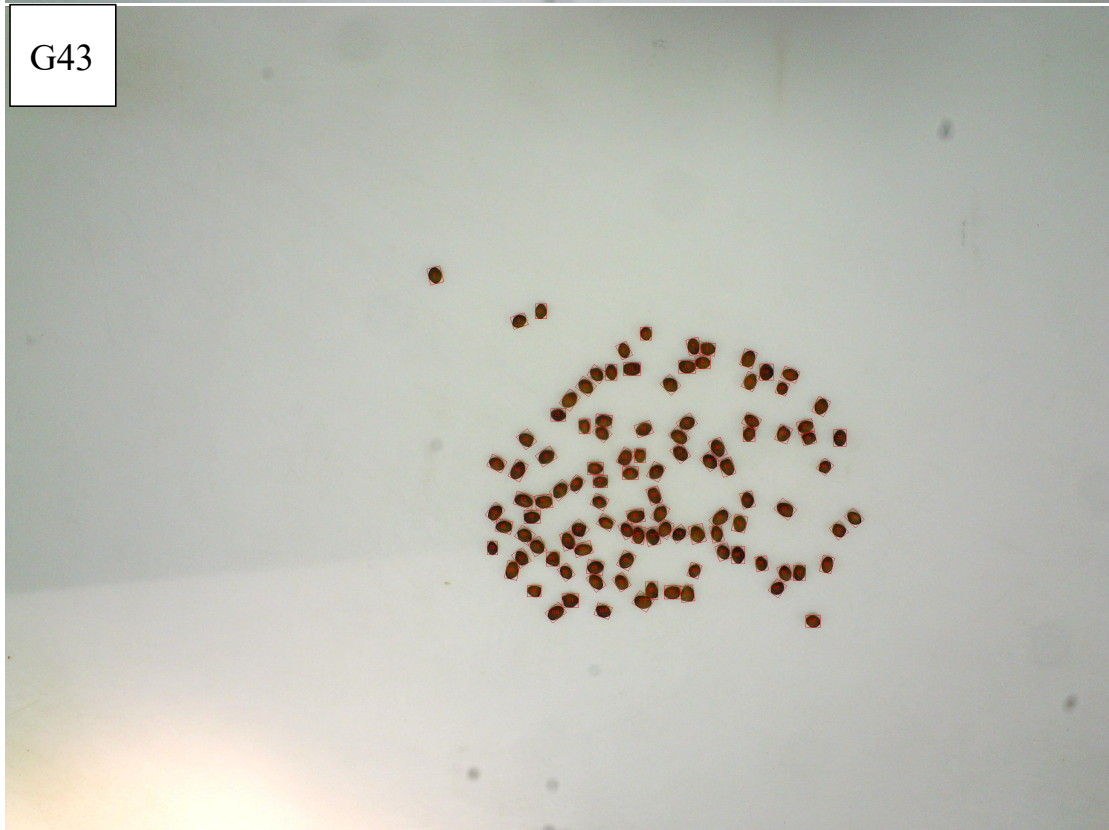

G44

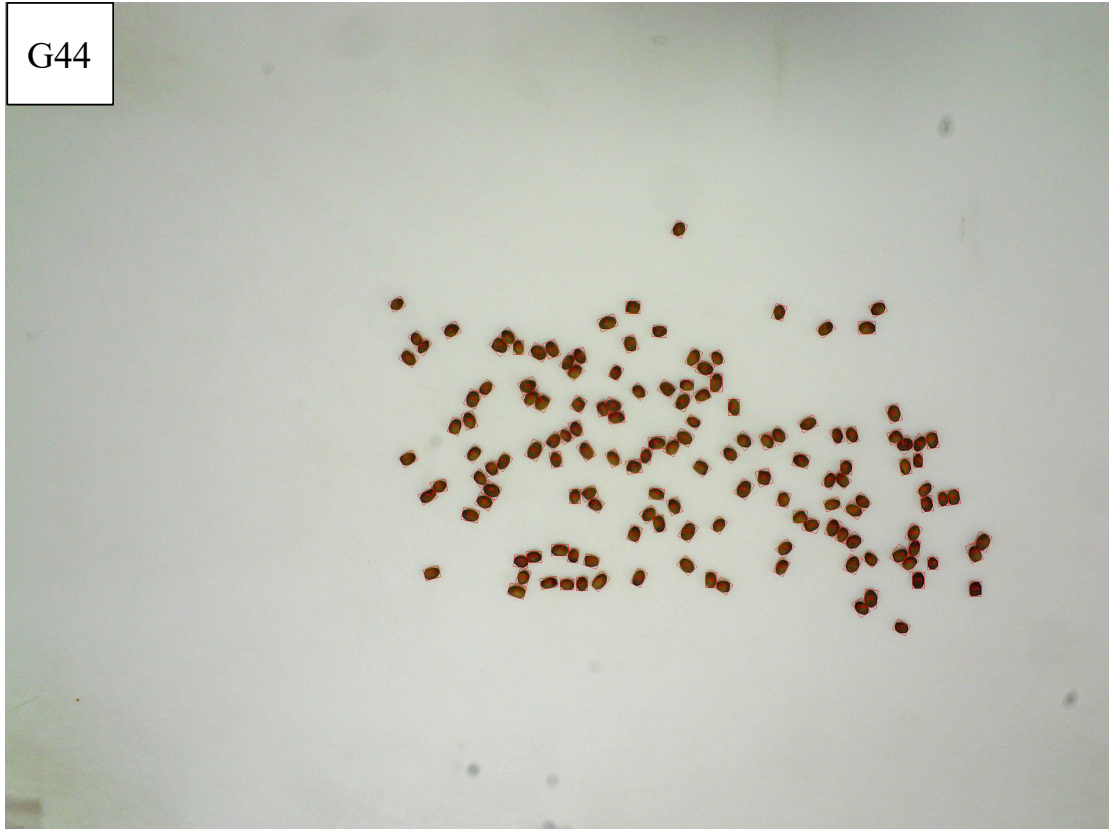

G46

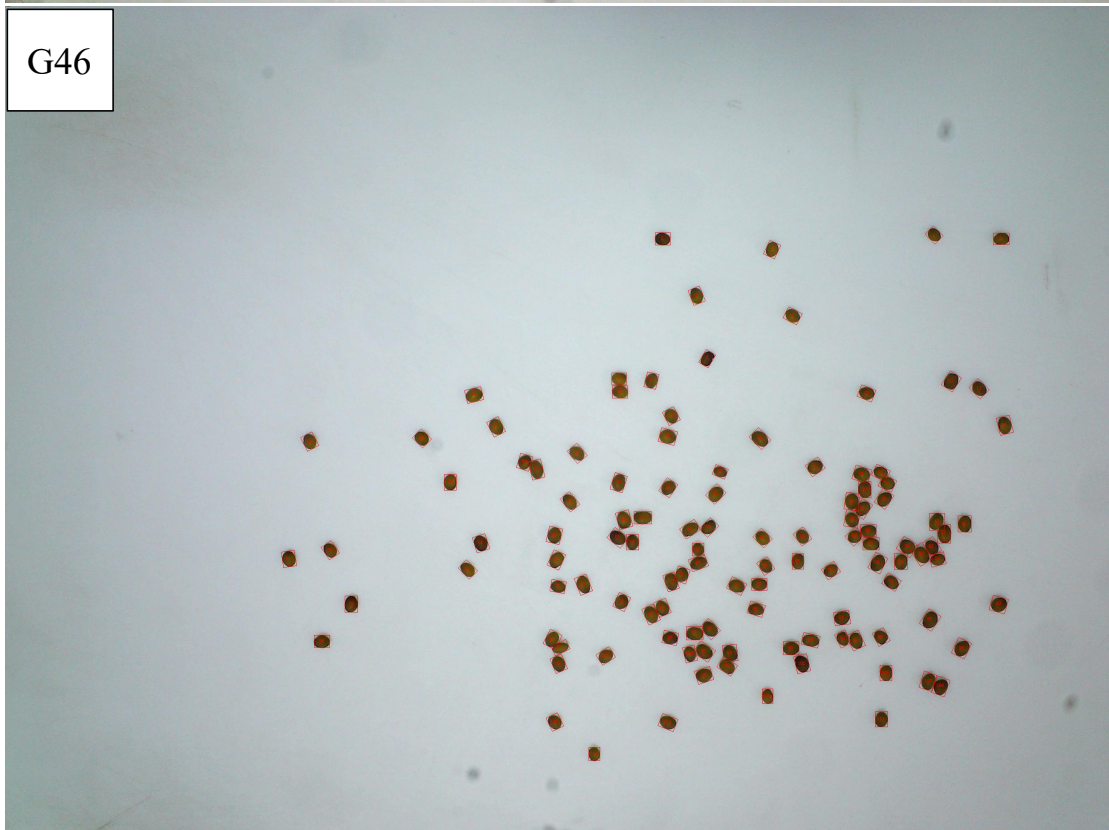

Supplement: Supplementary Table 3 — 22 bioclimatic variables in the 10 populations of S. alopecuroides. [file DataSheet2.zip › Supplementary file 1/KLSM.pdf]

G61

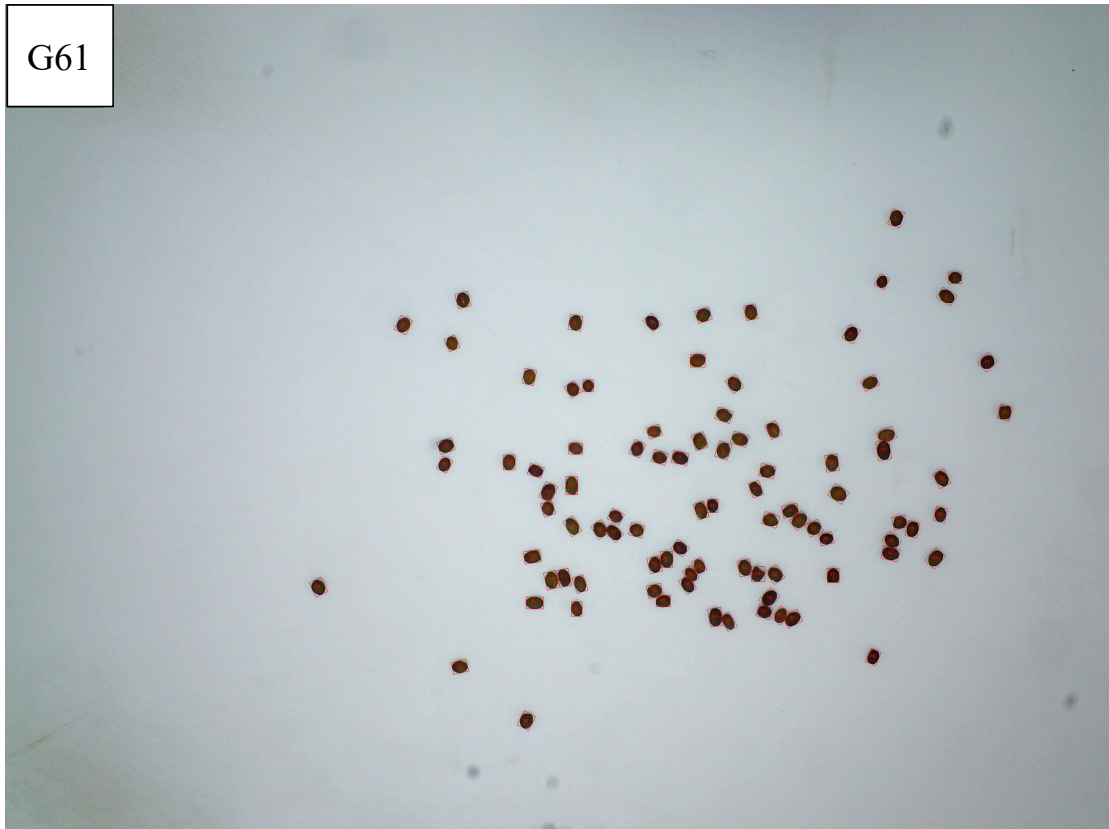

G62

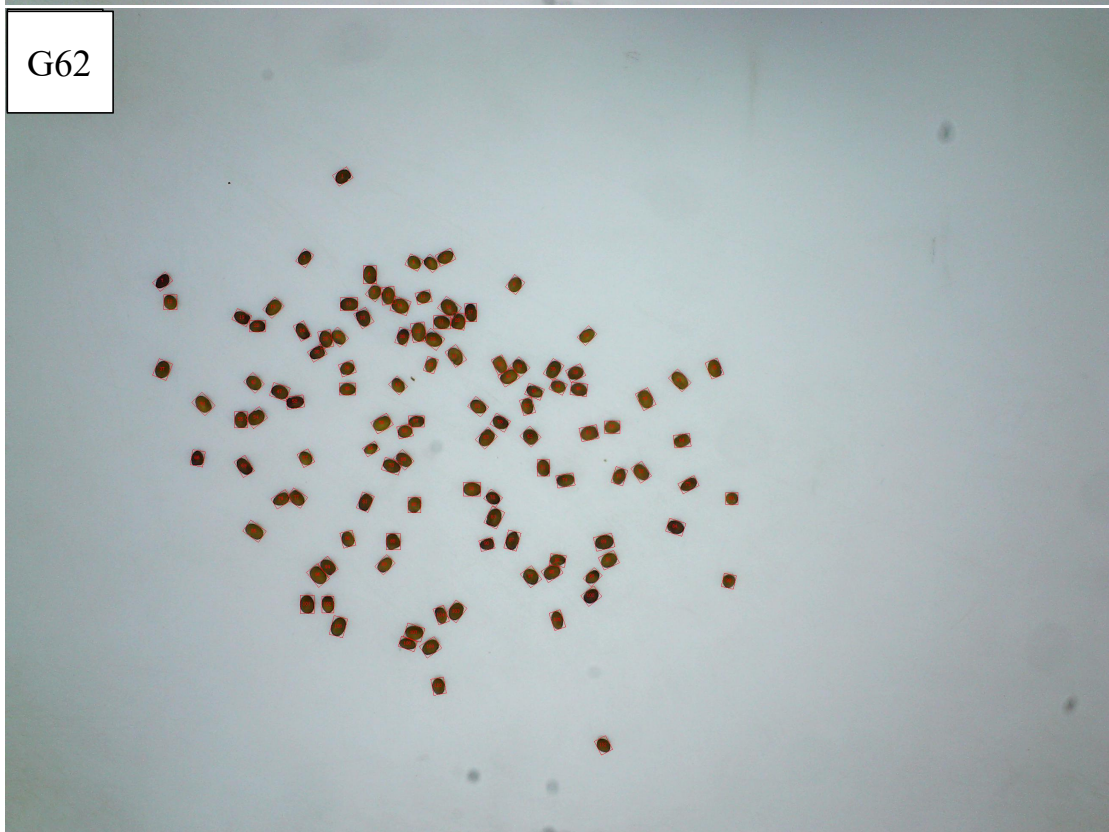

G63

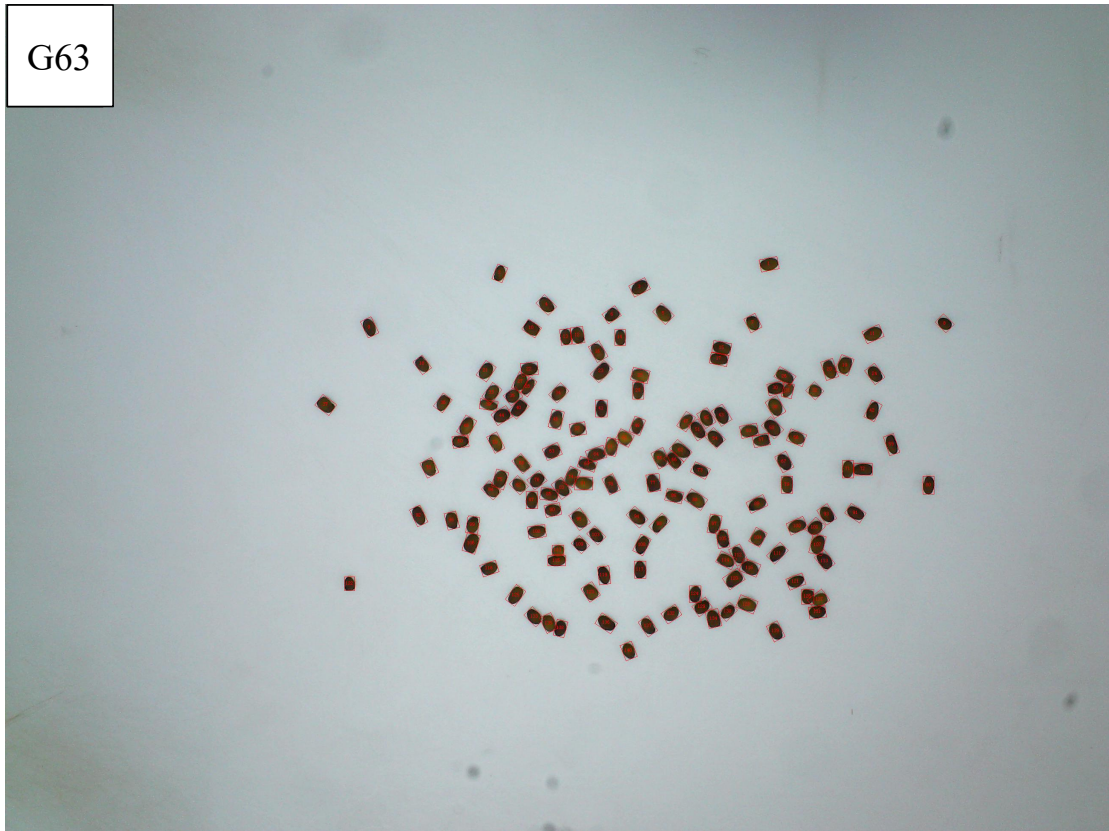

G64

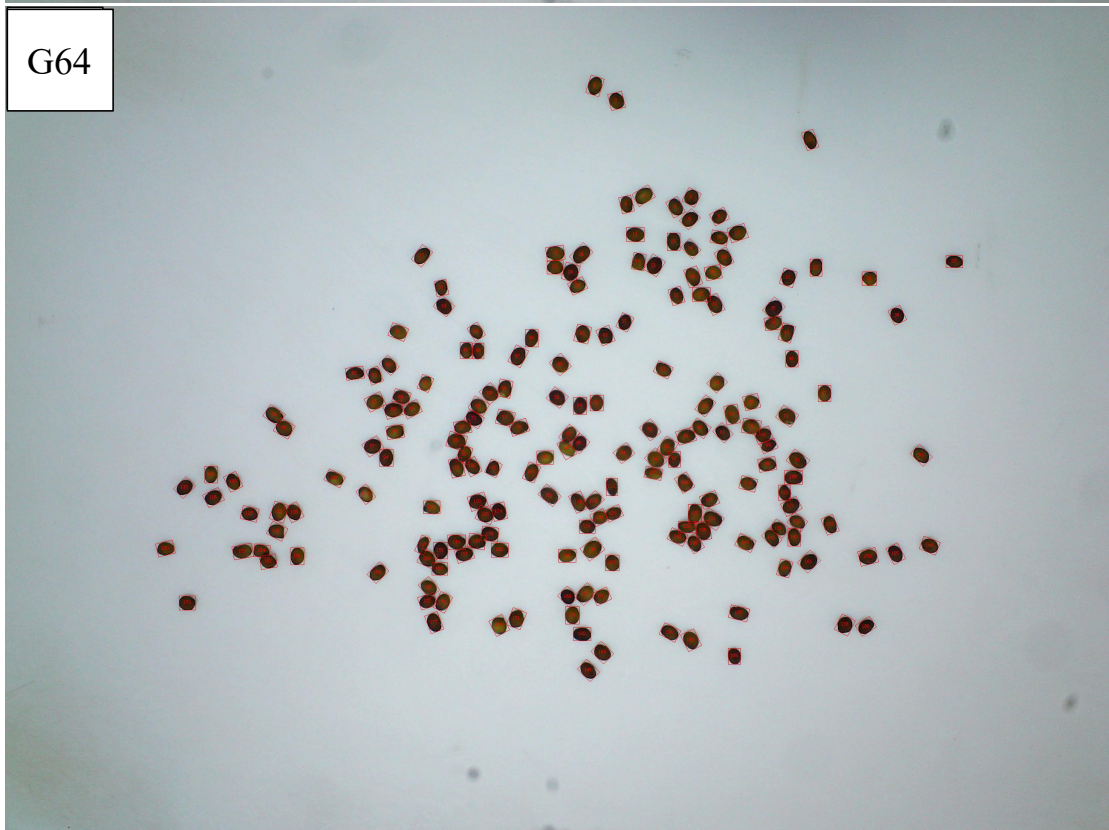

Supplement: Supplementary Table 3 — 22 bioclimatic variables in the 10 populations of S. alopecuroides. [file DataSheet2.zip › Supplementary file 1/NMGY.pdf]

G52

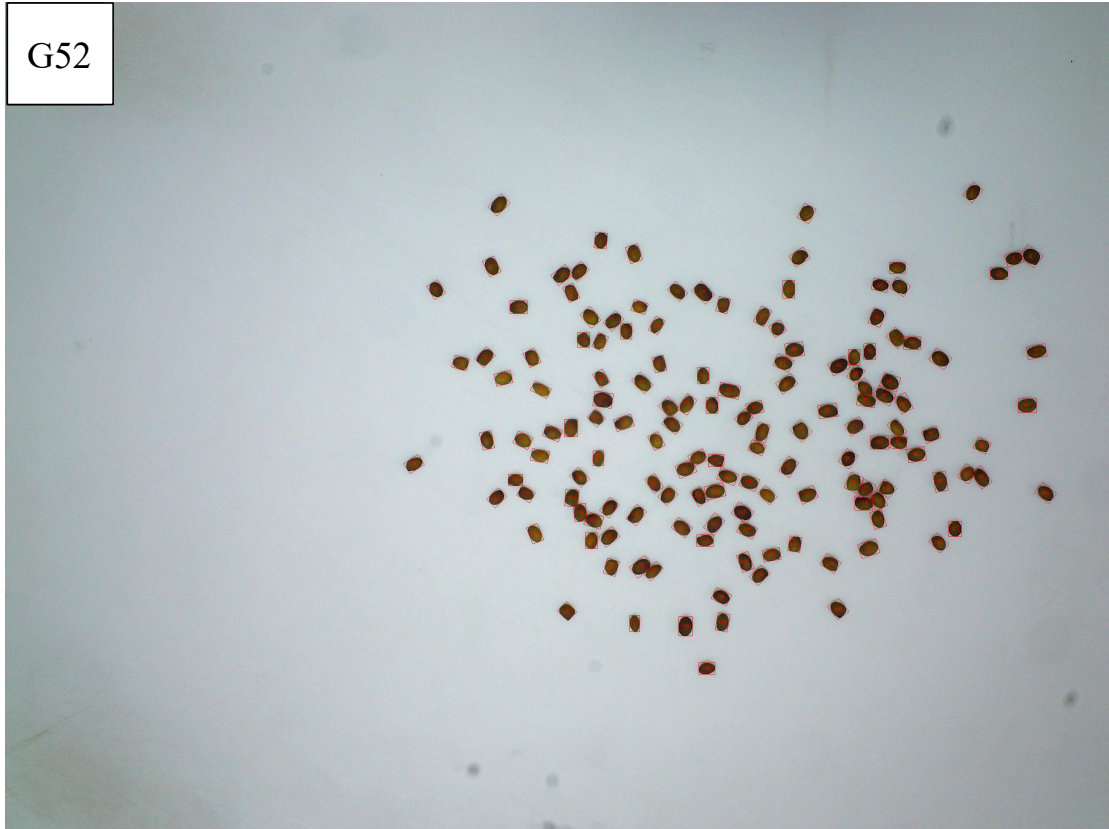

G53

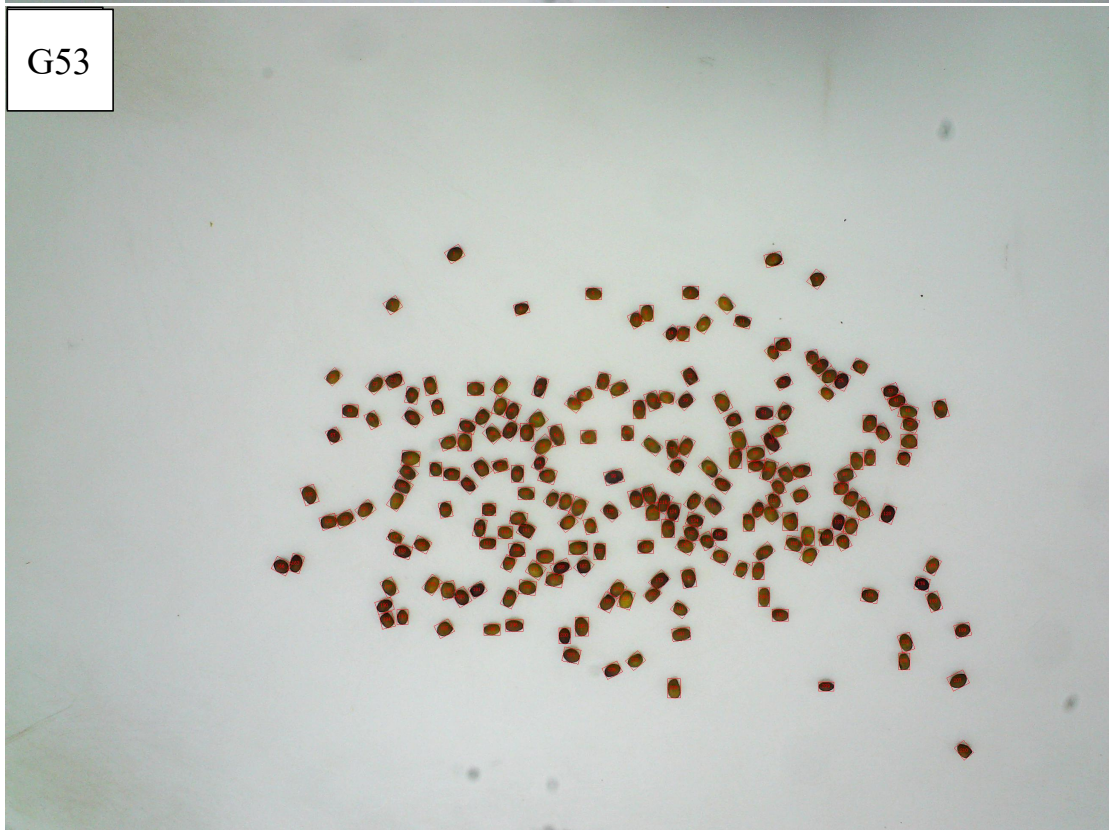

G54

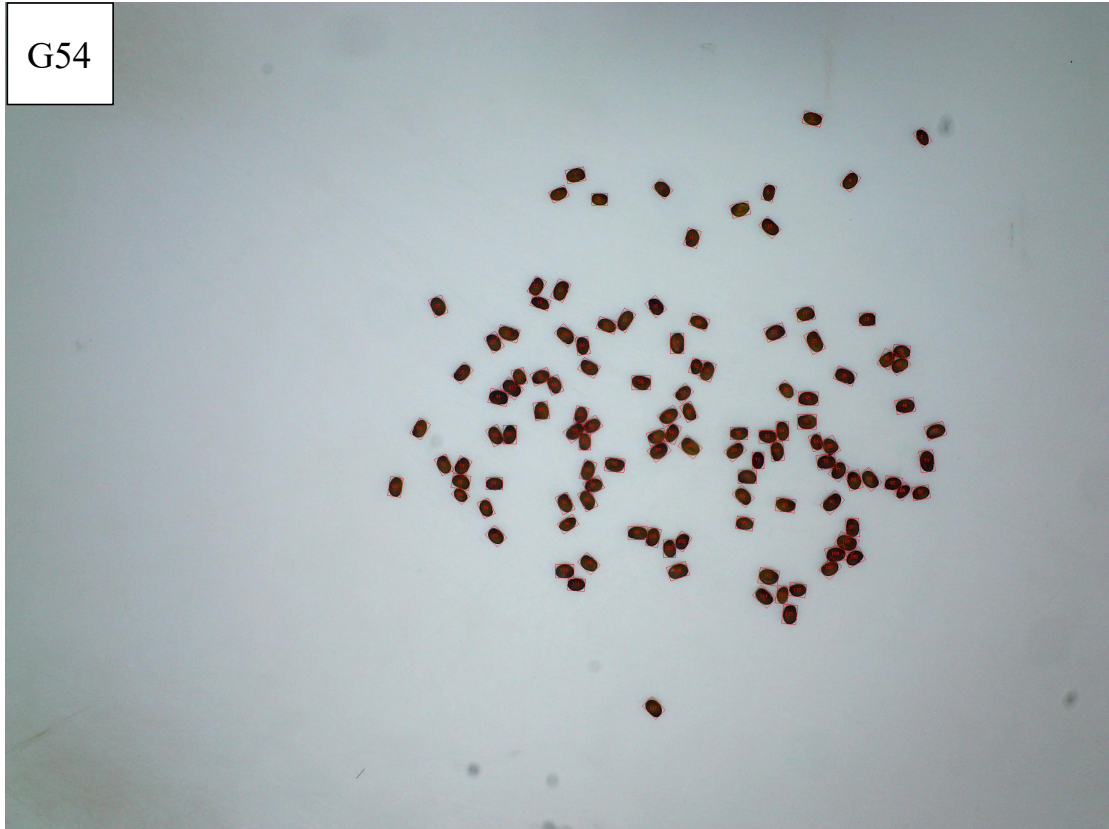

G55

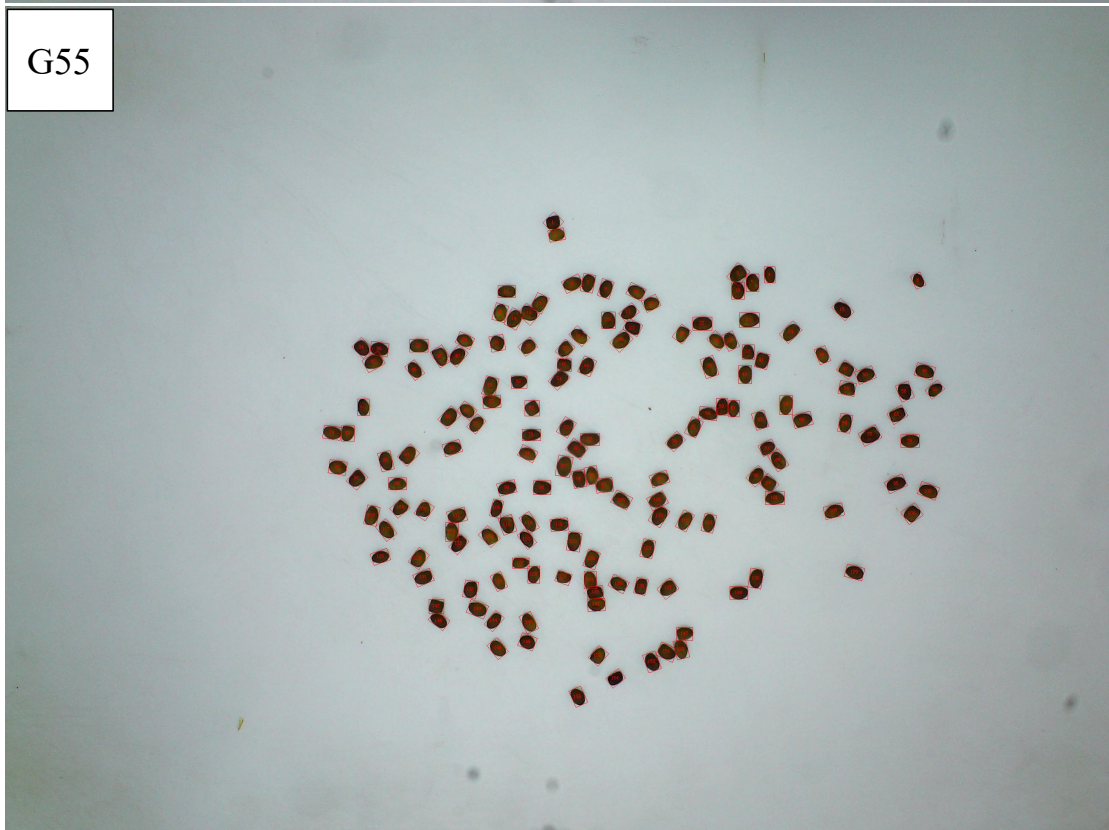

Supplement: Supplementary Table 3 — 22 bioclimatic variables in the 10 populations of S. alopecuroides. [file DataSheet2.zip › Supplementary file 1/QLSM.pdf]

G8

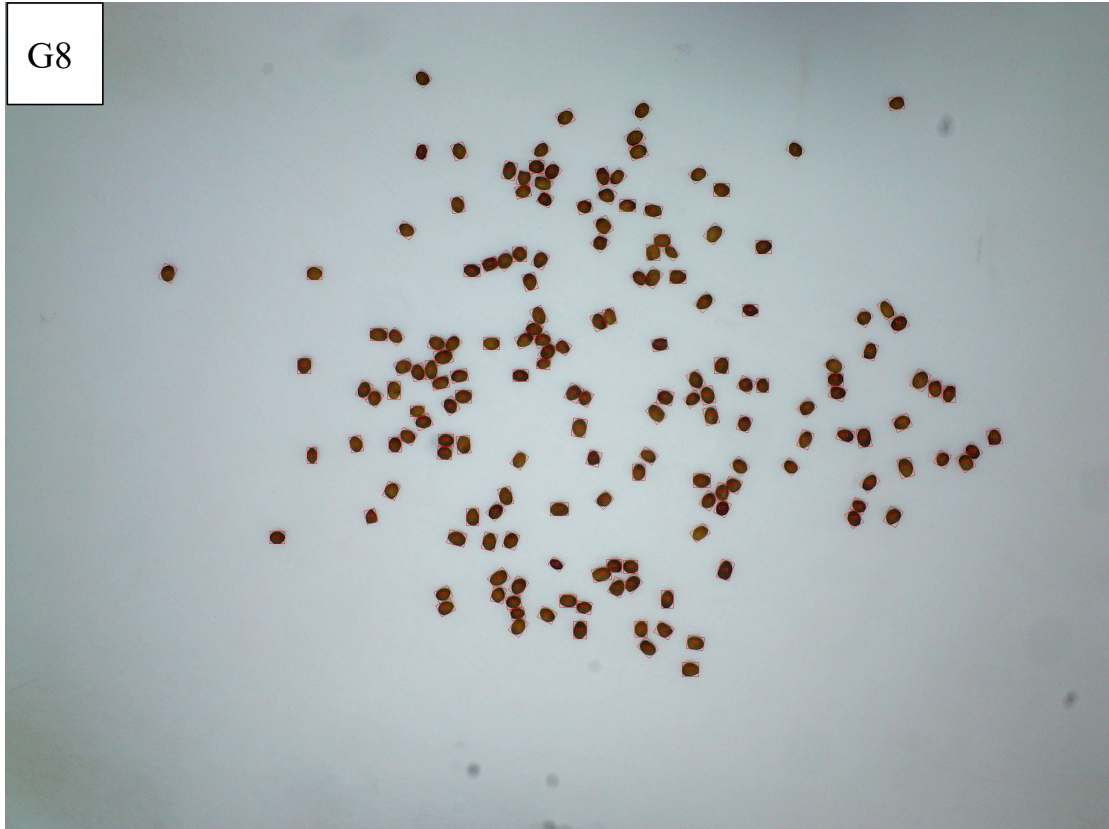

G9

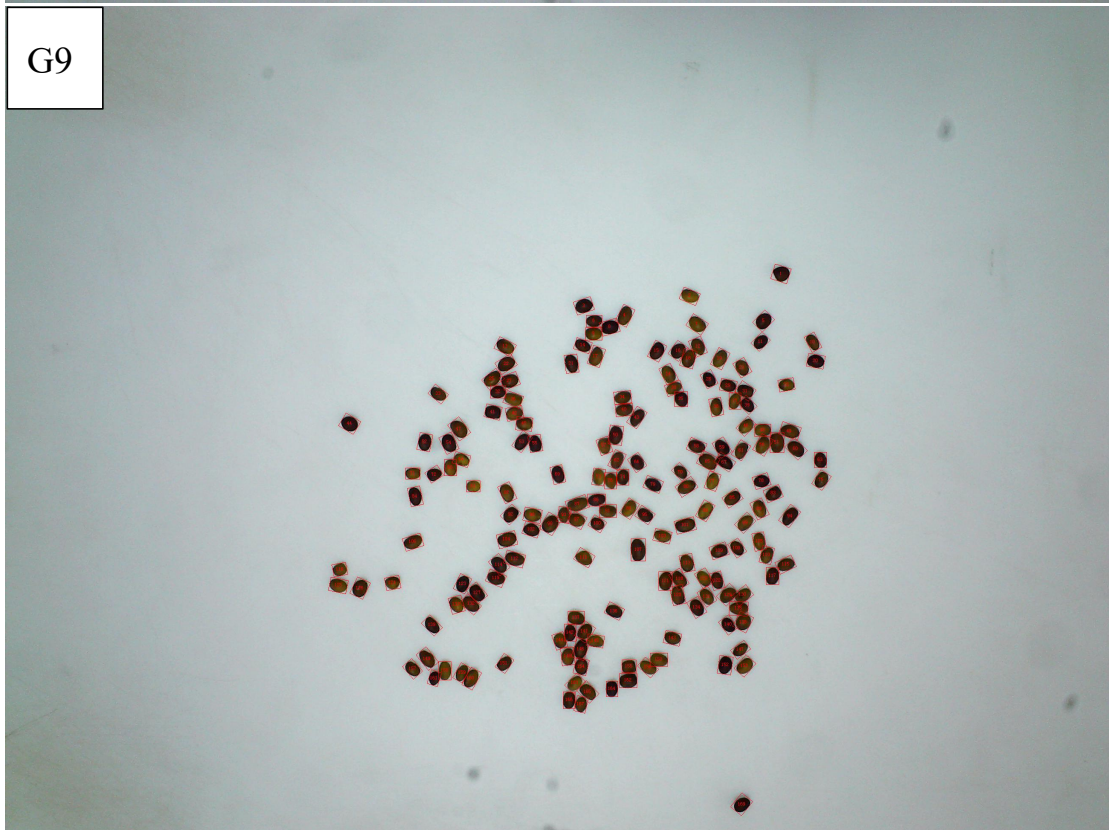

G10

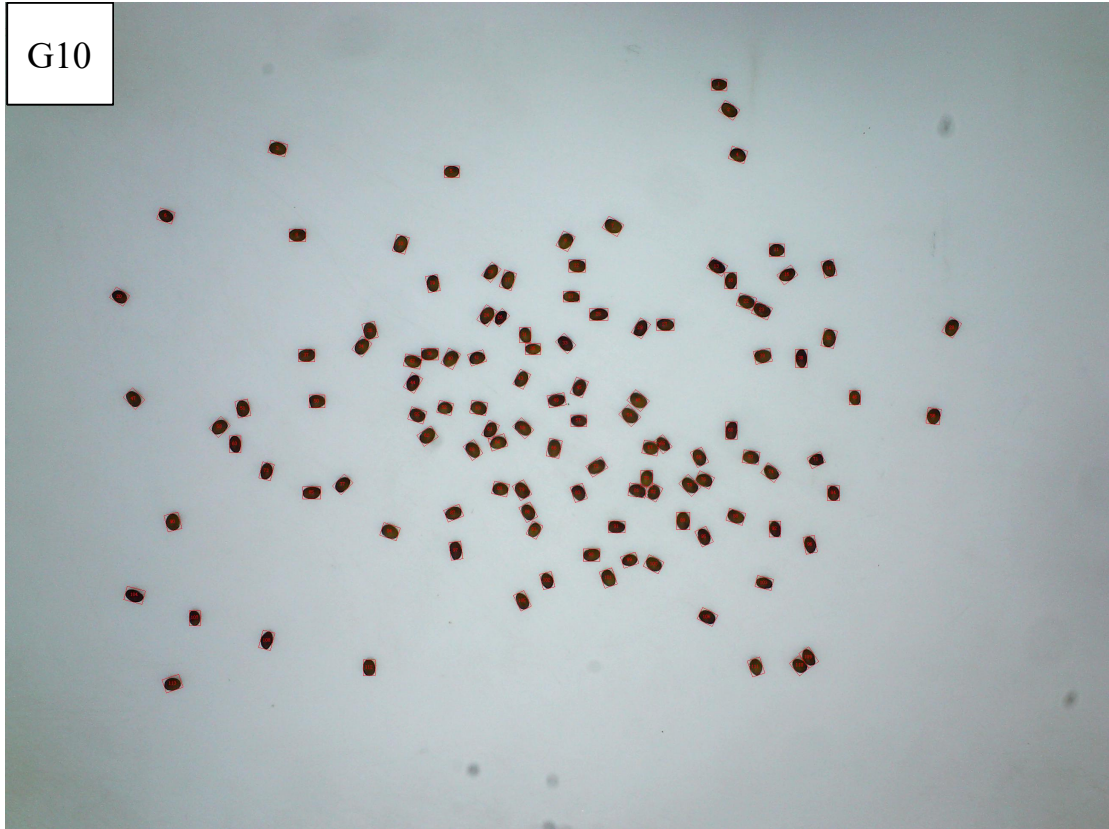

G11

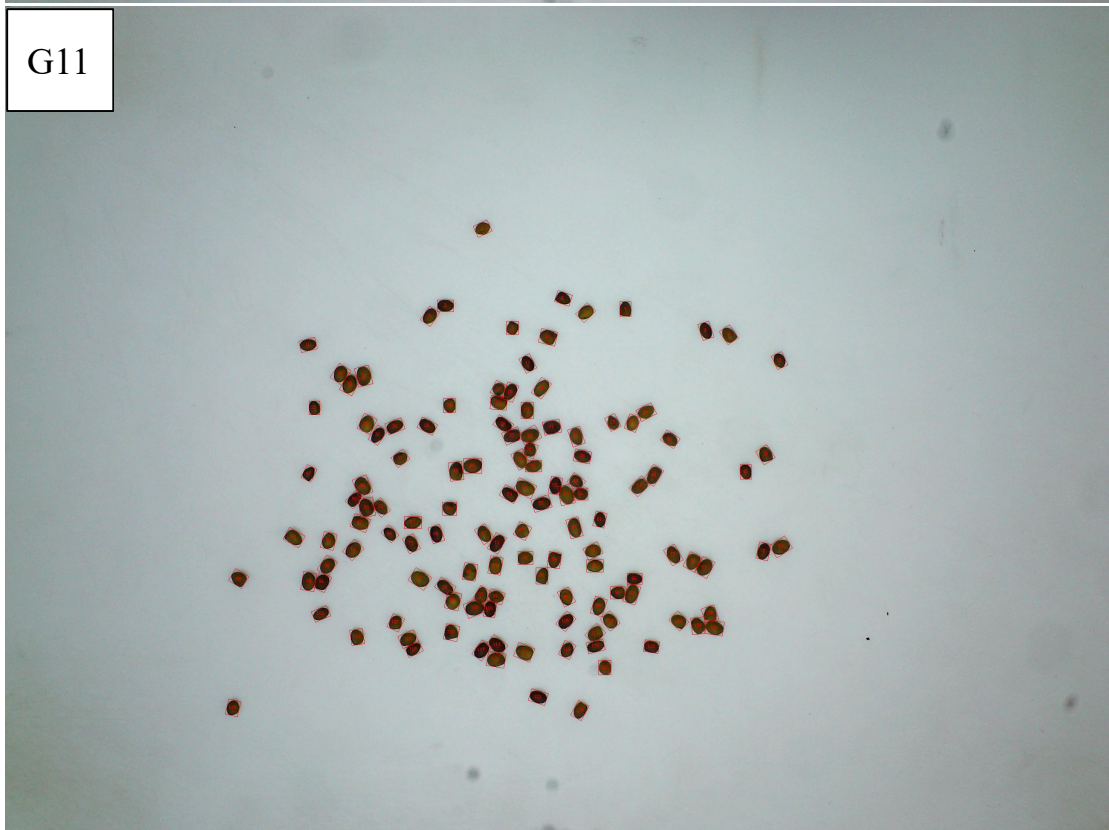

Supplement: Supplementary Table 3 — 22 bioclimatic variables in the 10 populations of S. alopecuroides. [file DataSheet2.zip › Supplementary file 1/TCDQ.pdf]

G47

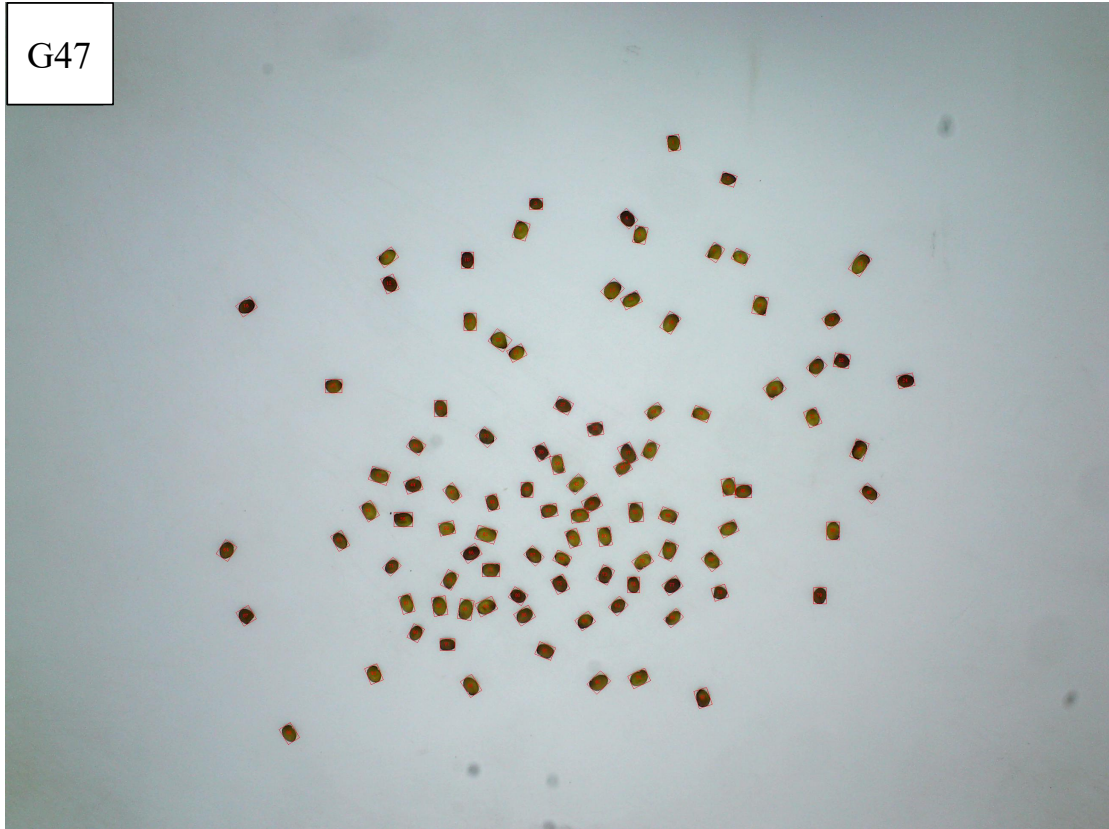

G48

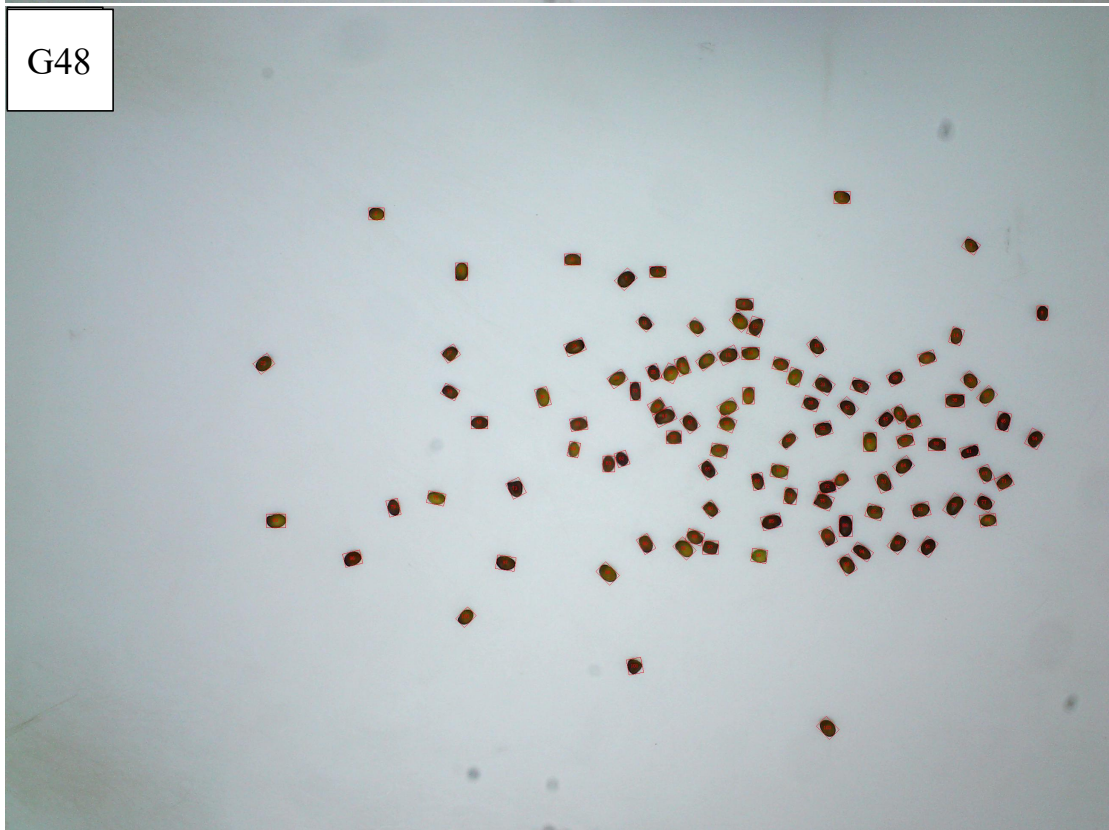

G49

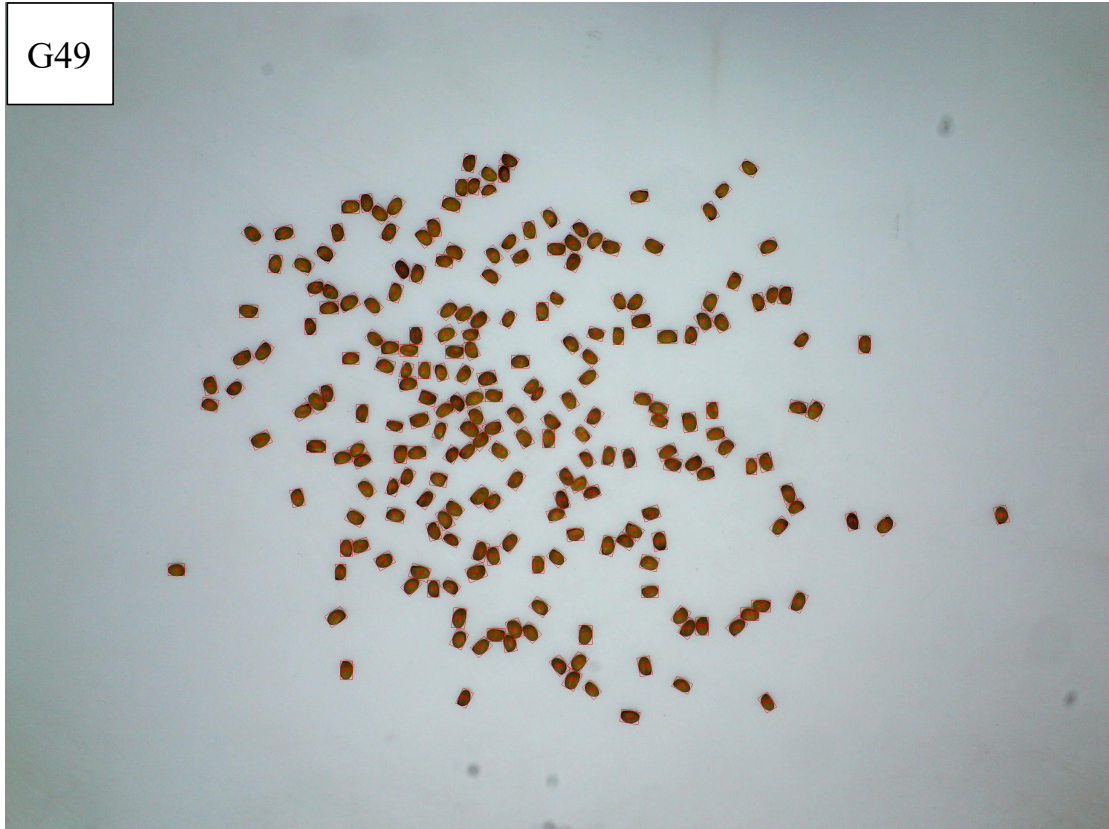

G50

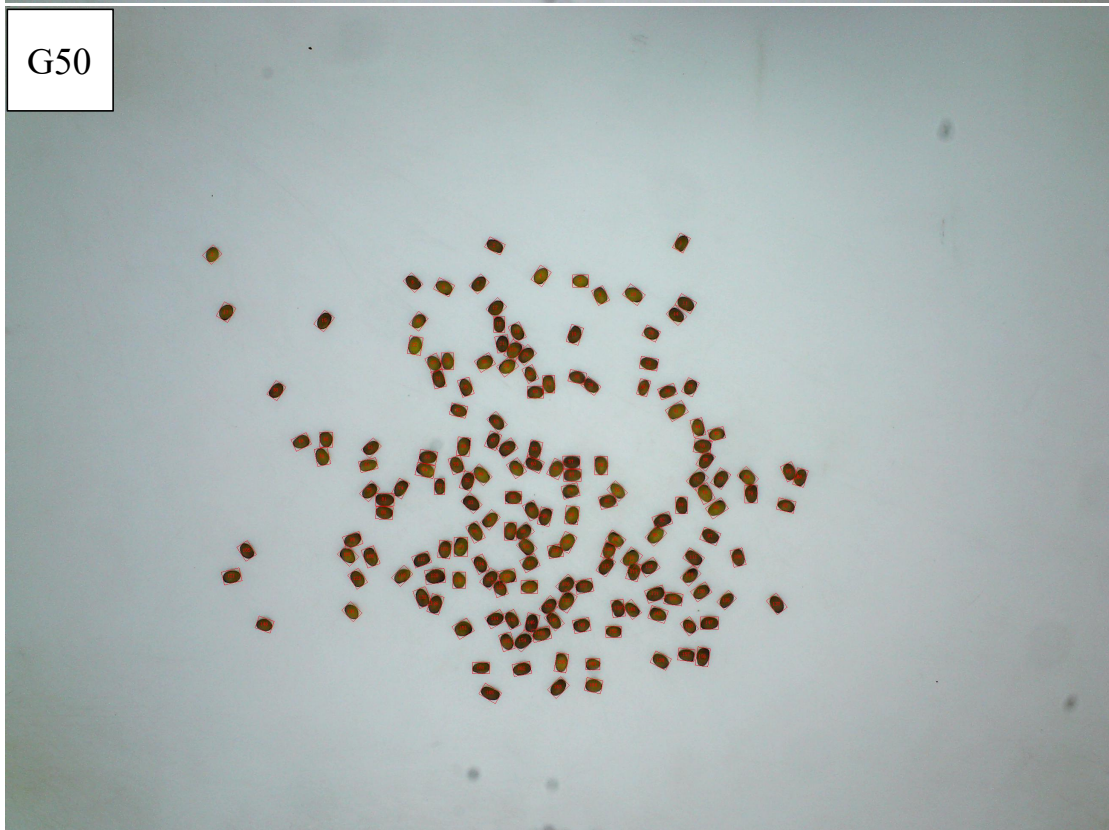

G51

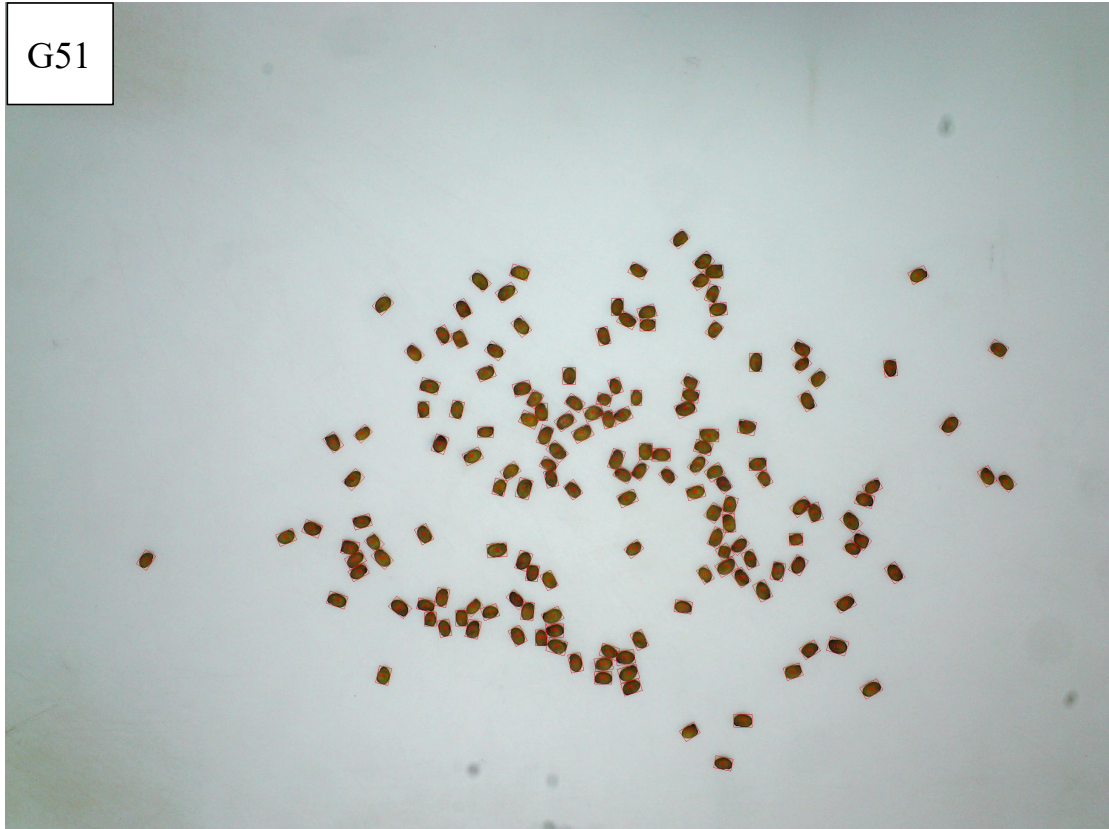

Supplement: Supplementary Table 3 — 22 bioclimatic variables in the 10 populations of S. alopecuroides. [file DataSheet2.zip › Supplementary file 1/THPD.pdf]

G6

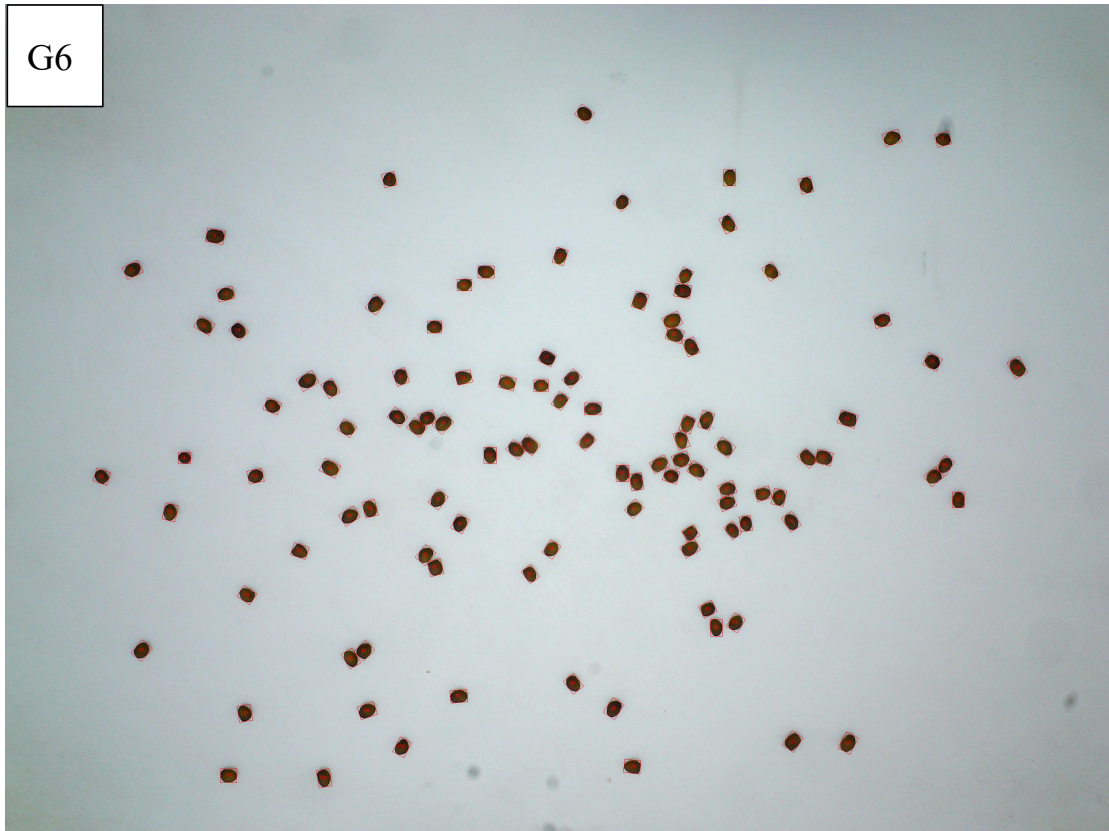

G7

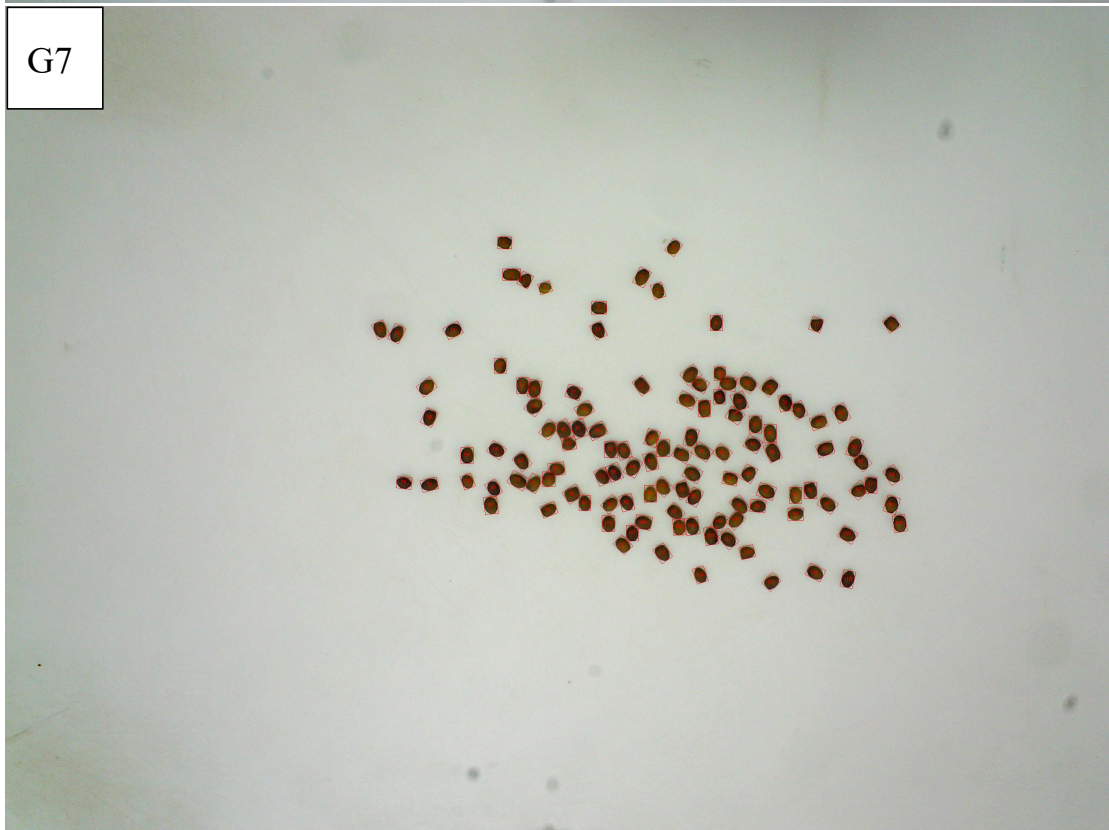

G15

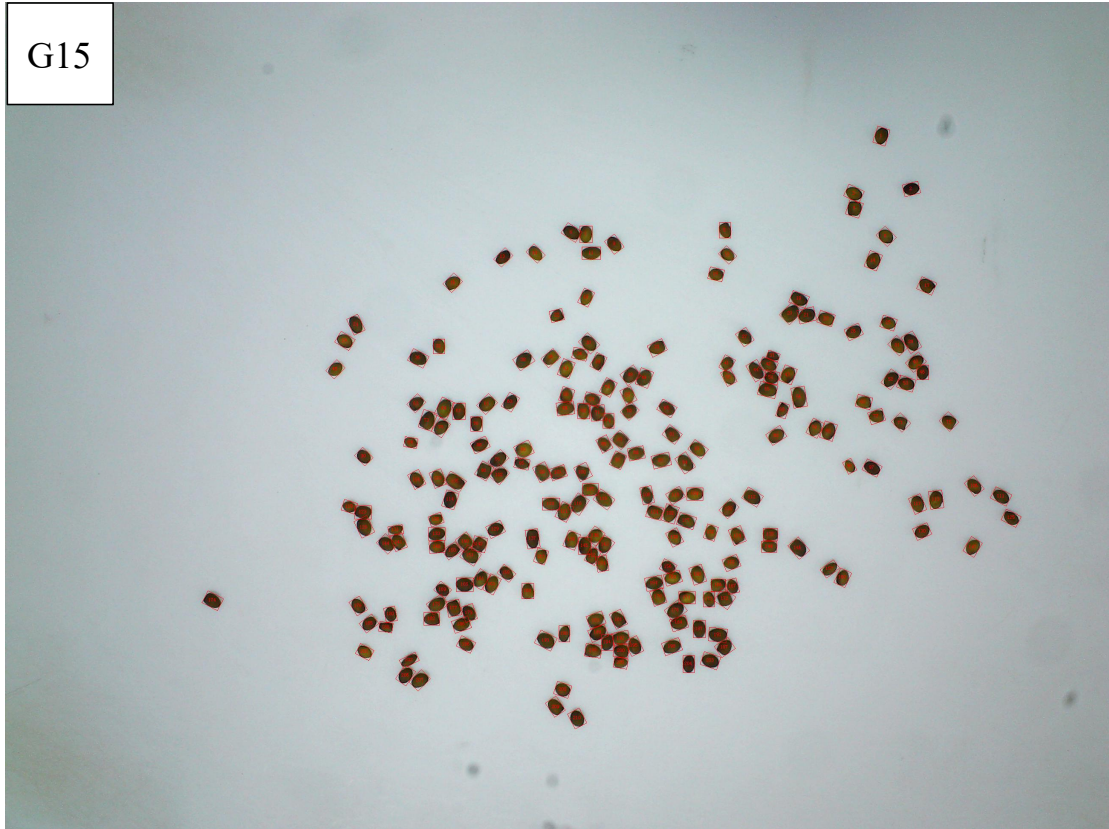

G16

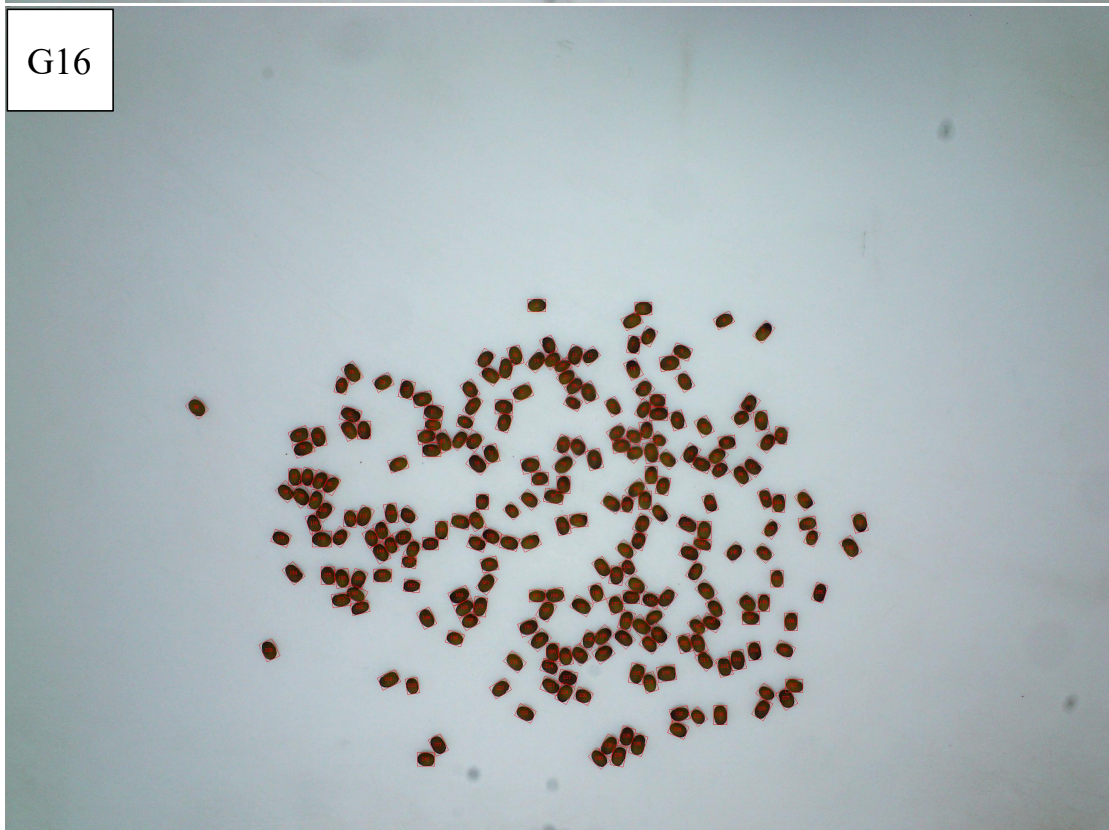

G17

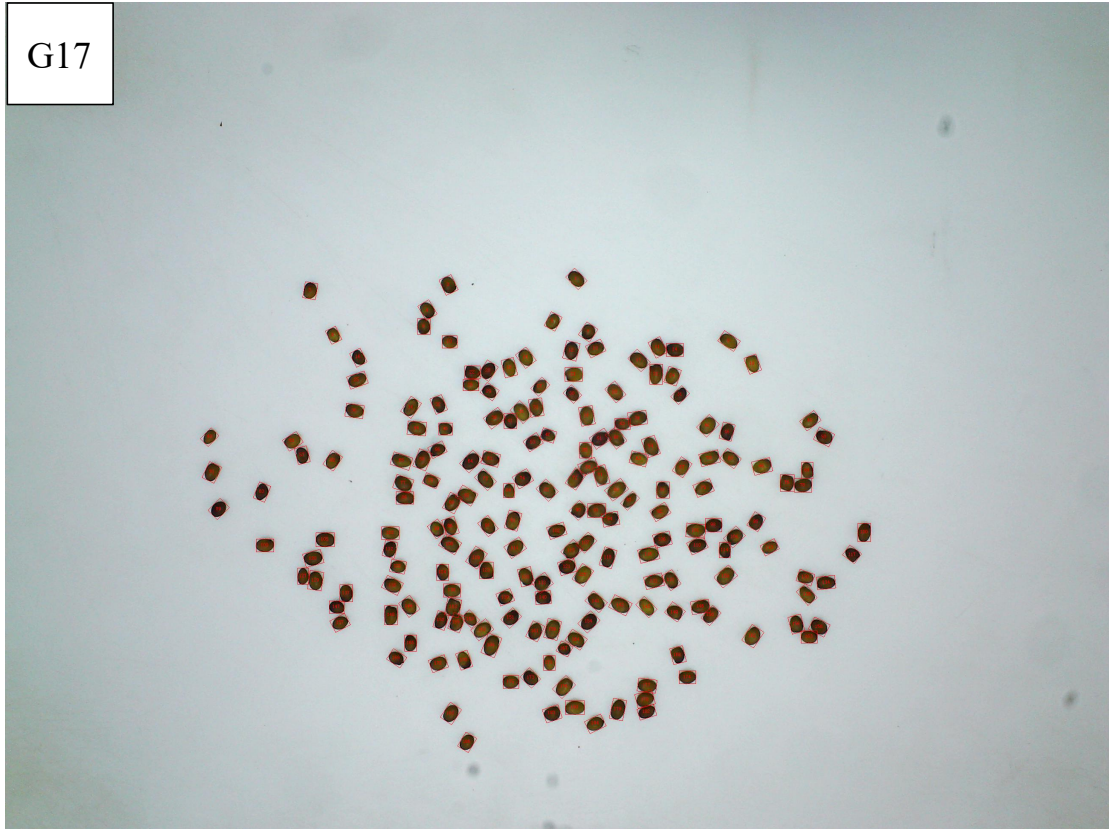

G18

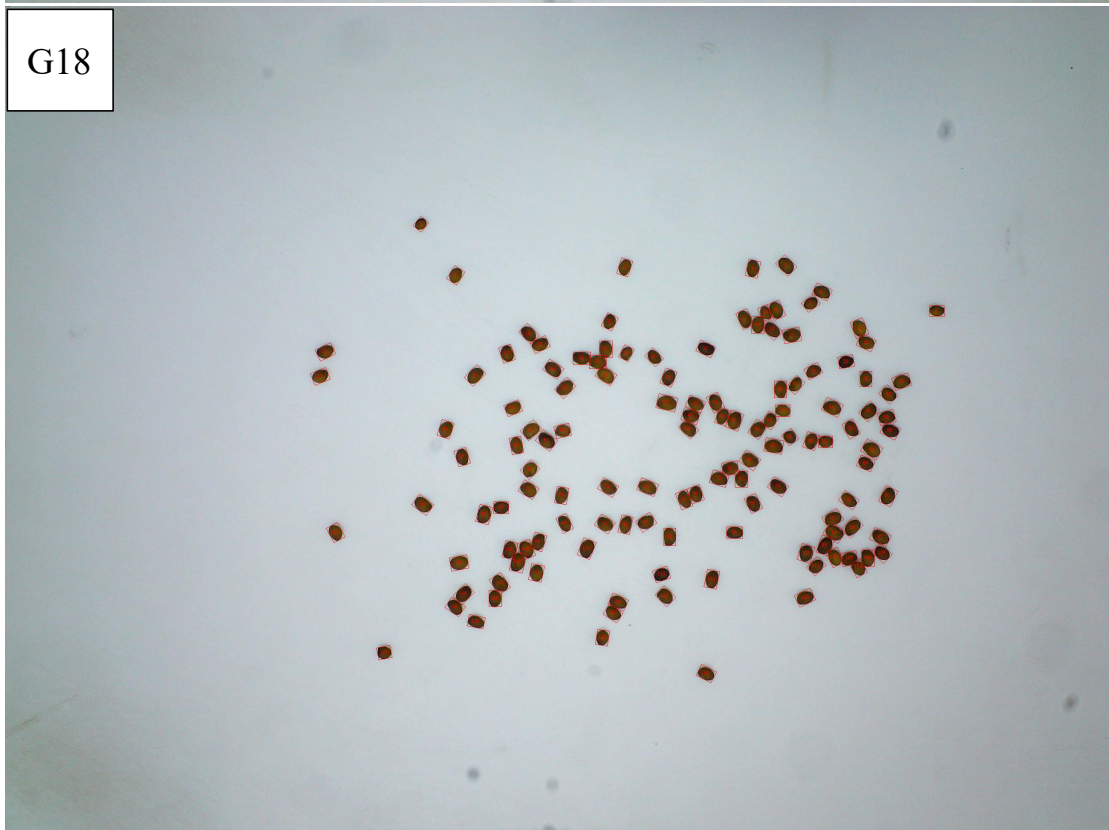

G19

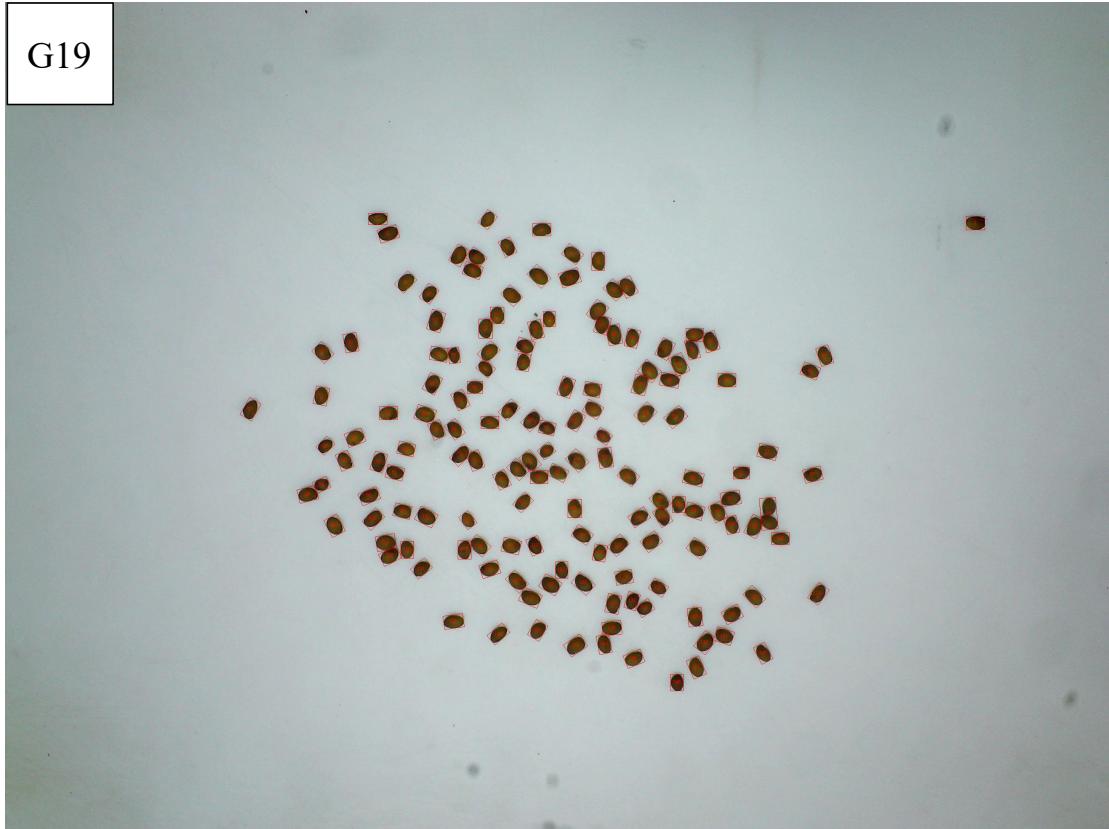

G20

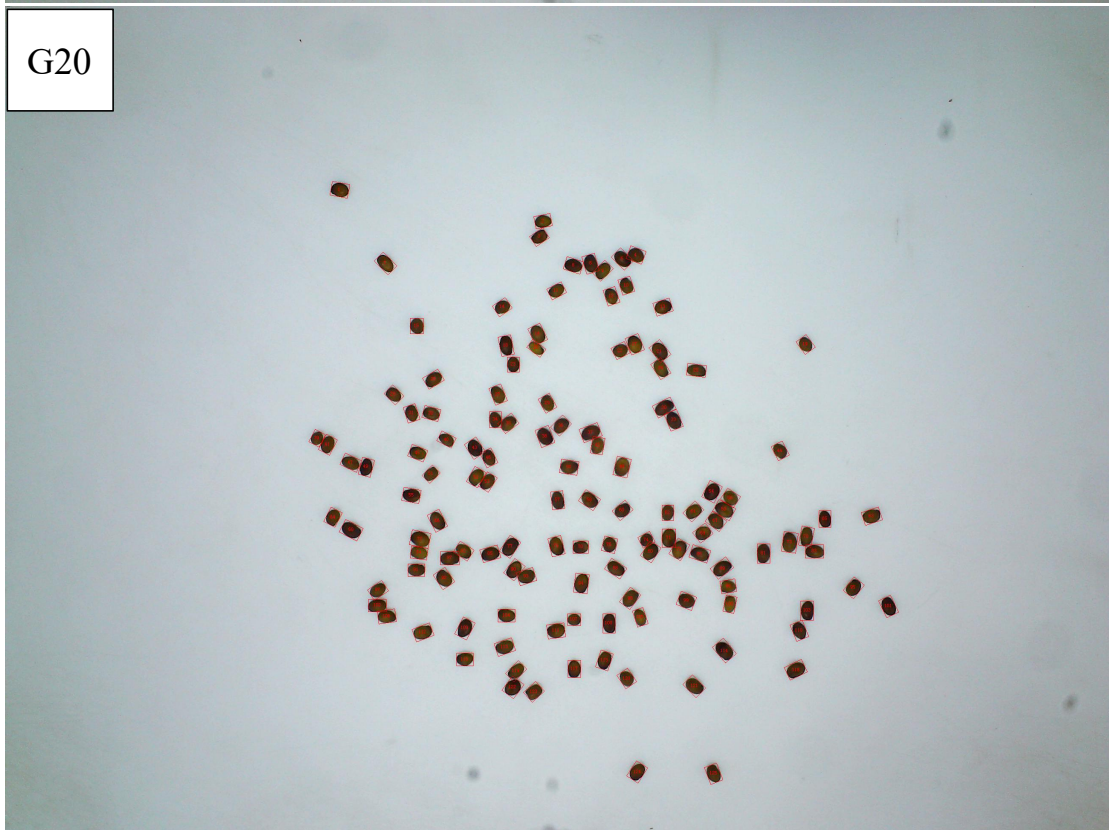

G21

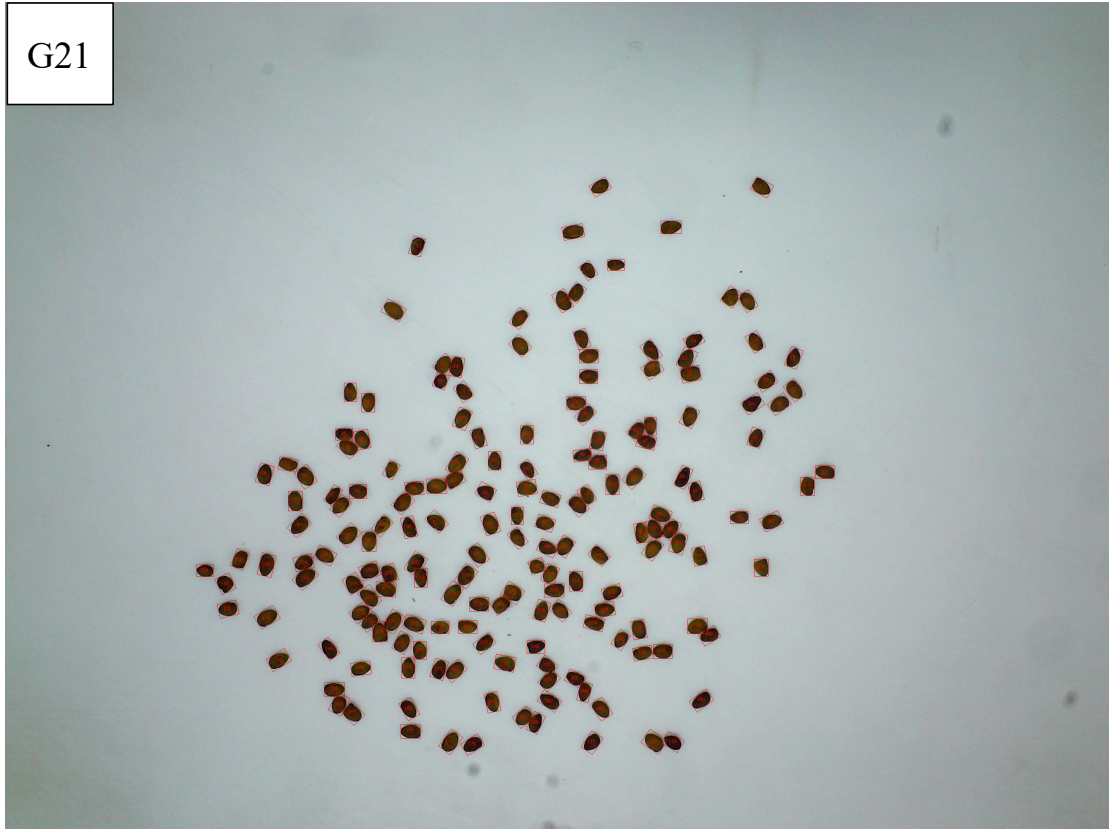

G22

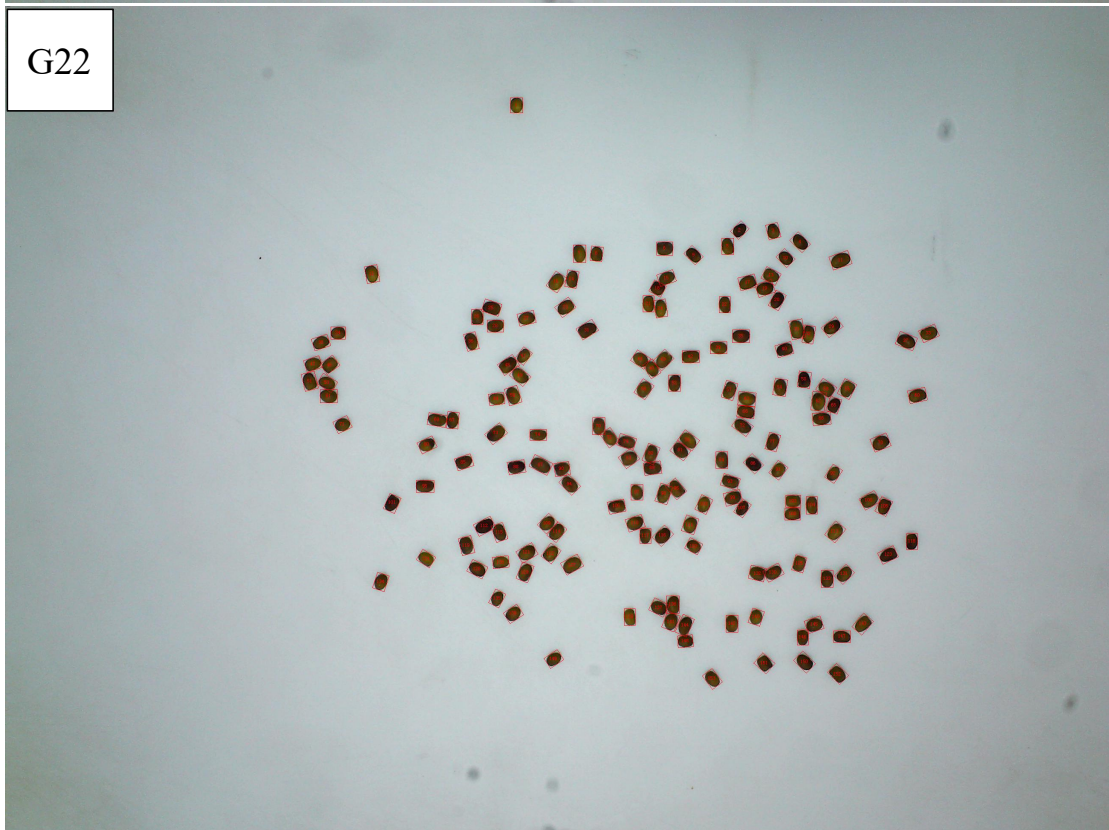

Supplement: Supplementary Table 3 — 22 bioclimatic variables in the 10 populations of S. alopecuroides. [file DataSheet2.zip › Supplementary file 1/TSBL.pdf]

G25

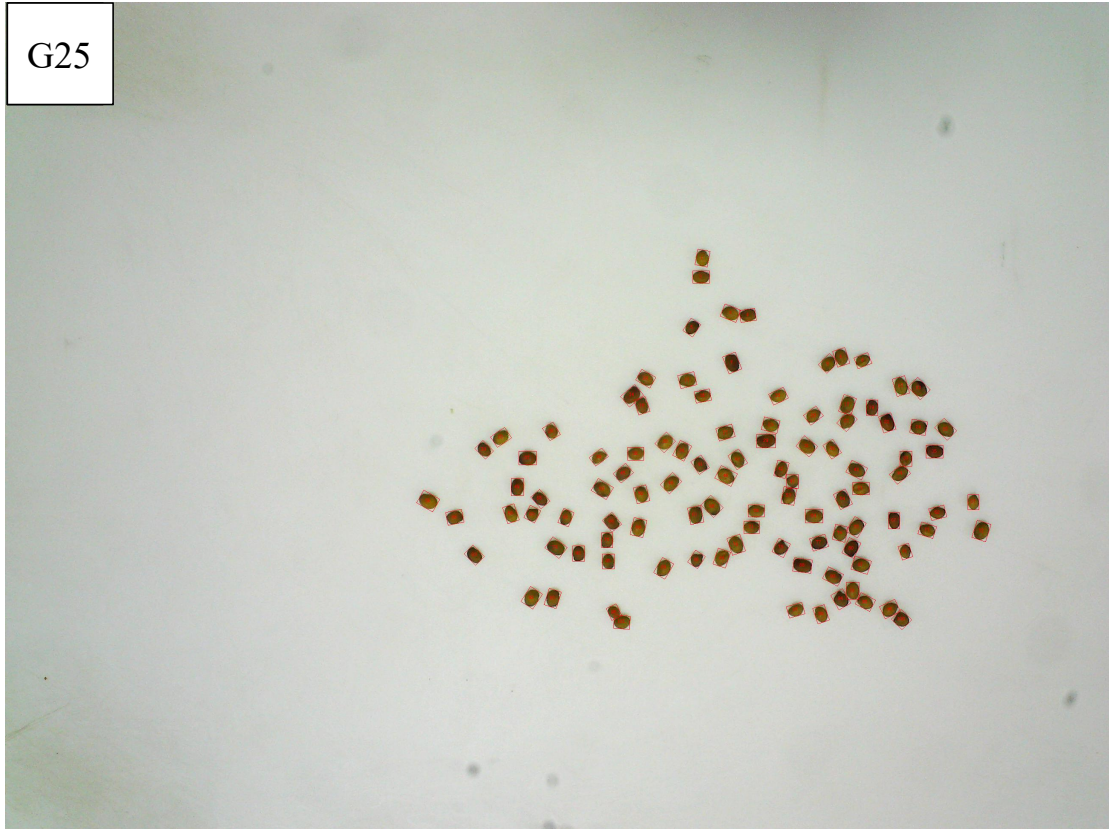

G26

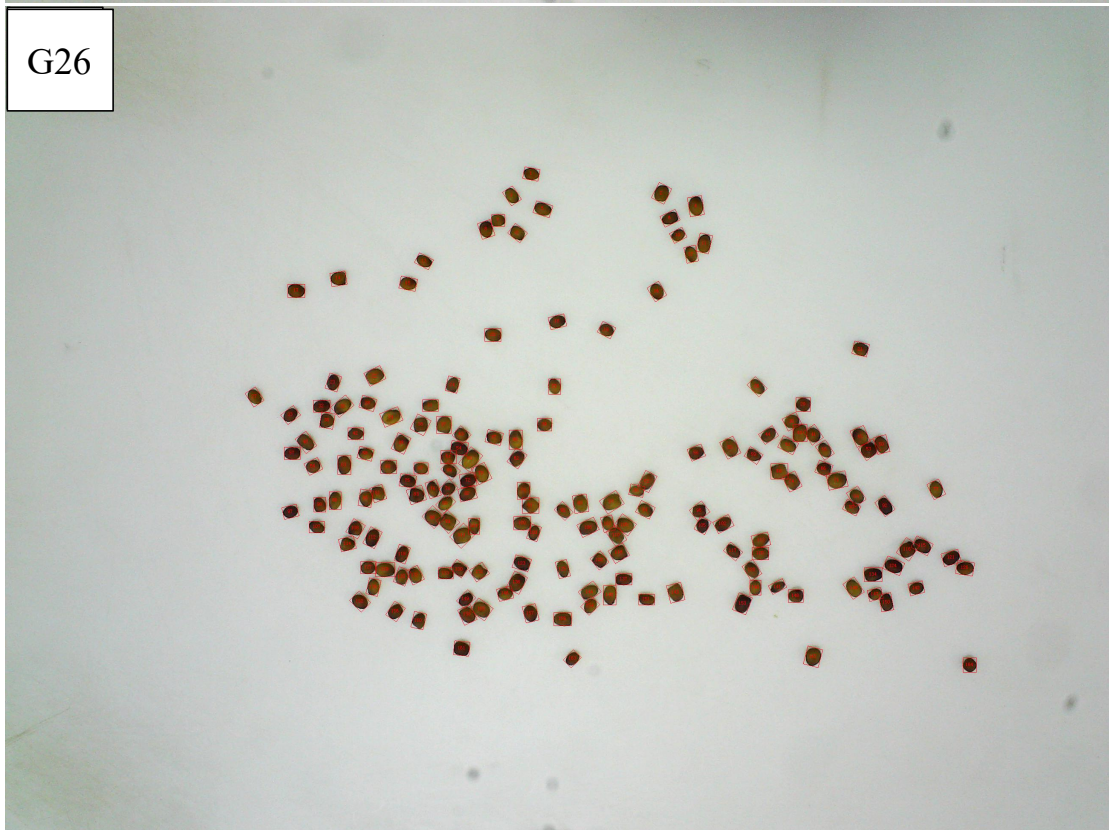

G27

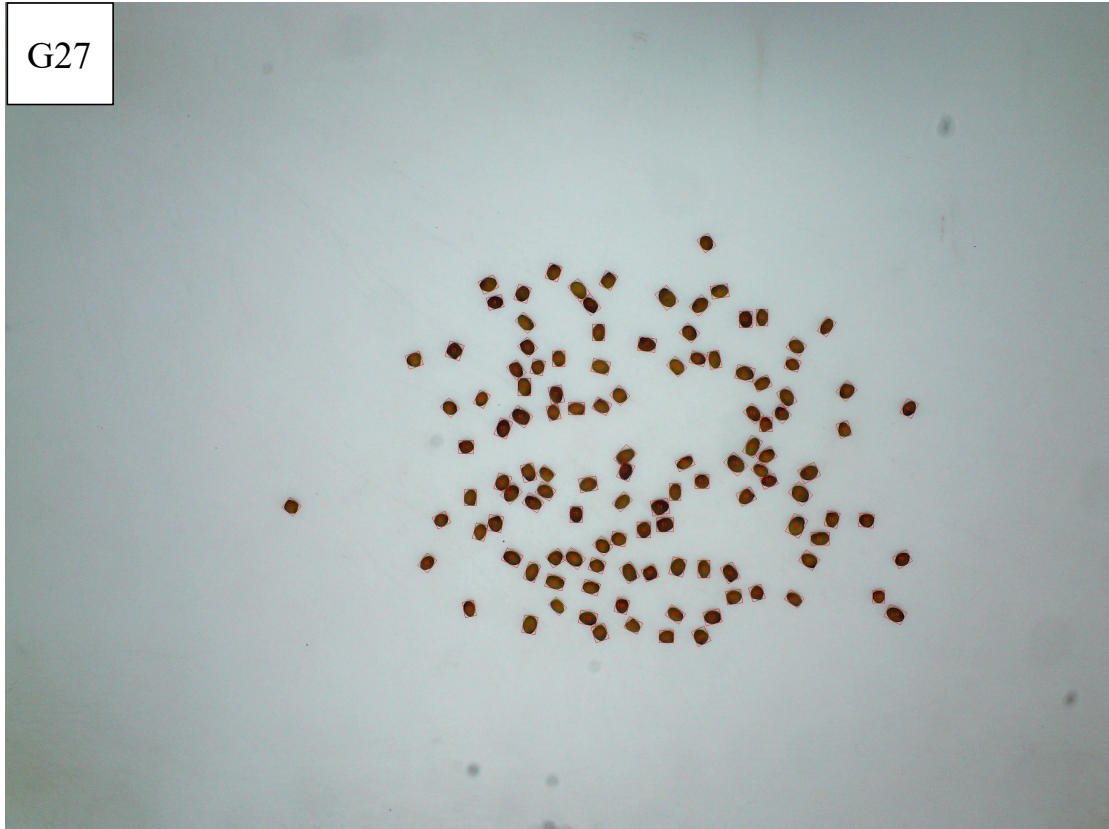

G31

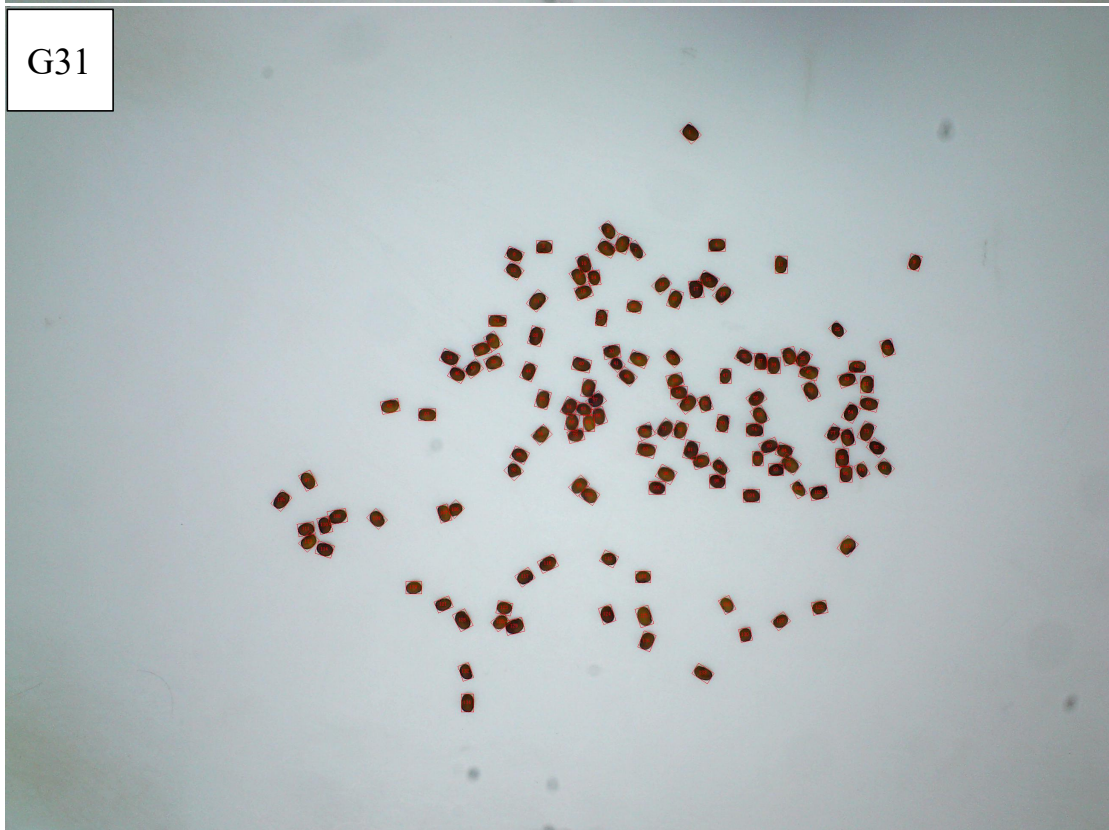

G32

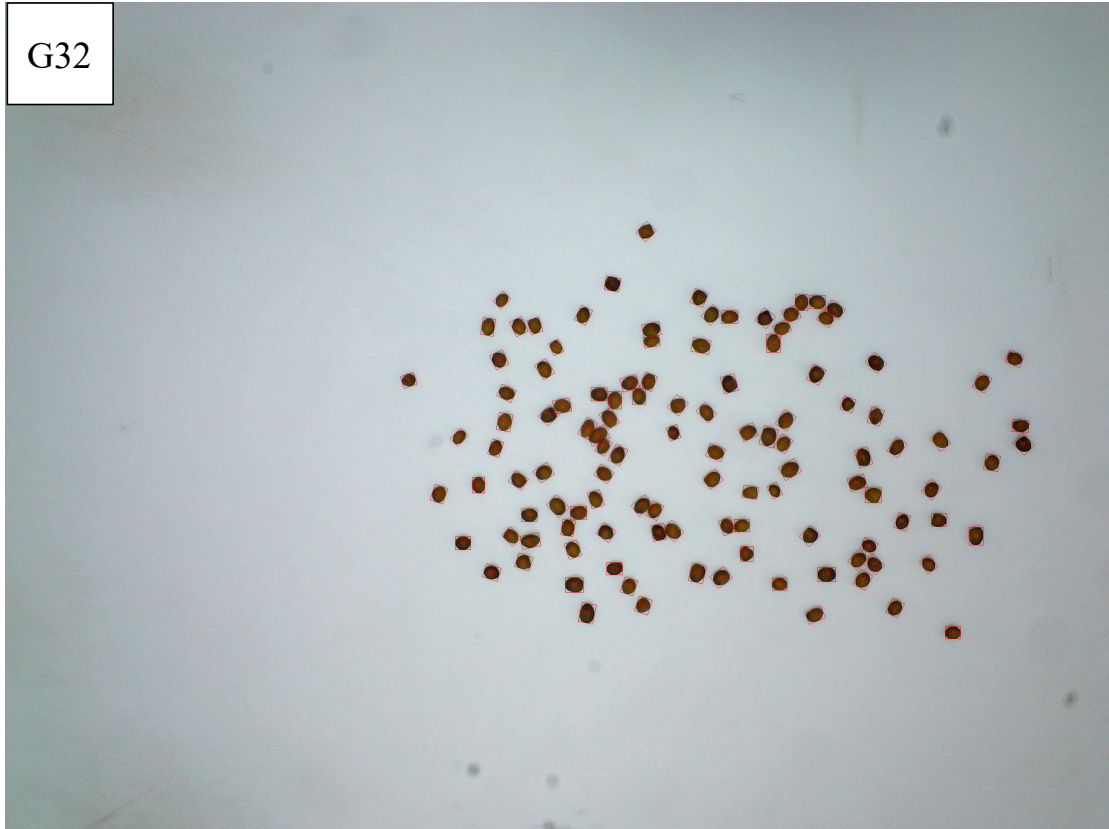

G33

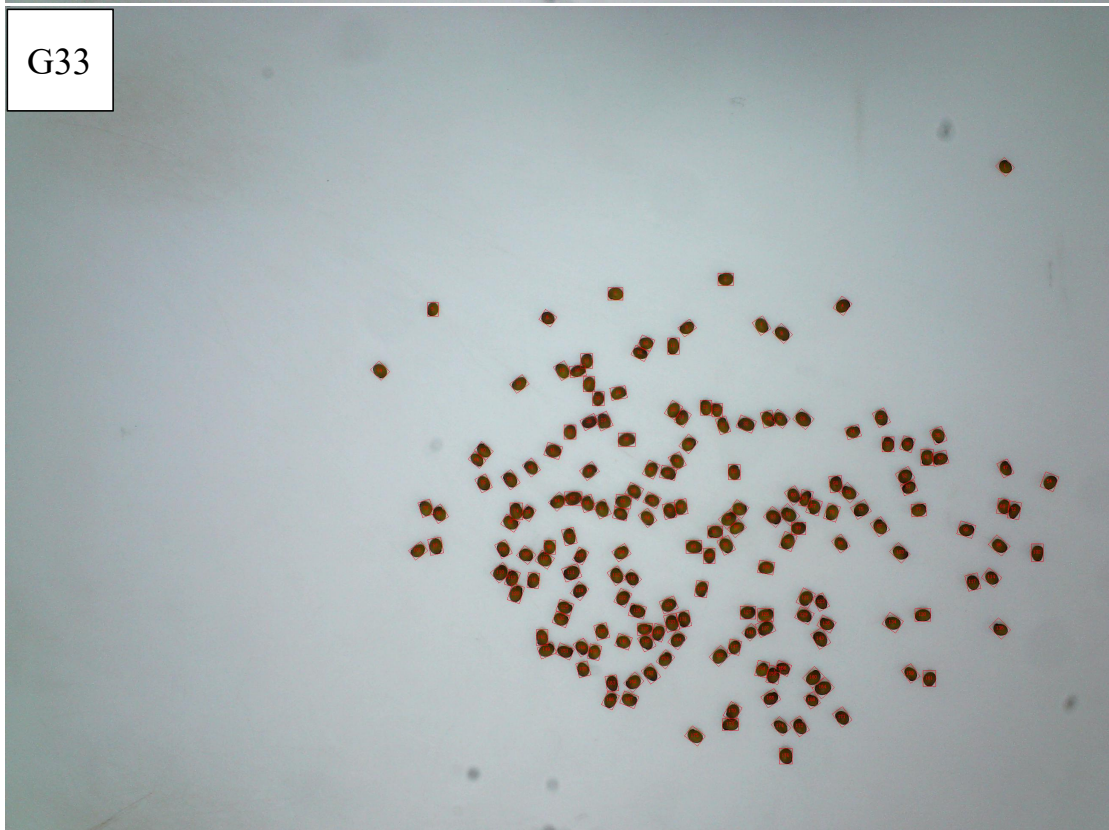

G34

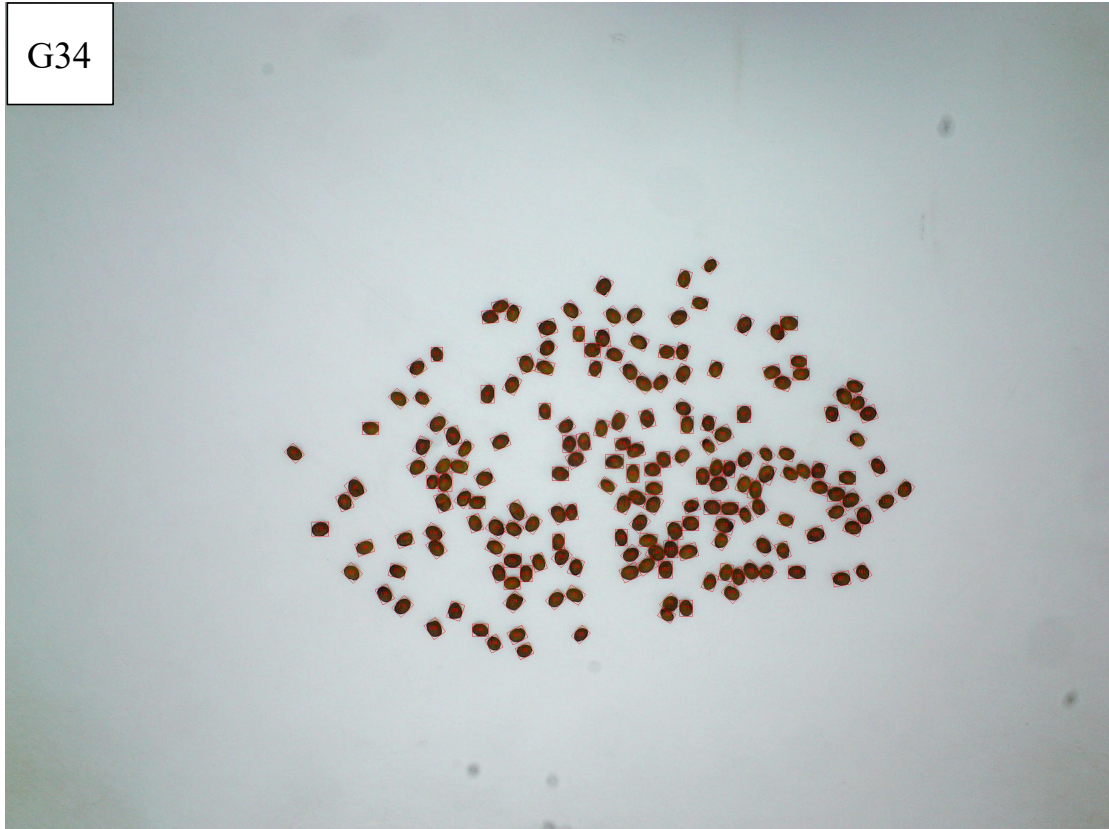

G35

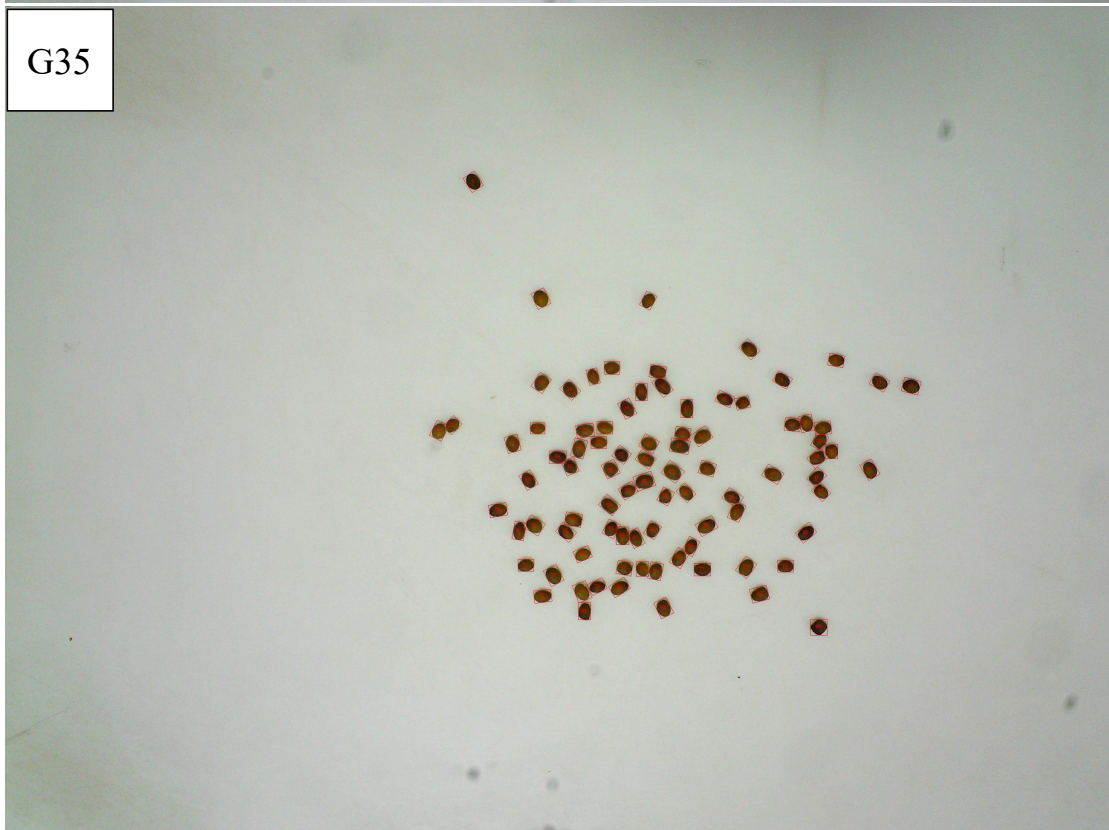

G36

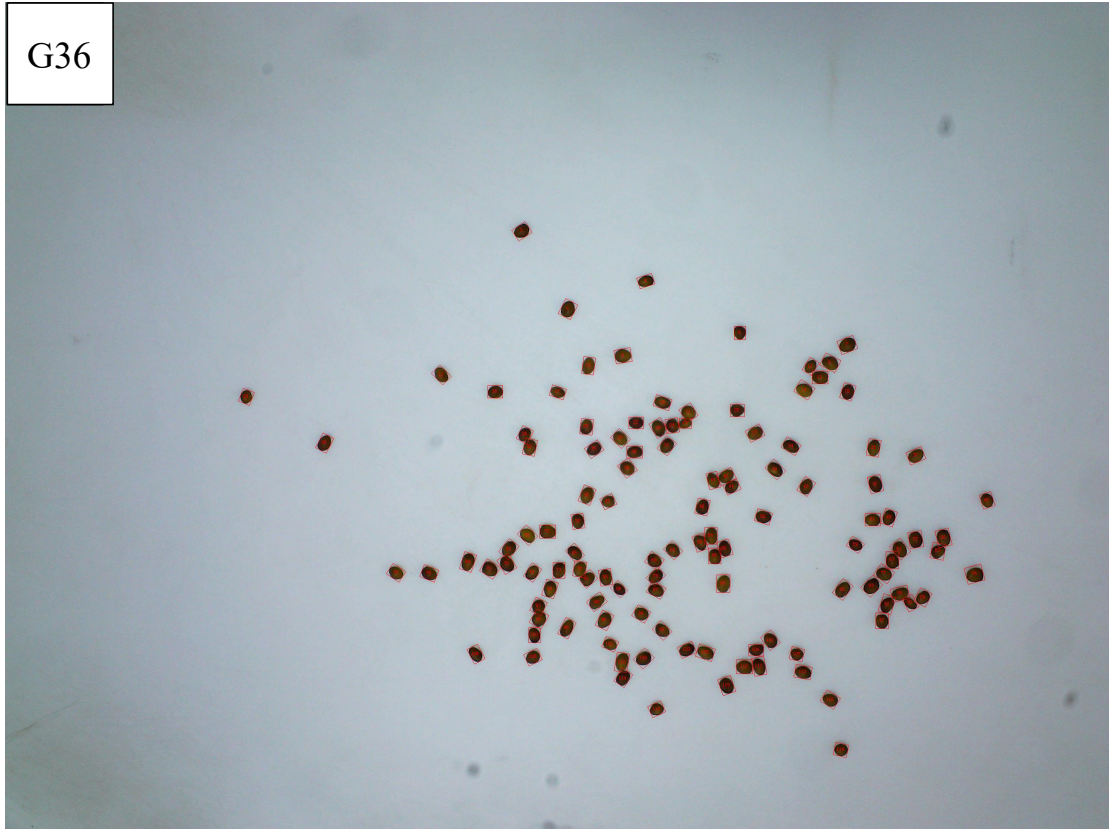

G45

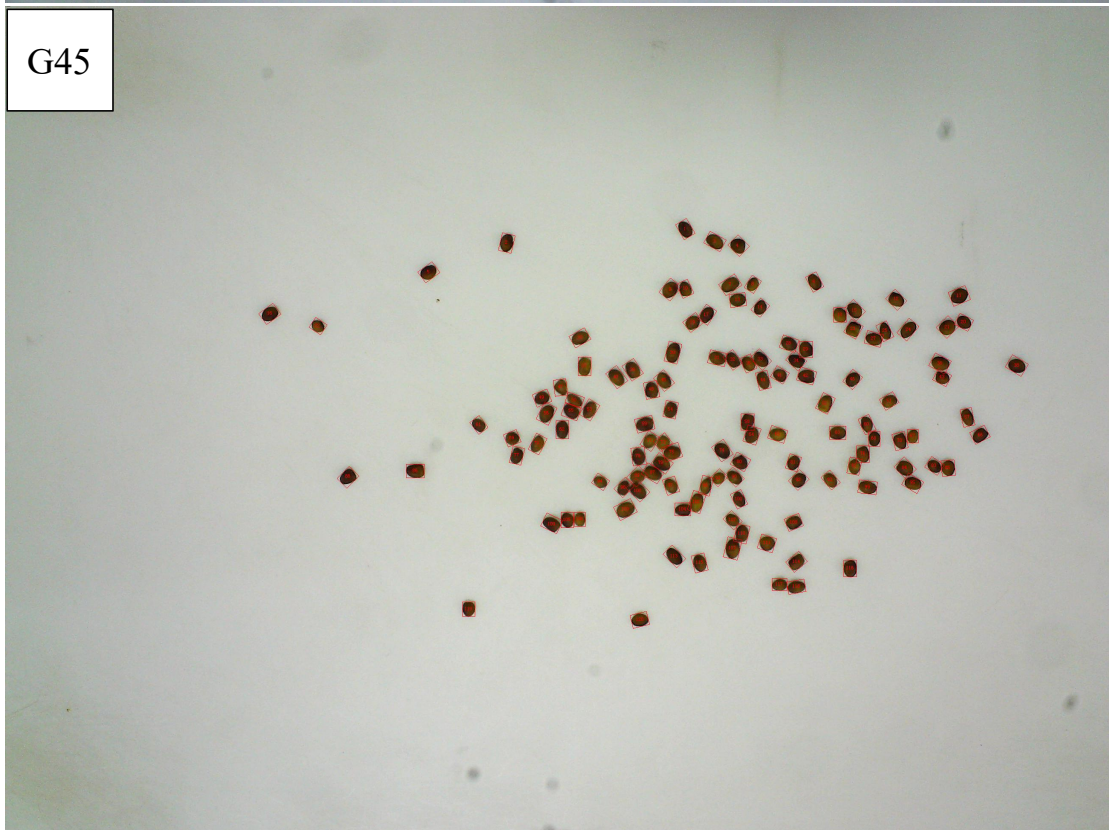

Supplement: Supplementary Table 3 — 22 bioclimatic variables in the 10 populations of S. alopecuroides. [file DataSheet2.zip › Supplementary file 1/TSNL.pdf]

G1

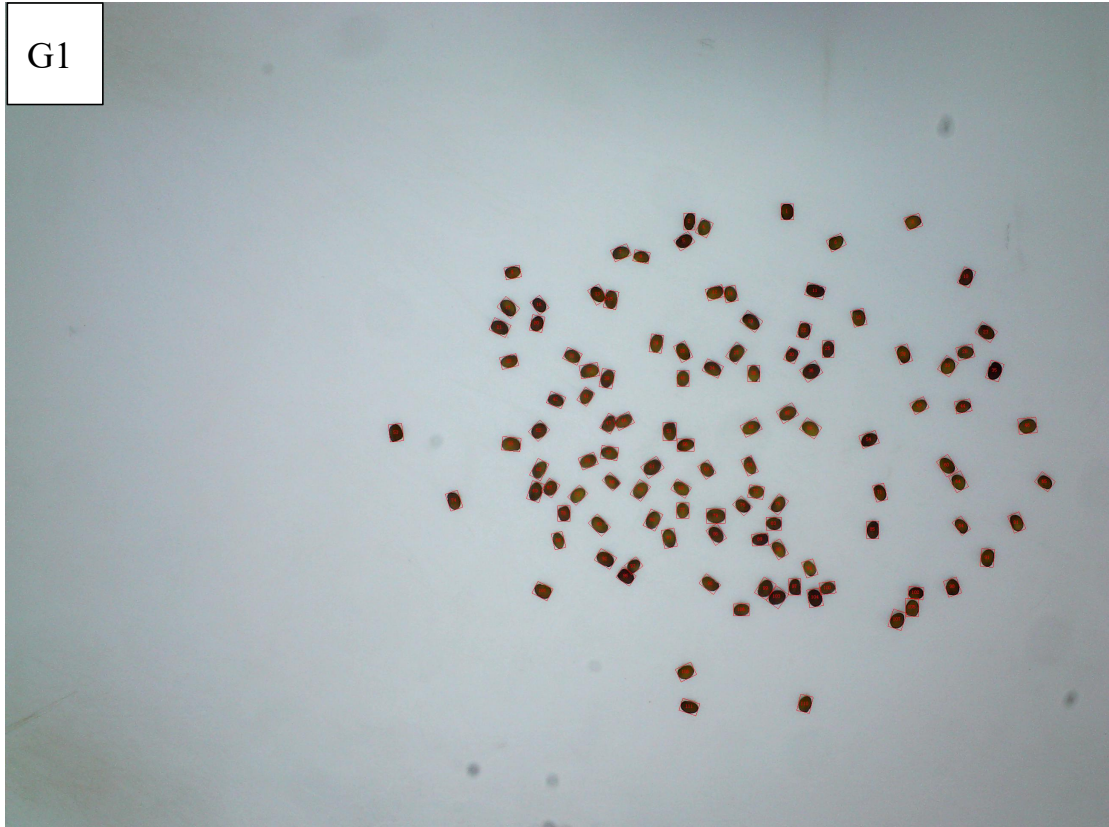

G2

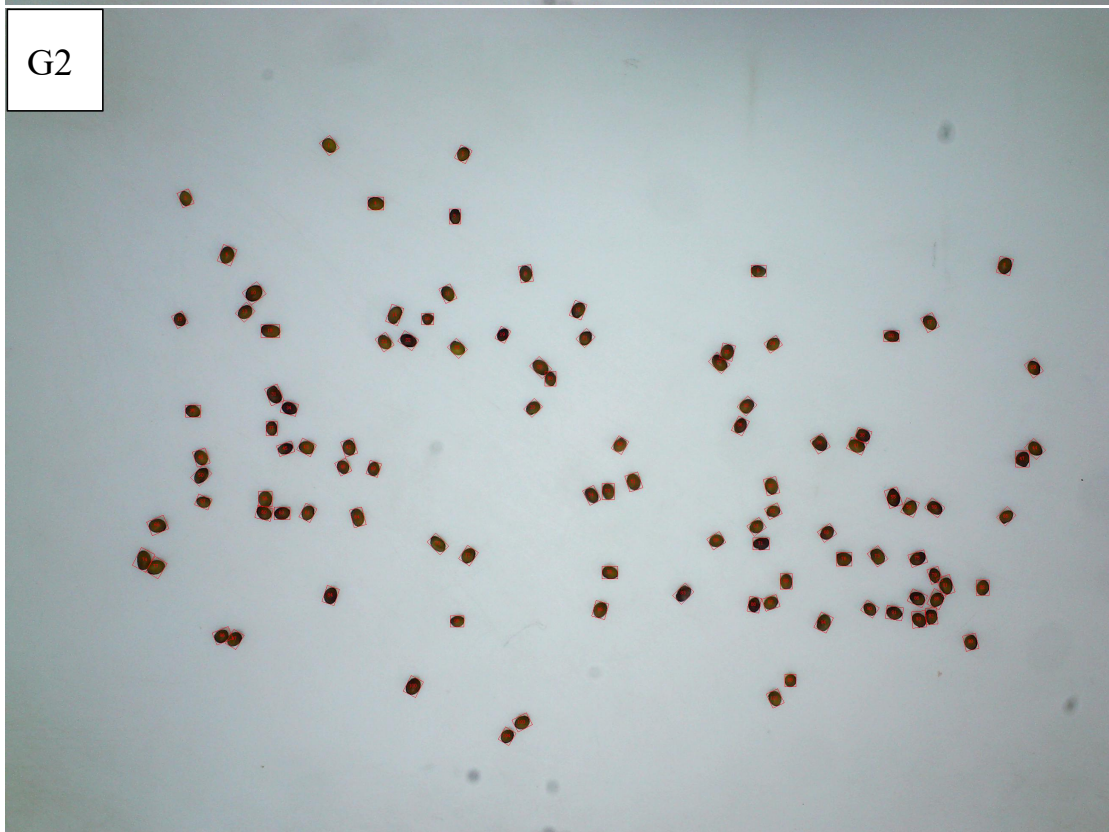

G3

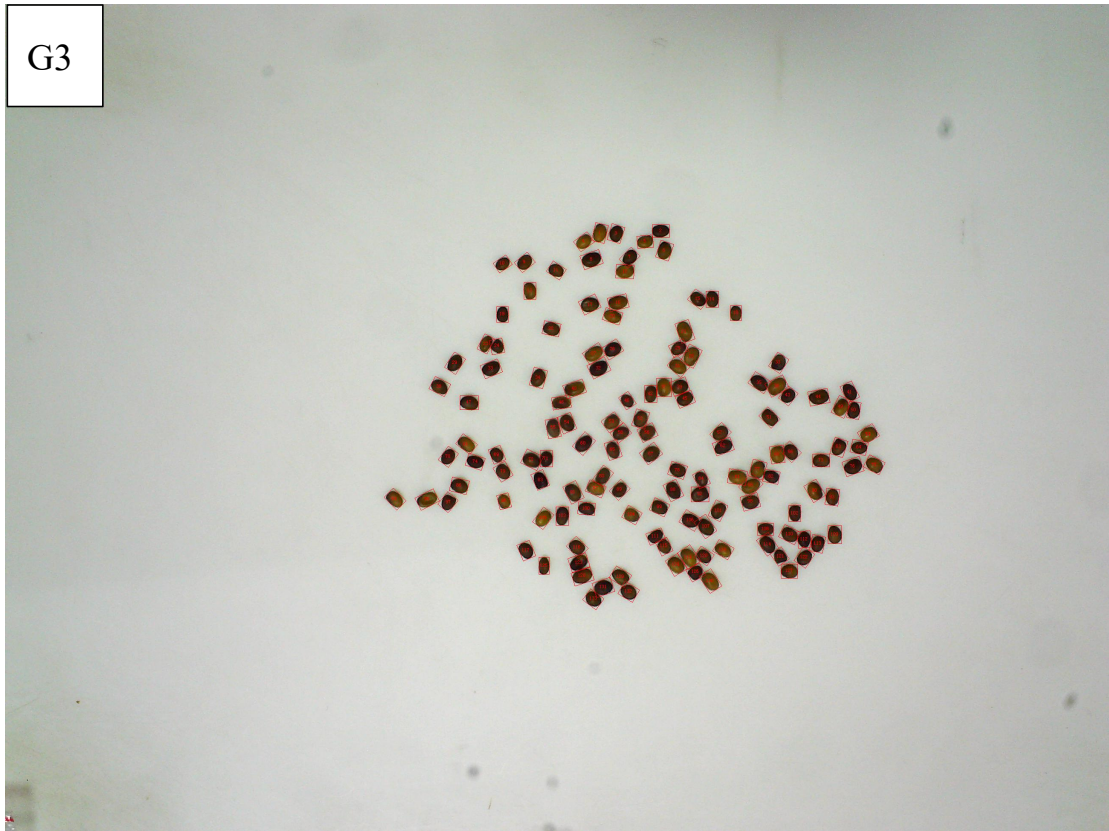

G4

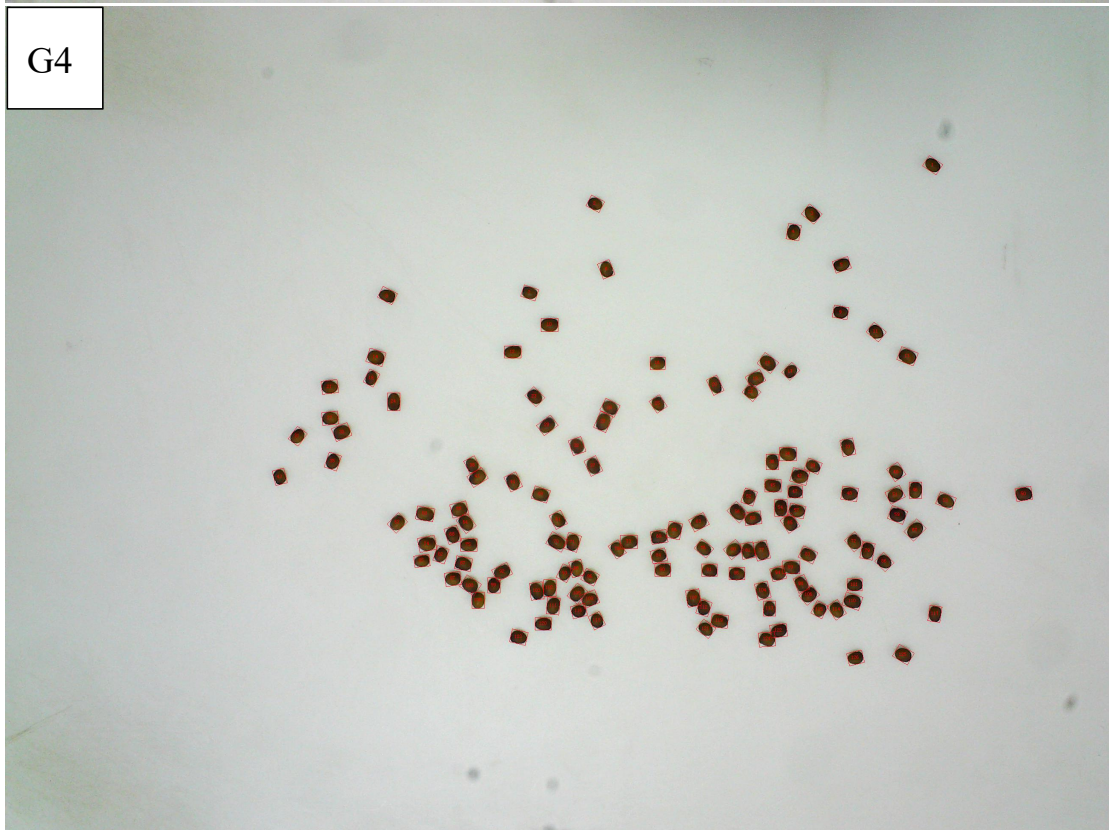

G5

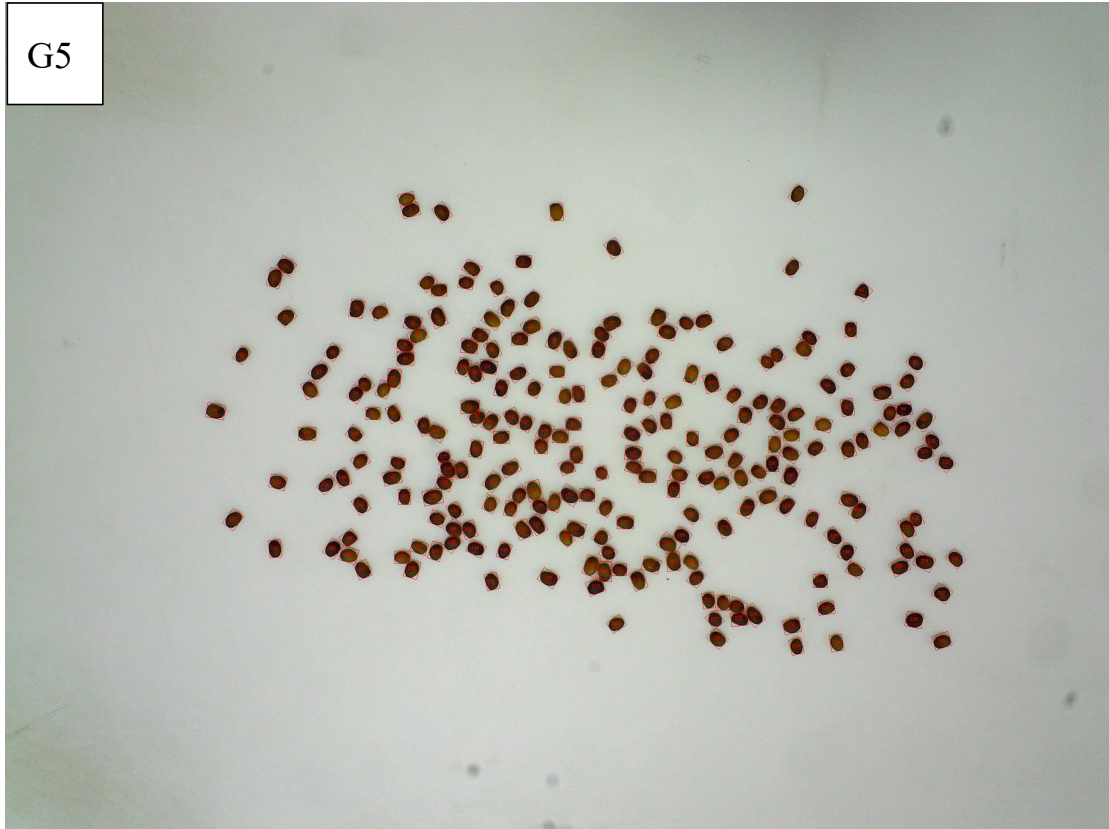

Supplement: Supplementary Table 3 — 22 bioclimatic variables in the 10 populations of S. alopecuroides. [file DataSheet2.zip › Supplementary file 1/YLHG.pdf]
